# Identification of eight QTL controlling multiple yield components in a German multi-parental wheat population, including *Rht24, WAPO-A1*, *WAPO-B1* and genetic loci on chromosomes 5A and 6A

**DOI:** 10.1007/s00122-021-03781-7

**Published:** 2021-03-12

**Authors:** Beatrice Corsi, Lia Obinu, Camila M. Zanella, Saverio Cutrupi, Rob Day, Manuel Geyer, Morten Lillemo, Min Lin, Lorenzo Mazza, Lawrence Percival-Alwyn, Melanie Stadlmeier, Volker Mohler, Lorenz Hartl, James Cockram

**Affiliations:** 1 NIAB, 93 Lawrence Weaver Road, Cambridge, CB3 0LE UK; 2 Department of Agriculture, University of Sassari, Viale Italia, 07100 Sassari, Italy; 3 University of Messina, Messina, Italy; 4 Bavarian State Research Center for Agriculture, Institute for Crop Science and Plant Breeding, 85354 Freising, Germany; 5 Norwegian University of Life Sciences (NMBU), P.O. Box 5003, NO-1432 Ås, Norway; 6 Present Address: Saatzucht Donau GesmbH and Co KG, Mendelweg 1, 4981 Reichersberg, Austria

## Abstract

**Key message:**

Quantitative trait locus (QTL) mapping of 15 yield component traits in a German multi-founder population identified eight QTL each controlling ≥2 phenotypes, including the genetic loci *Rht24*, *WAPO-A1* and *WAPO-B1*.

**Abstract:**

Grain yield in wheat (*Triticum aestivum* L.) is a polygenic trait representing the culmination of many developmental processes and their interactions with the environment. Toward maintaining genetic gains in yield potential, ‘reductionist approaches’ are commonly undertaken by which the genetic control of yield components, that collectively determine yield, are established. Here we use an eight-founder German multi-parental wheat population to investigate the genetic control and phenotypic trade-offs between 15 yield components. Increased grains per ear was significantly positively correlated with the number of fertile spikelets per ear and negatively correlated with the number of infertile spikelets. However, as increased grain number and fertile spikelet number per ear were significantly negatively correlated with thousand grain weight, sink strength limitations were evident. Genetic mapping identified 34 replicated quantitative trait loci (QTL) at two or more test environments, of which 24 resolved into eight loci each controlling two or more traits—termed here ‘multi-trait QTL’ (MT-QTL). These included MT-QTL associated with previously cloned genes controlling semi-dwarf plant stature, and with the genetic locus *Reduced height 24* (*Rht24*) that further modulates plant height. Additionally, MT-QTL controlling spikelet number traits were located to chromosome 7A encompassing the gene *WHEAT ORTHOLOG OF APO1* (*WAPO-A1*), and to its homoeologous location on chromosome 7B containing *WAPO-B1*. The genetic loci identified in this study, particularly those that potentially control multiple yield components, provide future opportunities for the targeted investigation of their underlying genes, gene networks and phenotypic trade-offs, in order to underpin further genetic gains in yield.

**Supplementary Information:**

The online version contains supplementary material available at 10.1007/s00122-021-03781-7.

## Introduction

Bread wheat (*Triticum aestivum* L.) is one of the world’s most widely cultivated crops, accounting for more than 20% of human calorific intake (FAO 2017). Accordingly, wheat production is a key component underpinning global food security. Wheat grains develop within the inflorescence, more commonly termed the ‘spike’ or ‘ear’, located at the apex of a long stem called a tiller. The wheat ear is composed of a central rachis along which branching structures called the rachilla each bear multiple florets that form the spikelet. Each ear typically contains ~ 20 spikelets, which are arranged sequentially on alternate sides of the rachis axis with a terminal spikelet at the apex (Murai et al. 2002). The rachilla meristem is indeterminant, and around 4–5 florets are arranged sequentially along alternating sides of the rachilla axis, with the florets referred to from the spikelet base to tip as position G1, G2, G3, etc. Ultimately, grain yield represents the culmination of many genetic, developmental, and environmental interactions with the coordination of plant growth and development across the growing season, and is under complex genetic control—although it can often show high heritability (e.g. Muqaddasi et al. 2019). Yield can be largely partitioned into three major components: grain weight/size and shape, grain number per spike and spike number per unit area (Gegas et al. 2010). Evidence suggests that improvements in grain number per ear (itself a combination of the number of spikelets, and floret fertility) have played an important role in the increased yields attained via modern wheat breeding (e.g. Calderini et al. 1995; Würschum et al. 2018). Similarly, grain size has also increased, with for example a recent study of Chinese wheat varieties indicating that breeding has resulted in increased grain size predominantly at positions G1, G2 and G3 (Feng et al. 2018). These major yield components can be further subdivided into constituent traits, e.g. grain size = grain morphometric traits, carpel (unfertilized female organ) size, rate of grain filling, cell expansion rate, and cell number (Brinton and Uauy 2019). They are also influenced by other factors such as accumulation and transport of photosynthetic products, flag leaf size and angle, plant height, overall biomass and flowering time, and the interaction of all these factors with the environment. Furthermore, wheat processing traits that affect milling performance (e.g. grain shape, size, density and uniformity) are also critical for flour yield. The inter-dependent roles of the ‘source’ (e.g. leaves) and ‘sink’ (e.g. grain) tissues in building yield across the lifecycle of a wheat plant are complex, and trade-offs between yield components are the norm. For example, two varieties with the same overall grain yield could be a result of more grains per ear in one variety, or more tillers and less grain per ear in another. Similarly, the strong negative correlation between grain yield and protein content is a well-recognized trade-off in wheat, and other crops (Simmonds 1995; Mosleth et al. 2020). Such compensatory phenotypic effects, along with the large, complex polyploid nature of the 17 Gb wheat genome (IWGSC 2018)—within which homoeologous gene copies buffer the effects of mutation at any single gene—make the physiological and genetic study of yield and yield components potentially more challenging than in diploids (Brinton and Uauy 2019). For example, numerous natural genetic variants controlling yield components have been map-based cloned in the diploid cereal crop rice (*Oryza sativa* L.) (for a review of rice grain size genes, see Li et al. 2018a), and these tend to account for ~ 10–40% of the phenotypic variation. In contrast, while exceptions do exist, most notably a locus on chromosome 7A controlling spikelet number per ear (Muqaddasi et al. 2019; Kuzay et al. 2019; Voss-Fels et al. 2019), natural variation at wheat genetic loci controlling yield and yield component traits in bread wheat typically control just ~ 2–10% of the variation (reviewed by Brinton and Uauy, 2019). This observation is likely due to the compensatory effects of homoeologous genes in polyploid wheat. However, this also provides opportunities for molecular-assisted improvement in hexaploid wheat if characterized alleles at homoeologous genetic loci or genes can be combined in a single genetic background to overcome functional redundancy. One route to achieve this aim is to identify quantitative trait loci (QTL) controlling a trait of interest, and ultimately to characterize the genes and gene variants that underly them. Toward this aim, numerous studies investigating the genetics and/or physiology of grain yield (e.g. Rustgi et al. 2013; Garcia et al. 2019), grain size (e.g. Gegas et al. 2010; Xie et al. 2015; Brinton et al. 2017), carpel size (Xi et al. 2015) and grain three-dimensional shape (Hughes et al. 2019) have been undertaken, as well as analyses of key traits which impact yield such as flowering time (e.g. Griffiths et al. 2009a), height (e.g. Griffiths et al. 2009b), flag leaf size (e.g. Xue et al. 2013; Ma et al. 2020), stomatal conductance and photosynthetic performance (Faralli et al. 2019). However, just two wheat yield component QTL have been cloned or fine-mapped to the genic level to date. The first, *Grain Number Increase 1* (*GNI1*), was identified as a QTL in a cross between a tetraploid durum wheat (*T. durum*) cultivar and a *T. durum* line which contained an introgression from wild tetraploid emmer wheat (*T. turgidum* ssp. *dicoccoides*) (Sakuma et al. 2019). The underlying gene, *GNI-A1*, encodes a homeodomain leucine zipper class I (HD-Zip 1) transcription factor orthologous to the barley inflorescence architecture gene *Six-rowed spike 1* (*VRS1*) that controls the fertility of lateral florets on the barley spikelet (Komatsuda et al. 2007). *GNI-A1* acts to repress floral fertility in durum wheat, with the reduced function allele resulting in increased numbers of fertile florets per spikelet. The *GNI-A1* reduced function allele increases grain weight at spikelets located predominantly at the basal and central regions of the ear (Sakuma et al. 2019; Golan et al. 2019), as well as at specific grain position within the spikelet (G1, G2, G3). Subsequent resequencing of *GNI-A1* in a panel of European bread wheat found a similar reduced function allele to be present in around a third of the varieties surveyed and was associated with higher grain number per ear (Sakuma et al. 2019). More recently, fine-mapping of a QTL on chromosome 7A controlling spikelet number per ear in bread wheat has resulted in the identification of *WHEAT ORTHOLG OF APO1* (*WAPO1*) as the likely underlying gene (Kuzay et al. 2019; Muqaddasi et al. 2019). *WAPO1* underlies a wheat yield component QTL of unusually large effect, explaining up to 23% of the phenotypic variation (Kuzay et al. 2019; Muqaddasi et al. 2019; Voss-Fels et al. 2019). It encodes an F-box protein orthologous to the rice gene *ABBERANT PANICLE ORGANISATION 1* (*APO1*) which controls rice spikelet number (Ikeda et al. 2007), and is homologous to *UNUSUAL FLORAL ORGANS* (*UFO*) that regulates arabidopsis floral organ identity (Wilkinson et al. 1995; Samach et al. 1999).

To date, most studies of yield and yield components have been undertaken in either bi-parental or association mapping populations, each of which has their own inherent pros and cons (reviewed by Cockram and Mackay 2018). For example, bi-parental populations are limited in the genetic diversity that the two founders can capture, while the need to account for genetic population substructure can reduce the power to detect QTL in association mapping panels. The relatively recent development of multi-founder populations in many plant species (reviewed by Scott et al. 2020a) provides alternative population types with which to investigate the genetics of target traits. Here we use an eight founder multiparent advanced generation inter-cross (MAGIC) population: the Bavarian MAGIC winter wheat population (BMWpop) (Stadlmeier et al. 2018). The population consists of 394 F_6:8_ recombinant inbred lines derived from eight founders: Event, BAYP4535, Ambition, Firl3565, Format, Potenzial, Bussard and Julius. With the exception of the Danish variety ‘Ambition’, the founders represent breeding lines and released varieties developed by German wheat breeding companies, and the population has been estimated to capture 71.7% of the allelic diversity available in the German wheat breeding gene pool (Stadlmeier et al. 2018). Although the BMWpop has previously been used to investigate the genetics of wheat disease resistance (Stadlmeier et al. 2019; Lin et al. 2020), it has yet been used to study yield components. With the aim of identifying yield component QTL relevant to north-western European wheat cultivation, we grew the BMWpop in three trials across two countries and phenotyped 15 traits. Subsequent genetic analysis resulted in the identification of 34 replicated QTL, of which 24 were found to resolve into eight genetic loci each controlling two or more traits.

## Methods

### Wheat germplasm and field trials

Details of the characteristics of the BMWpop founders and population are described by Stadlmeier et al. (2018). Briefly, it consists of 394 recombinant inbred lines created from eight winter wheat varieties using a simplified mating design, which included an additional eight-way inter-cross step to give the same results in terms of the levels of missing founder assignments and number of recombination events as a MAGIC design with all 210 possible four-way crosses. The BMWpop genetic linkage map consists of 5,435 single nucleotide polymorphism (SNP) markers distributed over 2,804 loci and spanning 5,230 cM (Stadlmeier et al. 2018). Here, all 394 lines of the BMWpop were grown with two replicate plots per line in three trials: two trials in the United Kingdom (UK, in years 2017 and 2018), and one in Germany in 2018. Trials were sown in the autumn and grown to full maturity the following summer and were cultivated under standard local agronomic practices, but without the application of fungicides and growth regulators in the UK. The two UK field trials were conducted at NIAB, Cambridge, United Kingdom (UK) (Lat. 52.242931, Long. 0.097802). The trials consisted of 394 recombinant inbred lines in two replications each, and the eight founders in three replications each, plus three additional controls (the cultivars Arina, Jenga and Tarso) in four replications each. Trial design was undertaken in R (R Core Team, 2015) using the package Blocks Design v2.8. Each trial was arranged in two randomized, complete replicates, each of 13 blocks for a total of 836 plots, with each plot consisting of two 1-m rows. The German trial was conducted at Frankendorf (Lat. 48.348091, Long. 11.977043) and consisted of the 394 recombinant inbred lines and founders in two replications each, with the exception of the founder Julius which was replicated nine times, as well as the check variety RGT Reform in seven replications. The trial was composed of 860 plots in an alpha lattice design with two replications and 43 incomplete blocks per replication. Three sub blocks were built based on plant height. Randomization of the trial was performed using the R package Agricolae version 1.3–2 (de Mendiburu 2009). Plot sizes were 1.5 m x 3 m. Subsequently in this manuscript, where numbers are used to indicate BMWpop founders, these are listed as: (1) Event, (2) BAYP4535, (3) Ambition, (4) Firl3565, (5) Format, (6) Potenzial, (7) Bussard, (8) Julius.

### Phenotyping and trial analyses

The trait ‘plant height’ was measured in cm as the height from ground to the top of the spike, excluding awns or scurs. ‘Flowering time’ was scored the date the majority of plants within a plot had fully emerged ears (Zadoks growth stage GS59), and recorded as days from the 1^st^ May. From each plot, ten representative ears were collected at plant maturity for subsequent phenotypic analysis: ‘ear length’ (in mm, measured from the base of the first spikelet to the apex of the last spikelet at the top of the ear, excluding awns or scurs), ‘ear weight’ (g), ‘total number of spikelets’, ‘number of fertile spikelets,’ and ‘number of infertile spikelets’. Subsequently, ear samples from each plot were threshed, cleaned of chaff, and used for grain phenotyping using digital images taken with a MARVIN Grain Analyser (www.gta-sensorik.com). Analysis of the resulting images using MARVIN 5.0 software (GTA Sensorik GmbH), combined with the knowledge of the number of ears from which the grain originated, resulted in the calculation of the following phenotypes: ‘number of seeds per ear’, ‘seed length’, ‘seed width,’ and ‘seed area’. After imaging, grain was weighed (g), allowing ‘thousand grain weight’ (g) to be determined using the MARVIN software. The following additional derived phenotypes were also calculated: ‘function form density’ (thousand grain weight/seed area), ‘seed length/seed width ratio’, and ‘seed weight/ear weight ratio’. Finally, the most prominent fungal diseases present in the UK17 (septoria tritici blotch) and UK18 (yellow rust) trials were scored on the first four leaves at the plot level using a percentage infection scale, following the guide in Supplementary Table 1.

### Statistical analysis and genetic mapping

Summary statistics (mean, median, standard deviation and variance) were calculated using GenStat 19^th^ edition (VSN International). Best linear unbiased estimates (BLUEs) were calculated using a linear mixed approach in REML using GenStat. For each trait in each test environment, histograms of the BLUEs were created using the package ggplot2 (Wickham 2016) in R Studio (RStudio Team 2020). Pearson’s correlation coefficients and paired Wilcoxon signed-rank test were carried out in R Studio using the package Hmisc (Harrell 2019) and plotted using the R package corrplot (Wei and Simko 2017). Heritability was estimated as 1 – *Att* / (2 γg^2^), where γg^2^ is the variance component of the term divided by the residual variance, and *Att* is the average variance for differences between the effects of the term divided by the residual variance (Cullis et al. 2006; Piepho and Möhring 2007).

MAGIC QTL mapping was carried out using the subset of 2,804 SNP markers representing unique positions on the BMWpop genetic map, as described by Stadlmeier et al. (2018). Four QTL analysis approaches were used: (1) SMA (identity-by-state single marker analysis): single marker analysis using the SNP bi-allelic classes using R/lme4 (Bates et al. 2015). (2) IBD (identity by descent): regression against haplotype probability estimates calculated using the ‘mpprob’ function in R/mpMap (Huang and George 2011) implemented in R/qtl (Broman et al. 2003) with a threshold of 0.5. (3) IM (interval mapping): conducted in R/mpMap using R/mpMap haplotype probability estimates. (4) CIM (composite interval mapping): conducted in R/mpMap with 5 (CIM-cov5) or 10 (CIM-cov10) covariates using R/mpMap haplotype probability estimates. For IBS and IBD analyses, multiple-test correction was carried out using the p.adjust function in R, with a threshold of *p* < 0.05. For IM/CIM an empirical *p* threshold of 0.05 was determined for QTL analyses by conducting 100 simulations, using the sim.sigthr function in R. This value, together with a window size of 100 markers, was used to determine QTL peaks using “find.qtl”. All detected QTL were simultaneously fitted in a full model using the function ‘fit.qtl, and QTL retained where *p* < 0.05 and percentage variation explained > 1% in the fitted model. The full model including all detected QTL was fitted to estimate the overall phenotypic variance explained (*R*^2^). IM was used to call QTL, with additional detection using CIM-cov5, CIM-cov10. IBD and IBS were undertaken to further confirm IM/CIM QTL. Significance values and percentage variation explained for all QTL reported in the manuscript are derived from IM. Flanking markers were defined by CIM-cov10 or CIM-cov5 when QTL were detected by both IM and CIM; otherwise, intervals were defined by IM. QTL detected in IM analysis were named following the recommendations for gene symbolization in wheat (McIntosh et al. 2013).

### Bioinformatics, DNA sequencing, and haplotype analysis

The position of SNP markers on the wheat reference genome RefSeq v1.0 (IWGSC 2018) was obtained from the T3 wheat website (https://triticeaetoolbox.org/wheat/). Where not available, or where the retrieved chromosome assignation differed to that of the BMWpop genetic map (Stadlmeier et al. 2018), the SNP DNA flanking sequenced was used as a query for BLASTn (Altschul et al. 1990) searches of the RefSeq v1.0 wheat genome assembly using the BLAST function at EnsemblPlants release 49 (https://plants.ensembl.org/; Howe et al. 2019) using the default search parameters, and the position of the SNP on the correct chromosome determined manually. To determine whether QTL mapped to regions of low genetic recombination, for each chromosome, marker genetic map locations (Stadlmeier et al. 2018) were plotted against their RefSeq v1.0 physical map positions, where available, using Microsoft Excel. *WAPO-A1* and *WAPO-B1* genomic sequence for the BMWpop founders was generated by direct Sanger sequencing of PCR products using the homoeologue-specific primers listed in Supplementary Table 2, following the protocols described by Cockram et al. (2007). The sequences are available in GenBank under accession numbers MW366865 to MW366879. *WAPO-A1* and *WAPO-B1* sequences for an additional 16 hexaploid wheat lines were extracted from published genome assemblies (IWGSC 2018; Walkowiak et al. 2020). DNA and protein alignments were undertaken using Clustal Omega (Madeira et al. 2019) and visualized using GeneDoc v2.7 (Nicholas et al. 1997). *WAPO* haplotypes were determined manually using sequence alignments, with the positions of DNA variants listed relative to start codon in the relevant cv. Chinese Spring gene model. Protein domains were identified using Pfam 33.1 (El-Gebali et al. 2019). Visualization of amino acid sequence conservation at specific regions was undertaken in WebLogo (Crooks et al. 2004) using the predicted proteins of plant species with sequenced genomes identified as ‘orthologous’ in Ensembl Plants release 49, and for which amino acid sequence similarity with WAPO-A1 or WAPO-B1 was > 20% (listed in Supplementary Table 3). For the traits that co-located at the chromosome 7A locus controlling spikelet number phenotypes, haplotype analysis was conducted using the meta-analysis BLUEs, from which means for each trait were calculated and the RILs grouped according to marker *wsnp_JD_c20555_18262317* as a surrogate for *WAPO-A1* haplotype. Haplotype analysis for the 7B loci controlling spikelet number traits spanning *WAPO-B1* were conducted using the peak genetic marker identified in the meta-analysis for both ‘total number of spikelets’ and ‘number of fertile spikelets’ (*AX.94949800*) and the two flanking markers immediately flanking this SNP in the BMWpop genetic map (*Tdurum_contig81911_179* and *Kukri_c12901_706*). To account for the strong effect of the 7A locus on spikelet number traits, we used SNP *wsnp_JD_c20555_18262317* as a co-factor to generate the BLUEs used for the haplotype analysis of the 7B QTL. The significance of the difference between the means for each haplotype at the 7A or 7B locus was determined by Wilcoxon test at *p* < 0.05 using the R/ggpubr package (Kassambara 2020). Gene expression data was sourced from Li et al. (2018b) and Ramírez-González et al. (2018) following the methods outlined in Supplementary Text 1, and heatmaps generated using the heatmap.2 function in the gplots package (Warnes et al. 2012) in R.

## Results

### Phenotyping, transgressive segregation, and heritabilities

Three replicated field trials were undertaken using the BMWpop: two in the UK in 2017 and 2018 (UK17, UK18) and one in Germany in 2018 (DE18). Across all 394 RILs and eight founders, 15 phenotypes were collected (listed in Table 1): 13 yield components, as well as ‘plant height’ and ‘flowering time’. BLUEs were calculated for all traits and are listed in Supplementary Table 4. Phenotypic data for all traits were found to be normally distributed, and so did not require further transformation. Transgressive segregation was observed in both the positive and negative direction for all traits in all 45 year/location combinations analyzed (Supplementary Fig. 1). This was especially notable for ‘number of infertile spikelets per ear’, ‘total number of spikelets per ear’, ‘seed length’ and ‘flowering time’. Heritabilities varied depending on phenotype and year (Table 1), ranging from 0.42 (‘seed weight/ear weight ratio’ in trial UK17) to 0.95 (‘seed length–width ratio’ in DE18). Mean heritability for seed traits (SA, SWI, SL, FFD, SL.SWI, TGW; mean *h*^2^ = 0.80) were higher than those for ear architecture (EL, EW, NFSP, NISP, totNSP, NS.NE, SW.EW; mean *h*^2^ = 0.71). Individual trait heritabilities were broadly similar between the three trials, with 12 of the 15 traits showing differences in their heritabilities lower than 0.26. The exceptions were ‘seed weight-ear weight ratio’, ‘number of seeds per ear’, and ‘thousand grain weight’, with the former driven by low heritability in UK17 (0.42), and the latter two by relatively low heritability in UK18 (0.46 and 0.61, respectively).

**Table 1:** Trait descriptions, trait heritabilities (*h*^*2*^), and trait correlations between trials. Listed are the 15 traits measured in the BMWpop over three trials, conducted in the United Kingdom in 2017 (UK17) and 2018 (UK18), and in Germany in 2018 (DE18)

Trait code	Trait name	UK17 *h*^*2*^	UK18 *h*^*2*^	DE18 *h*^*2*^	Max. *h*^*2*^ difference	Corr. UK17 vs. UK18	Corr. UK17 vs. DE18	Corr. UK18 vs. DE18
EL	Ear length (cm)	0.77	0.75	0.86	0.11	0.71	0.74	0.80
EW	Ear weight (g)	0.60	0.69	0.80	0.20	0.65	0.29	0.22
NFSP	No. fertile spikelets per ear	0.77	0.66	0.85	0.19	0.71	0.74	0.70
NISP	No. infertile spikelets per ear	0.57	0.57	0.78	0.21	0.35	0.43	0.14
totNSP	Total no. spikelets per ear	0.85	0.70	0.81	0.15	0.76	0.83	0.76
NS.NE	Number of seeds per ear	0.65	0.46	0.85	0.39	0.42	0.45	0.26
WS.EW	Seed weight/ear weight ratio	0.42	0.84	0.62	0.42	0.11	0.16	0.04
SA	Seed area (mm^2^)	0.77	0.85	0.91	0.14	0.67	0.53	0.47
SWI	Seed width (mm)	0.71	0.83	0.84	0.13	0.6	0.45	0.36
SL	Seed length (mm)	0.84	0.89	0.69	0.20	0.73	0.60	0.49
FFD	Factor form density	0.72	0.77	0.75	0.05	0.61	0.40	0.33
SL.SWI	Seed length/seed width ratio	0.78	0.89	0.95	0.17	0.66	0.66	0.53
FT	Flowering time (days)	0.82	0.63	0.88	0.25	0.73	0.76	0.74
HT	Plant height (cm)	0.83	0.77	0.93	0.16	0.68	0.60	0.74
TGW	Thousand grain weight (g)	0.74	0.61	0.93	0.32	0.66	0.49	0.46
*Mean*		0.72	0.73	0.83	0.21	0.60	0.54	0.47

All-by-all trait correlations are listed in Supplementary Table 5

### Trait correlations

**Fig. 1:**
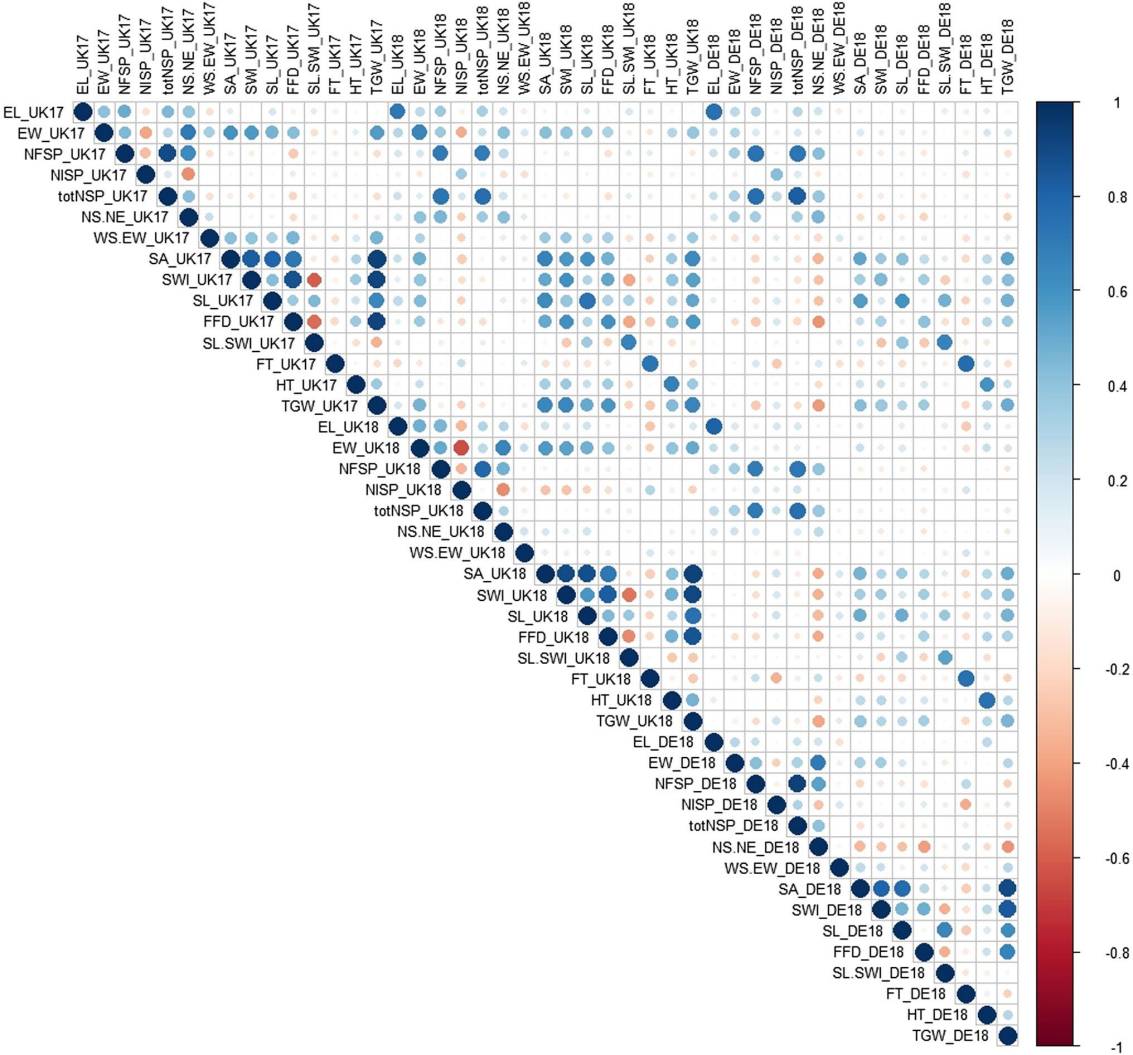
Correlations between the 15 traits measured in the BMWpop grown in field trials undertaken in the United Kingdom in 2017 and 2018 (UK17 and UK 18) and in Germany in 2018 (DE18). Trait abbreviations: EL (ear length), EW (ear width), NFSP (number of fertile spikelets per ear), NISP (number of infertile spikelets per ear), totNSP (total number of spikelets per ear), NS.NE (number of seeds per ear), WS.EW (seed weight/ear weight ratio), SA (seed area), SWI (seed width), SL (seed length), FFD (factor form density), SL.SWI (seed length/seed width ratio), FT (flowering time), HT (plant height)

Correlations between all trait and year combinations are illustrated in Fig. 1, with the underlying values listed in Supplementary Table 5. Correlation within a trait between years was generally high (median = 0.57), with 35 of the 45 between year comparisons (78%) showing a correlation ≥ 0.4 (see also Table 1). As might be expected, the strength of inter-year correlations was itself correlated with trait heritabilities, such that traits with low heritability also tended to have low correlations between years. Overall, traits measured in the two UK trials showed the highest between year correlations (mean = 0.60), followed by trial UK17 versus DE18 and UK18 versus DE18 (*r* = 0.55 and 0.47, respectively), possibly reflecting the contrasting agricultural environments between the sites in the UK and Germany. Particularly strong positive correlations across all three trials were observed for the yield components ‘ear length’ (*r* ≥ 0.71), ‘number of fertile spikelets per ear’ (*r* ≥ 0.70), and ‘total number of fertile spikelets per ear’ (*r* ≥ 0.76), and seed morphometric traits generally showed good positive inter-year correlations, ranging from 0.36 (‘seed width’, UK18 versus DE18) to 0.73 (‘seed length’, UK17 versus UK18). In addition to yield component traits, ‘flowering time’ and ‘plant height’ both showed high inter-year correlations with values above 0.7 and 0.6, respectively. Notable exceptions to these high correlation trends were the ear traits ‘number of infertile spikelets per ear’ (*r* = 0.14 to 0.43) and ‘seed weight/ear weight ratio’ (*r* = 0.04 to 0.16), and to a lesser extent ‘ear weight’ (*r* = 0.22 to 0.65), for which low correlations were driven by comparisons of the UK versus German trials (Table 1).

Considering correlations between traits within a given trial, the commercially important agronomic trait ‘thousand grain weight’ was negatively correlated, albeit relatively weakly in some cases, with the ‘number of fertile spikelets’, ‘total number of spikelets’ and the ‘number of seeds per ear’. This indicated increasing the fertility within the ear comes at the cost of reducing the average size of a given grain amongst the range of grain sizes achieved within a given ear. Notably high positive correlations within each of the three trials were found between ‘thousand grain weight’ and the grain morphometric traits ‘seed area’, ‘seed width’, ‘seed length’ and ‘factor form density’ (Fig. 1, Supplementary Table 5). Furthermore, these traits also showed good positive correlation within all trials with the ear-related traits ‘ear weight’ and ‘ear length’. Conversely, relatively strong negative correlations within each year were observed for the two UK trials between the ‘number of infertile spikelets’ and the four ear traits (‘ear weight’, ‘ear length’, ‘number of seed per ear’ and ‘number of fertile spikelets’), particularly in UK18, which would make sense based on prior assumptions. In the German trial, however, ‘number of infertile spikelets per ear’ was positively correlated with the ‘total number of spikelets per ear’ (*r* = 0.30, *p* = 4.91^–8^), indicating different yield component trade-offs may have taken place in the German growing environment. Indeed, ‘number of infertile spikelets’ was relatively strongly negatively correlated with ‘flowering time’ in DE18 (*r* = -0.37, *p* = 1.68^–14^), while this was not notably the case in the two UK trials. Collectively, these observations indicate different factors between the German trial and those in the UK played a part in driving the ‘number of sterile spikelets per ear’, as well as the trade-off between the ‘number of seeds per ear’ and seed morphometric traits. In both UK trials, ‘ear weight’ was positively correlated with ‘plant height’ and negatively correlated with ‘flowering time’, although these correlations were not seen in the German trial.

### Genetic analyses

**Fig. 2:**
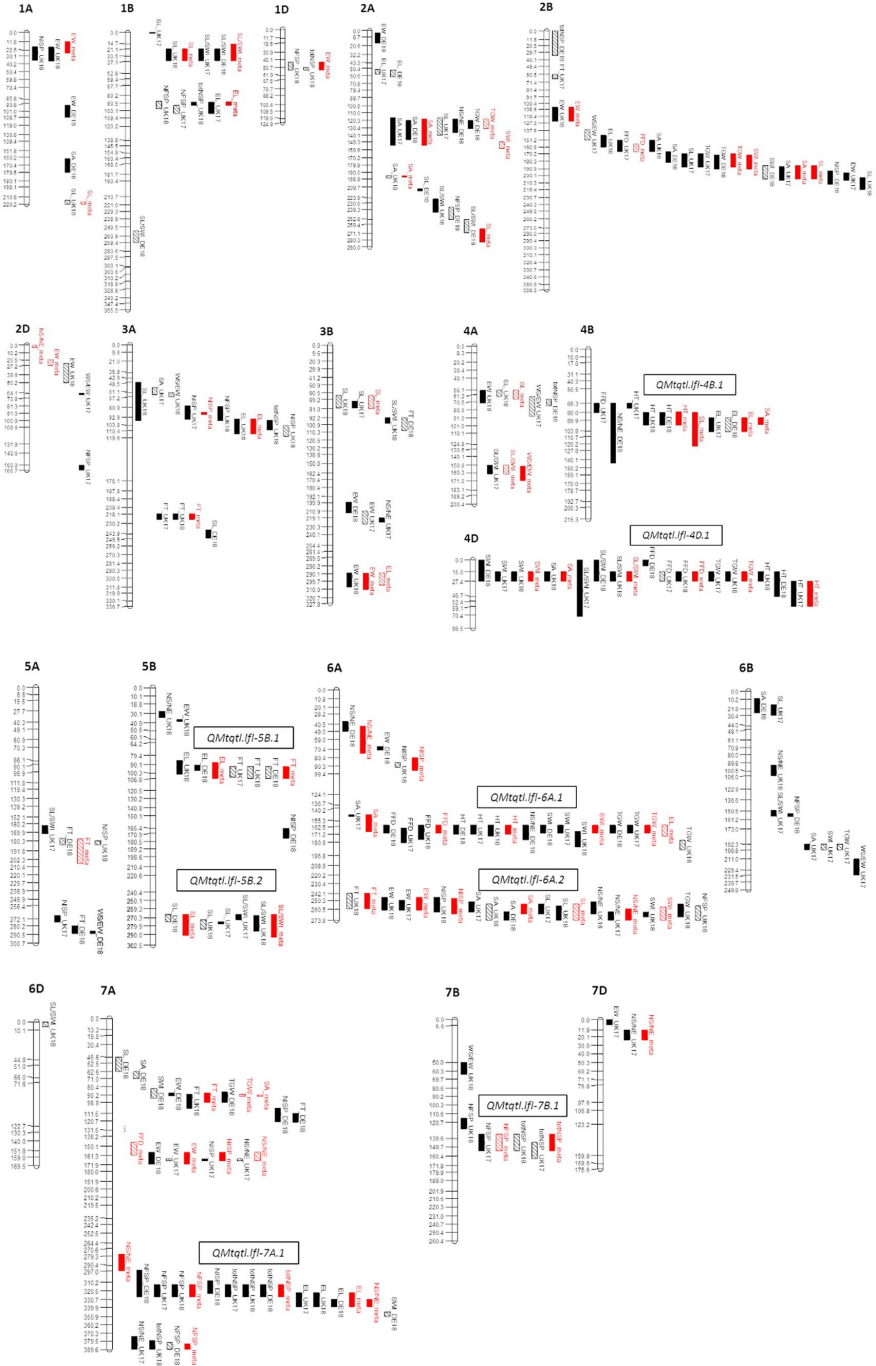
The genetic map locations of quantitative trait loci (QTL) identified in the BMWpop grown in field trials undertaken in the United Kingdom in 2017 and 2018 (UK17 and UK 18) and in Germany in 2018 (DE18) using interval mapping and composite interval mapping. Multi-trait QTL (MT-QTL) are also included, and are shown in red. ‘Strong’ QTL, classified as those with -log10*p-*values higher than the trait-specific *p* = *0.05* significance thresholds determined by permutation are indicated by solid bars. ‘Weak’ QTL, classified as those with -log_10_*p-*values less than the permutated *p* = 0.05 significance threshold but with -log_10_*p* > 3 and which explain ≥ 5% of the phenotypic variation, are indicated by bars with diagonal lines. The BMWpop genetic map is previously published (Stadlmeier et al. 2018)

Across all three trials, 187 individual QTL (excluding QTL from the meta-analysis) were detected across the 15 traits investigated: 63 in UK17, 65 in UK18 and 59 in DE18 (Fig. 2; Supplementary Table 6). These QTL were distributed across 19 of the 21 chromosomes of wheat, with fewer located on the wheat D sub-genome (23) than on the A and B sub-genomes (97 and 67, respectively). Within each trial, the average number of QTL per trait was four. The numbers of QTL per trait ranged from two (for ‘total number of spikelets’) to seven (‘seed length’) in UK17, from two (‘factor form density’ and ‘weight of seed as a proportion of ear weight’) to eight (‘seed length’) in UK18, and from one (‘weight of seed as a proportion of ear weight’) to six (‘ear weight’ and ‘seed area’) in DE18. Of the 187 individual QTL, 45% were found to be replicated for a given trait in two or more trials, resulting in the identification of 34 genetic loci controlling 13 of the 15 traits investigated (Fig. 2; Supplementary Table 6). The number of replicated QTL per trait ranged from one (for ‘number of seeds per ear’) to four (‘ear length’). No replicated QTL were identified for ‘number of infertile spikelets’ and ‘seed weight-ear weight ratio’.

### QTL controlling multiple traits

Of the 34 replicated QTL, 24 were found to resolve into eight genetic loci each controlling ≥ 2 traits, located on chromosomes 4B, 4D, 5B, 6A, 7A and 7B. Here, these are termed ‘multi-trait QTL’ (MT-QTL) and named as *QMtqtl.lfl-XX,* where ‘*XX*’ indicates chromosome (Fig. 2; Supplementary Table 6). All three of the replicated QTL for ‘plant height ‘identified in each of our three trials were found to co-locate with MT-QTL (*QMtqtl.lfl-4B.1, QMtqtl.lfl-4D.1* and *QMtqtl.lfl-6A.1*). The most significant height QTL identified correspond to the homoeologous *Reduced height* (*Rht*) loci *Rht-B1* and *Rht*-*D1*, explaining 5.7–9.4 and 14.5–29.9% of the phenotypic variation for plant height, respectively. These loci have been map-based cloned, and control the semi-dwarfing phenotype of modern wheat that played a central role in the ‘Green Revolution’ (Peng et al. 1999), and both have previously been shown to segregate in the BMWpop (Stadlmeier et al. 2019) (for BMWpop founder alleles at these loci, see Supplementary Table 7). At *Rht-D1*, replicated QTL for five traits were identified (‘plant height’, ‘seed width’, ‘seed length–width ratio’, ‘factor form density and ‘thousand grain weight’), while two replicated QTL co-segregated at *Rht-B1* (‘plant height’ and ‘ear length’). At each of these two loci, the direction of the predicted allelic haplotype effects for each of the eight founders for ‘plant height’ were found to be similar to those of the corresponding yield component QTL, indicating pleiotropy rather than genetic linkage was more likely the cause for QTL co-localization (Supplementary Table 6). The third ‘plant height’ QTL, located on chromosome 6A, controlled 9–12% of the phenotypic variation and co-located with QTL for three grain-related traits (‘seed width’, ‘factor form density’ and ‘thousand grain weight’). The genetic locus *Rht24* controlling plant height is reported to be located on chromosome 6A (Tian et al. 2017; Würschum et al. 2017; Herter et al. 2018; Scott et al. 2020b). Anchoring the flanking markers of our chromosome 6A ‘plant height’ QTL to the wheat reference genome (6A: 409–520 Mb, QTL peak at 439 Mb; Supplementary Fig. 2; Supplementary Table 6) found it to span the *Rht24* interval as defined by Würschum et al. (2017) (6A: 400–450 Mb). We therefore subsequently refer to this locus in the BMWpop as *Rht24*. Predicted allelic effects indicated the founders Ambition and Potenzial most consistently conferred a reduction in height, and that the allelic effects for these two founders conferred the strongest reduction at the co-locating QTL for ‘seed width’, ‘factor form density’ and ‘thousand grain weight’, thus indicating height affected these seed traits pleiotropically (Supplementary Table 6). A second MT-QTL was also located on chromosome 6A, *QMtqtl.lfl-6A.2*, consisting of replicated QTL for ‘ear weight’, ‘number of seed per ear’, ‘seed length’ and ‘seed area’ (Fig. 2, Supplementary Table 6). Predicted founder effects indicated that the founder Format generally had the strongest and most consistent effect on reducing trait expression for all four co-locating traits.

Two MT-QTL were located on chromosome 5B, neither of which were located within the region of low genetic recombination spanning the centromere (Fig. 2, Supplementary Table 6). *QMtqtl.lfl-5B.1* consisted of replicated QTL for ‘flowering time’ and ‘ear length’, explaining 5.0–5.9 and 6.5–10.0% of the phenotypic variation, respectively. The trends in the predicted allelic effects at this locus found founders Firl3565, Potenzial and Bussard to most strongly increase ‘ear length’. While alleles at these three founders also resulted in earlier ‘flowering time’, the effect was not always consistent, and so is not clear whether pleiotropic effects of flowering on ear length occur at this locus. At *QMtqtl.lfl-5B.2*, QTL for two grain morphometric traits colocalized, ‘seed length’ and ‘seed length-seed width ratio’, controlling 5.0–7.1% and 7.3–7.9% of the phenotypic variation, respectively. Predicted allelic effects from the founders Event and Format consistently resulted in increased values for both traits, indicating pleiotropy, as might be expected given that ‘seed length-seed width ratio’ is partially derived from measured values for ‘seed length’.

**Fig. 3:**
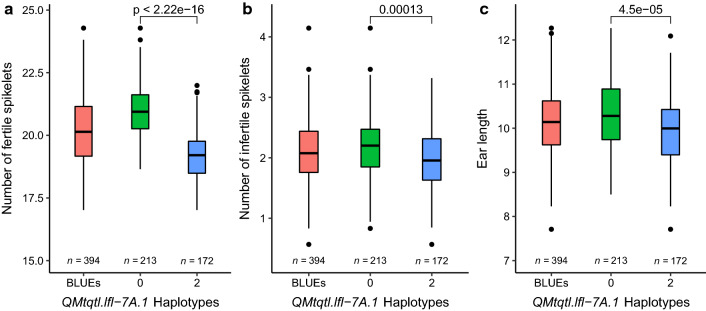
Analysis of the effects of haplotypes *WAPO-A1.hap1 WAPO-A1.hap2* at the multi-trait QTL *QMtqtl.lfl-7A.1*controlling spikelet number traits on chromosome 7A. Allelic state at SNP *wsnp_JD_c20555_18262317*, located six gene models from *WAPO-A1* in the wheat reference genome (RefSeq v1.0, gene model build RefSeq v1.2; IWGSC 2018), was found to be in phase with the two *WAPO-A1* haplotypes present in the eight BMWpop founders. This SNP was therefore used group the BMWpop recombinant inbred lines (RILs) for each of the three traits for which QTL co-located at *QMtqtl.lfl-7A.1*: (**a**) ‘number of fertile spikelets’, (**b**) ‘number of infertile spikelets’, and (**c**) ‘ear length’. For each of these traits, the meta-analysis best linear unbiased estimates (BLUEs) were used for haplotype analysis. The trait values for all 394 BMWpop RILs are shown on the left in red. The significance of the differences between haplotypes was determined by Wilcoxon test (*p* < 0.05)

Finally, MT-QTL *QMtqtl.lfl-7A.1* and *QMtqtl.lfl-7B.1* were identified on homoeologous positions on the long arm of chromosomes 7A and 7B, respectively (Fig. 2; Supplementary Table 6). *QMtqtl.lfl-7A.1* encompassed three ear related traits (‘number of fertile spikelets’, ‘total number of spikelets’ and ‘ear length’), with allelic effects from founders 1, 5, 6 and 7 (Event, Format, Potenzial and Bussard) predicted to increase the numbers of both fertile and infertile spikelets at all sites and years. However, the observed trends for the predicted allelic effects at the ‘ear length’ QTL differed, with increasing alleles conferred by founders 1, 2, 4, 6 and 7 (Event, BAYP4535, Firl3565, Potenzial and Bussard), indicating the possible presence of a linked QTL controlling ‘ear length’. The physical position of our chromosome 7A ‘number of fertile spikelets’ QTL (peak marker *wsnp_JD_c20555_18262317* at 674.277 Mb) corresponds to the strong effect spikelet number QTL identified in other studies (e.g. 673.779 to 674.277 Mb, Kuzay et al. 2019; 673.780 to 674.300 Mb, Muqaddasi et al. 2019) and was located eight gene models from the wheat gene *WAPO-A1* (*TraesCS7A02G481600*, located at 674.081 Mbp; Supplementary Fig. 3) previously identified as a likely candidate underlying this chromosome 7A QTL (Kuzay et al. 2019; Muqaddasi et al. 2019). We extracted *WAPO-A1* DNA sequences from 16 recently sequenced genome assemblies (Walkowiak et al. 2020), including 1000 bp up- and down-stream of the coding regions, identifying 21 DNA variants defining four haplotypes (Supplementary Figs. 3–4; Supplementary Table 8a). Sequencing a 1,719 bp region spanning the *WAPO-A1* coding regions in the eight BMWpop founders (as well as 12 bp and 251 bp up- and down-stream of the start and stop codon, respectively), found the parental lines to possess two of the four haplotypes identified in the 16 lines with sequenced genomes (based on the common 1,719 bp region): *WAPO-A1.hap1* was carried by the four BMWpop founders with low spikelet number alleles at *QMtqtl-7A.1* (Ambition, BAYP4535, Firl3565 and Julius), while *WAPO-A1.hap2* was present in the four founders with high spikelet number alleles (Bussard, Event, Format and Potenzial) (Supplementary Figs. 3–4; Supplementary Table 8a). *WAPO-A1.hap2* included two SNPs that resulted in amino acid substitutions relative to *WAPO-A1.hap1*: a cysteine to phenylalanine at amino acid position 47 (C47/F) located within a highly conserved position in the F-box domain, and an asparagine to aspartate substitution at position 384 (N384/D) at a non-conserved residue toward the C-terminus (Supplementary Figs. 3–4; Supplementary Table 8a), as reported previously by Kuzay et al. (2019). The peak SNP identified at the BMWpop chromosome 7A spikelet number locus (*wsnp_JD_c20555_18262317*) was located seven gene models away from *WAPO-A1*, with the two alleles at SNP *wsnp_JD_c20555_18262317* distinguishing the two *WAPO-A1* haplotypes present in the BMWpop founders. Grouping the BMWpop RILs according to allele call at *wsnp_JD_c20555_18262317* found the RILs predicted to carry *WAPO-A1.hap2* to have a significant increase in the number of fertile spikelets (*P* < 2.22e^−16^), the number of infertile spikelets (*P* = 0.00013) and ear length (*P* = 4.5e^−05^), compared to those predicted to carry haplotype *WAPO-A1.hap1* (Fig. 3).

**Fig. 4:**
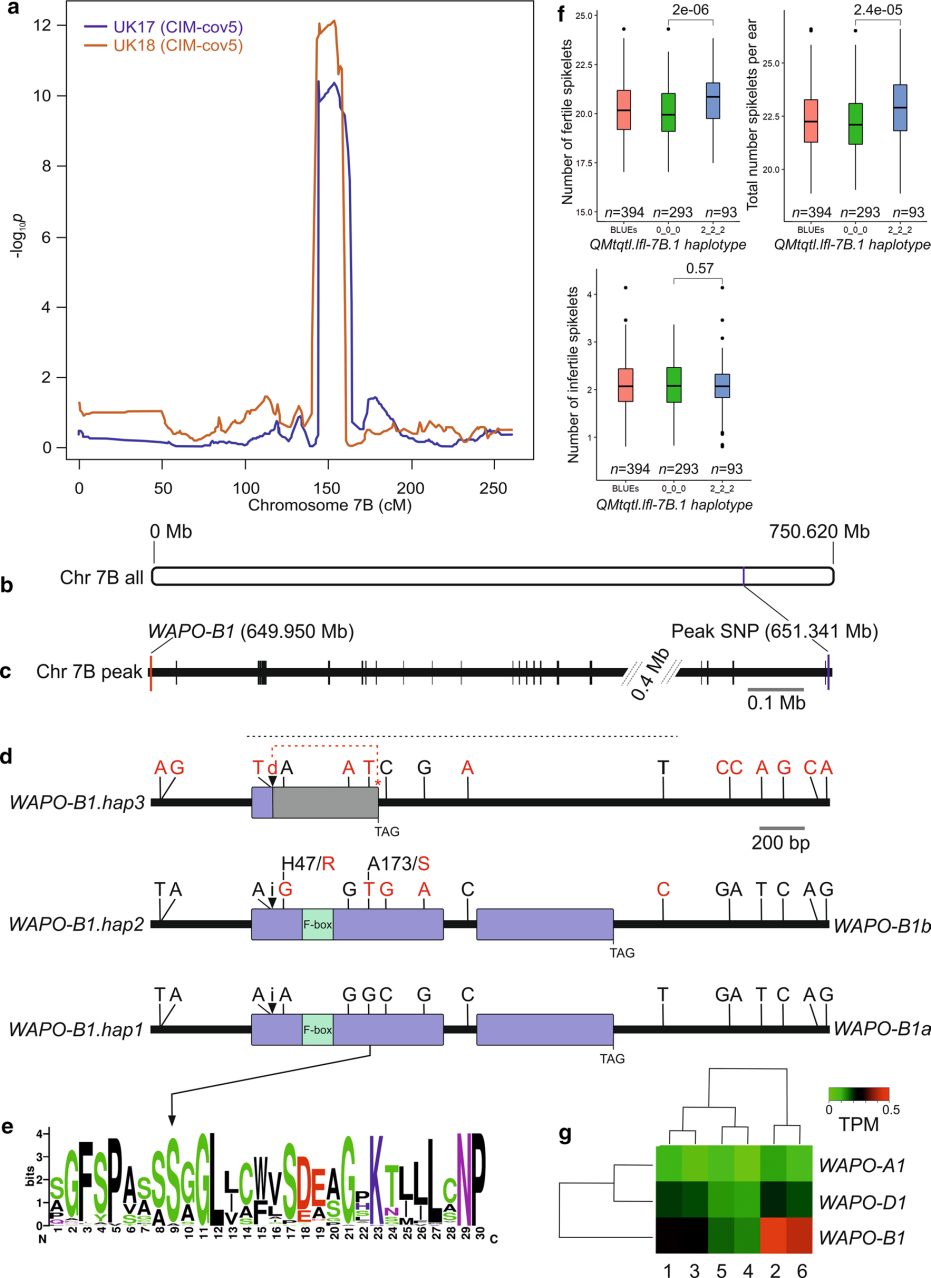
The BMWpop multi-trait QTL *QMtqtl.lfl-7B.1* and details of the underlying candidate gene, *WAPO-B1*. (**a**) QTL on chromosome 7B identified in trials conducted in the United Kingdom in 2017 (UK17) and 2018 (UK18) for the trait ‘total number of spikelets’, one of the three traits with co-locating QTL at *QMtqtl.lfl-7B.1*. Results of composite interval mapping with five covariates are shown (note, while the significance of the QTL is inflated relative to the results of interval mapping without the inclusion of covariates shown in Supplementary Table 6, the definition of the QTL intervals is improved and so is used here). (**b**) Location on the chromosome 7B physical map (RefSeq v1.0; IWGSC 2018) of the peak SNP marker (*AX.94949800*, in gene model *TraesCS7B02G386400*) identified at QTL for ‘total number of spikelets’ in trials UK17 and UK18. (**c**) the location *WAPO-B1* (*TraesCS7B02G384000*) on the physical map, 24 high-confidence gene models from the peak SNP identified by QTL mapping. (**d**) *WAPO-B1* haplotypes, based on the DNA variants identified by comparing sequences from the wheat reference genome assembly (IWGSC 2018) with those from 15 additional hexaploid wheat lines with sequenced genomes (Walkowiak et al. 2020). The positions of the 17 DNA variants that define the haplotypes are as detailed in Supplementary Table 8b, with changes in the DNA and amino acid sequences from that of the reference sequence of cv. Chinese Spring indicated in red. The 5 bp insertion (1) / deletion (**d**) in exon-1 present in *WAPO-B1.hap3* is indicated, and leads to a frame shift (grey) that spans the F-box domain and a subsequent premature stop codon (red asterisk). Sequencing the 1,893 bp region indicated by the dashed horizontal black line in seven of the eight BMWpop founders allowed allocation as *WAPO-B1.hap1* (Ambition, Bussard, Event, Format and BAY4535, all of which carried the low spikelet number allele, designated here *WAPO-B1a*) or *WAPO-B1.hap2* (Julius and Potenzial, allele *WAPO-B1b*); *WAPO-B1* haplotype in the founder Firl3565 is currently undetermined as we were unable to amplify the gene. Of the five SNPs that differentiated the two *WAPO-B1* haplotypes in the seven BMWpop founders, two lead to alterations of the predicted protein in *WAPO-B1.hap2*. The first was an A + 140/G SNP that results in a H/47R amino acid substitution in a highly non-conserved region of the protein (alignment shown in Supplementary Fig. 6). The second was a G + 517/T SNP resulting in an A173/S amino acid substitution, (**e**) located in a highly conserved region of the protein, based on alignment of 56 proteins from 53 plant species. (**f**) Haplotype analysis at the *QMtqtl.lfl-7B.1* locus in the BMWpop recombinant inbred lines (RILs). Haplotypes were constructed using three SNPs: *Tdurum_contig81911_179*, *Kukri_c12901_706* and *AX.94949800* (the peak marker from the meta-analyses for ‘number of fertile spikelets’ and ‘total number of spikelets’), located at RefSeq v1.0 positions 646,037,877, 646,063,954 and 651,340,580 bp, respectively. Haplotype `000’ (representing the combination of the allele calls at each of the three SNPs) tags the low spikelet *WAPO-B1a* allele (Ambition, Bussard, Event, Format, BAYP4535) while haplotype `222′ tags the high spikelet *WAPO-B1b* allele (Julius and Potenzial, as well as Firl3565). All BMWpop RILs carried haplotypes `000′ or `222′, except for two lines carrying haplotype ‘002′ which were excluded for the purposes of the analysis presented here. (**g**) *WAPO-A1, -B1* and *-D1* gene expression in the apical meristem at different stages of development, measured as transcripts per million reads (TPM), sourced from Li et al. (2018b). Apical meristem developmental stages included are numbered in developmental order: 1 = vegetative, 2 = elongation, 3 = single ridge, 4 = double ridge, 5 = glume primordium, 6 = floret differentiation. At stage 4, the meristem has transitioned from the vegetative to reproductive stage

Multi-trait QTL *QMtqtl.lfl-7B.1*, located on chromosome 7B at a homoeologous location to the 7A QTL *QMtqtl.lfl-7A.1*, included co-locating QTL for two related spikelet fertility traits (‘number of fertile spikelets’ and ‘total number of spikelets’) that were identified in both UK trials and the meta-analysis (Fig. 4a; Supplementary Table 6). Predicted allelic effects were relatively variable within and between these trait/year combinations, although the effects were more consistent in the meta-analysis with the founders Firl3565, Potenzial and Julius carrying alleles for higher spikelet number (Supplementary Table 6). Analysis of the IBS results confirmed the IM/CIM result, with significant markers identified for the 7B locus in trials UK17 and UK18 for ‘number of fertile spikelets’ (*p*_adj_ ≤ 0.0008) and ‘total number of spikelets’ (*p*_*adj*_ ≤ 0.0010) (Supplementary Table 6). Although genetic analysis using IM/CIM did not identify a significant spikelet number related QTL at the chromosome 7B locus in the DE18 trial, IBS genetic analysis of DE18 identified significant hits for both ‘number of fertile spikelets’ (*p*_adj_ = 0.0060) and ‘total number of spikelets (*p*_adj_ = 0.0039). *WAPO-B1* (*TraesCS7B02G384000*) is located on chromosome 7B at 649.950 Mb, 24 gene models away from the peak markers identified in the IM/CIM meta-analyses for the two spikelet traits (*AX.94949800*, chromosome 7B at 651.340 Mb) (Fig. 4b–c). As the chromosome 7A and 7B spikelet number QTL identified in the BMWpop were located at homoeologous positions, we first investigated the *WAPO-B1* haplotypes present in the 16 hexaploid wheat lines with sequenced genome assemblies used in our analysis of *WAPO-A1*. Extracting *WAPO-B1* genomic sequences, including 1 kb up- and down-stream of the gene, identified regions of missing sequence around a (CT)_n_ microsatellite upstream of the start codon (Supplementary Fig. 5). We therefore considered the regions starting immediately downstream of this microsatellite in the extracted sequences, identifying 17 polymorphic sites that defined three haplotypes: *WAPO-B1.hap1, WAPO-B1.hap2* and *WAPO-B1.hap3* (Fig. 4d; Supplementary Fig. 5; Supplementary Table 8b). Sequencing a 1893 bp region spanning *WAPO-B1* in the eight BMWpop founders, and aligning these to the genomic *WAPO-B1* sequences extracted from the 16 lines with genome assemblies, found two of the three *WAPO-B1* haplotypes to be present in the BMWpop. The BMWpop founders carrying the low spikelet number allele at *QMtqtl.lfl-7B.1* all possessed haplotype *WAPO-B1.hap1* (Ambition, Bussard, Event, Format and BAYP4535), while two of the three founders with the high spikelet number allele at the locus carried *WAPO-B1.hap2* (Fig. 4d; Supplementary Table 8b). These two *WAPO-B1* haplotypes were differed to each other by five SNPs: four in exon-1 and one in the 3′ untranslated region (UTR). Of the four exonic SNPs, two did not result in an amino acid change in the predicted protein (G + 594/C and A + 762/G), while the remaining two SNPs resulted in non-synonymous substitutions. The first, G + 140/A, resulted in an arginine to histidine substitution at amino acid residue 47 (R47/H), with alignment of WAPO proteins from 53 plant species showing this substitution to be located in a region of very low sequence alignment close to the N-terminus (Supplementary Fig. 6). The second non-synonymous SNP (T + 517/G) resulted in a serine to alanine substitution at residue 173 (S173/A, BLOSUM62 score = 1, indicating a disruptive amino acid change) within a highly conserved region of the *WAPO-B1* predicted protein at which the S residue was present in 56 of the 53 species investigated (Fig. 4e). We could not amplify *WAPO-B1* in the BMWpop founder Firl3565, despite trying three primer combinations targeting different regions of the gene (Supplementary Table 2). To investigate haplotype effects at *QMtqtl.lfl-7B.1* on spikelet traits in the BMWpop RILs, we constructed a three-SNP haplotype at the QTL using the most significant marker identified in the meta-analysis (*AX.94949800*) and the two flanking SNPs in the genetic map (*Tdurum_contig81911_179* and *Kukri_c12901_706*). The low (designated here *WAPO-B1a*) and high (*WAPO-B1b*) spikelet number alleles identified in the founders were represented by the three-SNP haplotypes ‘000′ and ‘222′, respectively. After using the chromosome 7A SNP *wsnp_JD_c20555_18262317* at *QMtqtl.lfl-7A.1* as a cofactor to account for the major effect of this locus on spikelet traits, haplotype analysis of the resulting BLUEs found significant differences between the three-SNP haplotypes at *QMtqtl-lfl-7B.1* for the traits ‘number of fertile spikelets’ and ‘total number of spikelets’, but not for ‘number of infertile spikelets’ (Fig. 4f). Finally, in silico analysis of gene expression found *WAPO-B1* to be expressed in similar tissues to its previously characterized homoeologue *WAPO-A1*, including the shoot apical meristem at different growth stages, with particular high expression at the meristem elongation stage and floret differentiation stage (Fig. 4g; Supplementary Fig. 7). Unlike *WAPO-A1* however, *WAPO-D1* also showed notably high expression in grain tissues.

### Additional replicated QTL

Nine replicated QTL were identified outside of the regions designated as MT-QTL (Supplementary Table 6). The most significant of these were: (a) the ‘seed length/width ratio’ QTL *QSlsw.lfl-1B.1* (IM-cov0 -log10*p* = 5.9–8.0), which explained 7.8–8.5% of the phenotypic variation, and for which the allele from the founder Event was predicted to increase trait expression, and (b) the ‘flowering time’ QTL *QFt.lfl-3A.1* (IM-cov0 -log10*p* = 5.1–8.0), explaining 6.8–10.0% of the phenotypic variation and for which alleles from the founders Firl3565 and Format were predicted to delay flowering. Two QTL were identified for ‘seed area’ at approximately colinear positions spanning the regions of very low genetic recombination that encompass the centromeres on chromosomes 2A (*QSa.lfl-2A.1*) and 2B (*QSa.lfl-2B.1*) (Fig. 2; Supplementary Table 6), explaining around 6% and 5% of the phenotypic variation, respectively. Also located on chromosome 2B was a replicated QTL for ‘thousand grain weight’ (*QTgw.lfl-2B.1*) explaining 5.9–8.5% of the variation. This QTL also spanned the highly non-recombining region across the chromosome 2B centromere, as did the chromosome 2B ‘seed area’ QTL (Supplementary Table 6). However, as this region is predicted to contain around half of the physical region of chromosome 2B, we did not classify these QTL as MT-QTL, despite their genetic map locations overlapping to some degree in some year/trial combinations. A ‘flowering time’ QTL identified in the UK trials, *QFt.lfl-3A.1*, was identified on the long arm of chromosome 3A and explained 6.8–10.0% of the variation. Finally, two replicated QTL were found on chromosome 3B. The first, *QSl.lfl-3B.1*, was located on the short arm and explained 5.2–6.4% of the variation for ‘seed length’, while the second, *QEw.lfl-3B.1*, was on the long arm and explained ~ 6% of the variation for ‘ear weight’.

## Discussion

### Trade-offs between key yield component traits and their interaction with plan height

Analysis of the correlations between yield component traits can give insight into the trade-offs that take place between them. Grain number per ear is one of the key components determining final grain yield and is largely set during the early stages of reproductive development (Sreenivasulu and Schnurbusch 2012). Our finding that ‘total number of seeds per ear’ was negatively associated with seed morphometric traits has been documented by others (e.g. Slafer and Miralles 1993; Sadras 2007; Bustos et al. 2013). We interpret this as a source limitation on grain yield/size, i.e. in lines where more grains are present per ear, grain filling is limited; possibly due to a lack of sufficient photo-assimilates, and/or to increased fertility in distal florets within spikelets leading to a higher number of smaller grains per ear. Our observation that the five seed morphometric traits were relatively highly positively correlated across all three trials, despite having been undertaken in different countries and/or seasons, indicates that genetic improvement specifically targeting these yield components should be robust across broad geographic areas. Indeed, the heritability of these traits was high across all trials (0.69 to 0.91). Therefore, understanding the potential trade-offs between these seed traits and other yield components could be interesting to investigate further. The finding that four seed morphometric traits (‘seed length’, ‘seed width’, ‘seed area’ and ‘factor form density’) were positively correlated with plant height across all years and locations indicated that, under our experimental conditions, taller lines supported increased grain size parameters. Indeed, as increased ‘plant height’ was not generally associated with an increase in spikelet number or number of seed per ear, the effect of this increase in height on grain morphometric traits appears to predominantly promote increased grain size without increasing the overall ear fertility. We found *Rht24* to be the second most important of the three ‘plant height’ QTL identified after *Rht-D1*, in agreement with previous analysis in the BMWpop (Stadlmeier et al. 2019). Unlike *Rht-B1* and *Rht-D1*, *Rht24* belongs to a group of gibberellic acid (GA) sensitive height altering genetic loci, and the height reducing *Rht24b* allele has been used throughout much of Europe, without notable geographic (and therefore climatic) trend (Würschum et al. 2017). Therefore, it might be assumed that ‘tall’ alleles at this locus contribute to this increase in grain morphometric traits. In addition to the *Rht24* height locus, QTL for three other traits (‘seed width’, ‘thousand grain weight’ and ‘factor form density’) co-localized within MT-QTL *QMtqtl.lfl-6A.1*. Notably, the wheat gene *TaGW2-A1* (RefSeq v1.1 gene model *TraesCS6A02G189300*, located at 237.74 Mb), orthologous to the rice gene *GRAIN WIDTH 2* controlling grain width and weight (Song et al. 2007), is predicted to lie within the *QMtqtl.lfl-6A.1* genetic interval. In wheat, artificial mutation at *TaGW2* increases grain width (Simmonds et al. 2016; Wang et al. 2018; Zhang et al. 2018a). However, in our population, both *TaGW2-A1* and *Rht24* were located within the region of very low genetic recombination of 0.538 cM that spans the chromosome 6A centromere. As a result, it is difficult for genetic mapping approaches to achieve meaningful genetic resolution for QTL mapping to much of this region. This is exemplified by a recent study that fine-mapped QTL controlling thousand grain weight and grain number per spike to a very tight genetic interval on chromosome 6A of 0.538 cM, with natural variation in the *TaGW2-A1* promotor suggested as responsible for the phenotypic differences observed (Zhai et al. 2018). However, as the 0.538 cM interval also spanned the region of very low genetic recombination spanning the chromosome 6A centromere, this represented a physical interval encompassing around a third of the chromosome (209 Mb, from 236.980 to 445.980 Mb) predicted to contain ~ 1,700 RefSeq v1.1 gene models, and is comparable to the *QMtqtl.lfl-6A.1* physical interval we identify here. Clearly, map-based approaches using experimental populations will likely find it difficult to separate the effects of allelic variation at *TaGW2-A1* from those of the many hundreds of genes within this region of low genetic recombination on chromosome 6A—possibly also including *Rht24*, estimated as located between 400–450 Mb at the distal end of the highly non-recombining region (Würschum et al. 2017). Given the well documented pleiotropic effect of *Rht-B1* (Guan et al. 2018) and *Rht-D1* on grain characters (Zhang et al. 2013), and that *Rht24* was the second strongest ‘plant height’ QTL identified in our population, the balance of evidence suggests that the presence of co-locating grain QTL in this region of chromosome 6A are more likely to be due to pleiotropic effects of *Rht24*. This assumption is supported by previous studies showing that *Rht24* pleiotropically affects thousand grain weight (Tian et al. 2017) as well as by the observations that the only other genetic loci in our population controlling height (*Rht-B1* and *Rht-D1*) also likely have strong pleiotropic effects on seed traits (Fig. 2), and that within each year, the directions of the predicted founder allelic affects at the chromosome 6A locus were similar across all co-locating QTL (Supplementary Table 6). Of course, the recent findings that even the very well characterized wheat genes *Rht-B1* and *Rht-D1* have very closely linked genes controlling thousand grain weight (Xu et al. 2019) and spikelet architecture (Dixon et al. 2018), respectively, mean that further investigation is needed to confirm the extent of any pleiotrophic effect of *Rht24* on yield components.

### Confirmation of previously described multi-trait QTL

Identification of previously characterized QTL help confirm the utility of the BMWpop for genetic dissection of yield components. In addition to the identification of the three *Rht* loci discussed above, a major effect QTL controlling spikelet number on the long arm of chromosome 7A was identified, as reported by previous studies (e.g. Quarrie et al. 2006; Würschum et al. 2018; Zhang et al. 2018b; Kuzay et al. 2019; Muqaddasi et al. 2019; Voss-Fels et al. 2019). The recent fine-mapping of this locus by Kuzay et al. (2019), as well as via additional association mapping studies using European varieties (Muqaddasi et al. 2019; Voss-Fels et al. 2019), identified *WAPO-A1* as a strong candidate gene, with a non-synonymous G/T exon-1 SNP present in the high spikelet number allele *WAPO-A1b* leading to a C47/F amino acid substitution in the F-box domain (Kuzay et al. 2019; Muqaddasi et al. 2019). The high spikelet allele at this locus was associated with *WAPO-A1* haplotype 2 (Keyaz et al. 2019). The observation that the peak SNP for our chromosome 7A QTL was seven gene models away from *WAPO-A1*, and that the high spikelet number *WAPO-A1b* allele at this QTL was conferred by founders carrying haplotype *WAPO-A1.hap2*, further demonstrates the utility of the population for fine-mapping. This chromosome 7A yield component QTL is unusual for wheat, in that it controls a notably large proportion of the phenotypic variance, such that the resulting difference in spikelet number is readily visible by eye. Indeed, analysis across a dataset of predominantly north-western European wheat varieties has previously shown that this locus (based on the location of SNP marker *IAAV5268* at 679.839 Mb) has been under strong breeder selection over the last two decades (Fradgley et al. 2019). We identify effects at this QTL in trials located in two European countries, indicating it is relatively unaffected by prevailing local environmental conditions, as also noted by Voss-Fels et al. (2019). The reason for the strong phenotypic effect of this locus has been suggested by Kuzay et al. (2019) to be because spikelet number is laid down relatively early during spike development (Bonnet 1936), and so is less prone to environmental effects such as heat and lower water availability later in the growing season as the plants mature. Alternatively, it could be due the presence of putative functional mutations in both the promotor (variant 3 in Supplementary Table 8a) and at conserved exonic locations (variants 10 and 15) (Kuzay et al. 2019). Whatever the underlying reason, increasing spikelet number per ear does not on its own necessarily result in overall increases in grain yield, due to pleiotropic effects on other yield components. Indeed, in our MAGIC population we found thousand grain weight and all grain morphometric traits measured to be negatively correlated with increased spikelet number, albeit weakly (Fig. 1). Kuzay et al. (2019) report that the increase in spikelet number afforded by the *WAPO-A1b* allele is associated with an increase in overall grain yield in both an association mapping panel and a bi-parental population, although a decrease in grain weight was also observed in the association panel. It is possible that this contrast in the effect of increased spikelet number on grain weight may be because any panel of wheat varieties investigated has, by definition, been subjected to intense breeder selection for beneficial agronomic traits—principally grain yield. In such genetic backgrounds, the ~ 15% increase in spikelet number afforded by the *WAPO-A1b* allele may expose the source limitations of the plant, i.e. the ability to supply the developing florets and grain with sufficient resources to maximize grain fill and grain set. In a bi-parental population, such artificial selection to push yield to its limits has not been imposed, and so trade-offs with grain size may not be so apparent. Indeed, we did not find any other grain QTL to co-locate with the chromosome 7A QTL identified in the BMWpop. Having said that, a QTL for increased sterile spikelet number was identified at the chromosome 7A locus, with increased numbers of infertile spikelets associated with increased numbers of fertile spikelets and the *WAPO-A1.hap2* haplotype. Therefore, while BMWpop RILs with the *WAPO-A1b* allele likely did result in some degree of source limitation, this did not manifest in the identification of QTL for grain morphometric traits at this locus.

### Additional multi-trait QTL identified in the BMWpop: chromosome 7B

We identified a multi-trait QTL involved in the control of two spikelet number traits on the long arm of chromosome 7B, at a homoeologous position to the chromosome 7A locus discussed above. However, the chromosome 7B locus explained a much lower amount of the phenotypic variation, ranging between 5 and 7%, and thus more typical of that commonly controlled by wheat yield component QTL. Although studies have identified spikelet number QTL at this location (e.g. Würschum et al. 2018), none to our knowledge have formally determined homoeology to the chromosome 7A locus. The peak SNP for the BMWpop chromosome 7B QTL was located very close to the candidate gene *WAPO-B1*, for which we identified two haplotypes in the BMWpop founders. The recent identification of *WAPO-A1* as underlying the chromosome 7A locus, our finding that alleles conferring higher spikelet number at the chromosome 7B QTL were associated with the *WAPO-B1.hap2* haplotype, and the presence of a non-conservative amino acid substitution in *WAPO-B1.hap2* at a highly conserved region of the protein, supports *WAPO-B1* as a strong candidate gene for the chromosome 7B QTL. That we were unable to amplify *WAPO-B1* in the founder Firl3565 warrants further investigation. However, the Firl3565 allele at the chromosome 7B locus conferred the strongest increase in spikelet number (Supplementary Table 6), and it is not immediately clear how the putative deletion of *WAPO-B1* might lead to this outcome, especially as loss-of-function mutation resulting in premature stop codons in the rice orthologue *APO1* lead to smaller inflorescences (Ikeda et al. 2007). Interestingly, our analysis of *WAPO-B1* genomic sequences in 16 hexaploid wheat lines with sequenced genomes identified *WAPO-B1.hap3* as possessing a 5 bp exonic deletion within the region coding for the F-box domain, leading to a frame shift and a truncated predicted protein. Although not present in the BMWpop, this haplotype was found in four of the 16 lines, including commercial spring wheat varieties from Australia and the International Maize and Wheat Improvement Center (CIMMYT). While further investigation is needed to determine the effect of *WAPO-B1.hap3* on spikelet number, it nevertheless indicates that putative non-functional *WAPO-B1* alleles can be tolerated in commercially viable wheat germplasm, thus not completely excluding the tentative hypothesis that *WAPO-B1* may be deleted in Firl3565. Finally, in silico analysis of *WAPO-B1* gene expression found it to be expressed in the apical meristem during the developmental phases in which final spikelet number is determined, supporting its role as underlying the spikelet-related QTL identified here. The expression of *WAPO-B1* in the grain tissues, which was particularly strong at the hard dough stage, indicates it may also play additional roles in the regulation of grain development, and warrants further investigation.

### Additional multi-trait QTL identified in the BMWpop: chromosome 5B

QTL for ‘flowering time’ and ‘ear length’ co-segregated at *QMtqtl.lfl-5B.1*. While flowering time is known to pleiotropically affect many yield component traits, the predicted allelic effects at this locus for these traits did not give a clear picture whether flowering was indirectly affecting ear length. Previous studies in the BMWpop have not identified a flowering time QTL at this location (Stadlmeier et al. 2019). However, although the flowering time QTL identified here by IM/CIM were classified as ‘weak’, they were identified in all three trials, as well as in the meta-analysis (Fig. 2). The presence of a flowering time QTL at this locus was further confirmed in all three trials by IBS (*p*_adj_ ≤ 0.0237) and by IBD in UK17 (*q*_*adj*_ = 0.0317) genetic analyses (Supplementary Table 6). The higher significance of IBS versus the IBD/CIM mapping methods observed at this QTL may be due to local factors across the locus (such as minor errors in marker order) reducing power of the haplotype-based genetic mapping approaches. Genes controlling flowering time determine the duration of specific developmental phases as the wheat plant matures, and this control is commonly in response to environmental queues (Dixon et al. 2018). Environment is known to affect developing inflorescence length (e.g. Friend 1965), and flowering time affects ear traits such as spikelet number and spikelet morphology (Dixon et al. 2018; Wolde et al. 2019; Worland et al. 1998). While it is feasible that flowering time may have a pleiotropic effect specifically on ear length, we are not aware of any previous reports demonstrating this. Lastly, while the cloned gene *Q* previously found to control wheat ear length is located on chromosome 5B (gene model *TraesCS5A02G473800*; Simons et al. 2006), it is situated > 120 Mb from *QMtqtl.lfl-5B.1* and so does not underlie the locus. For multi-trait QTL *QMtqtl.lfl-5B.2*, the QTL for ‘seed length/width ratio’ at this locus is presumably due to the co-locating QTL for ‘seed length’. While the predicted allelic founder effects for these underlying QTL at *QMtqtl.lfl-5B.2* were not always consistent for all eight founders between years/traits, the allele from the founder Format was notable in that it did consistently confer increased effect for both traits. A previous study using a bi-parental population between Format and another German variety, Pamier, identified a QTL controlling grain size characters at a similar location on chromosome 5B (based on anchoring common SNP marker *IWB39994* to the physical map: 695.660 Mb), with the increasing allele originating from Format (Mohler et al. 2016). However, while the chromosome 5B locus in the Format x Pamier population controlled thousand grain weight, seed width and seed area, no QTL was found in this population for seed length, making it unclear whether the QTL in these two populations are the same.

### Additional multi-trait QTL identified in the BMWpop: chromosome 6A

MT-QTL *QMtqtl.lfl-6A.2* on the long arm of chromosome 6A contained the highest number of constitutive co-locating QTL, including replicated QTL controlling four yield components (‘ear weight’, ‘number of seed per ear’, ‘seed area’ and ‘seed length’). All but two of the underlying QTL were identified in the UK trials, indicating the effects at this MT-QTL were predominantly environment specific. It is worth noting, resistance to the fungal diseases yellow rust (in UK18) and septoria tritici blotch (UK17) also co-located at the chromosome 6A locus and explained ~ 20% and 5% of the variation for disease severity, respectively (Supplementary Table 6). No obvious correlation was seen between the predicted allelic effects at the disease resistance and the yield component QTL. The founder carrying the most resistant yellow rust resistance allele (Potenzial) was also predicted to carry the most susceptible allele for septoria tritici blotch. Furthermore, while the chromosome 6A yellow rust QTL in UK18 co-located with many yield component QTL, the second major yellow rust resistance QTL on chromosome 1A, which also explained ~ 20% of the variation, did not. While the German trial was sprayed on one occasion with fungicide, this would not have protected the trial completely from disease. Together, these findings indicate that this region of chromosome 6A may contain allelic variation relevant to a number of agronomic traits. Indeed, yellow rust resistance loci have previously been identified at ~ 600 Mb on chromosome 6A (Muleta et al. 2017; Vazquez et al. 2012), as has a QTL for root architecture (e.g. Alahmad et al. 2019). Interestingly, our meta-analysis identified a flowering time QTL at *QMtqtl.lfl-6A.2*. While previous studies of septoria tritici blotch resistance in the BMWpop have not identified QTL on chromosome 6A, a QTL controlling flowering time has been reported at this location (*QEet.lfl-6A*, 250–252 cM) (Stadlmeier et al. 2019). Flowering plays a role in disease escape for yellow rust infection of the wheat ear (Cromey et al. 1989; Wellings 2003) and septoria tritici blotch (Eriksen et al. 2003; Stadlmeier et al. 2019). Flowering time can also pleiotropically affect yield component traits (reviewed by Cao et al. 2020), as potentially observed here for the flowering time QTL on chromosome 5B. However, the chromosome 6A flowering time QTL explained just 5% of the phenotypic variation, was not identified in the UK17 and DE18 trials, and the predicted allelic effects were not similar to those seen for the co-segregating yield-component QTL in the UK18 trial. While the balance of evidence suggests possible pleiotropic effects of the chromosome 6A disease resistance QTL on multiple yield component traits at *QMtqtl.lfl-6A.2*, this is by no means clear and warrants further investigation.

### Future directions for improved grain yield

Obvious next steps to investigate the QTL identified here would be to exploit the residual heterozygosity present in the BMWpop RILs to develop heterogeneous inbred families (HIFs), so Mendelizing the loci targeted. These HIFs could then be used for downstream investigation, such as detailed physiological characterization of the effects of discreet QTL, differential gene expression and fine-mapping. For multi-trait QTL, HIF materials could be used to further investigate whether co-location of QTL for multiple traits is due to linkage or pleiotropy. For example, by (1) generating recombination events within the target interval to segregate linked loci, or (2) by investigation of HIF materials predicted by to carry different allelic combinations for the target traits. In terms of understanding the genetics of how final grain yield is built across the lifecycle of wheat, approaches such as those employed here that define the genetic architecture controlling multiple constituent yield components can provide entry points into the genes and gene networks controlling yield (Brinton and Uauy 2019). This allows targeted selection for beneficial alleles, the mining of alternative alleles from wider germplasm, and ultimately to the creation of novel alleles at the underlying genes via artificial mutation techniques such as TILLING (Krasileva et al. 2017) and CRISPR/Cas9 gene editing (Shan et al. 2013). There is evidence that in high-yielding agricultural environments, elite cultivars are currently sink limited (Lynch et al. 2017). This indicates that molecular-assisted tracking of genetic loci that increase grains per ear and the manipulation of the genes that underlie them, such as the replicated spikelet number QTL identified here on chromosomes 1B, 3A, 6A, 7A and 7B, could play an important role in helping increase sink strength. A key trade-off in sink traits appears to be between thousand grain weight and spikelet fertility (which itself is composed of numerous traits, including the number of florets that produce grain within a spikelet and the number of spikelets per ear). In north-western Europe, there is evidence for an increasing trend in spikelet fertility over the last > 50 years, while over the same period no such trend is observed for thousand grain weight or spikelet number (Würschum et al. 2018). Therefore, the increase in fertility is likely attributed to the higher occurrence of grain set in more distal florets within the spikelet. At these positions, grain size is much smaller than at positions G1 and G2 (Feng et al. 2018). Carpel size has been reported to be positively correlated with final grain weight (Hasan et al. 2011; Xi et al. 2015). Whether reduced size in distal florets is due to source limitation during grain filling stage, and/or to reduced carpel size which is established at an earlier stage in plant development is not clear, and would be an interesting target for further exploration in the BMWpop. Either way, increasing grain set and size at G4 could be a clear breeding target to increase both thousand grain weight and grain yield. Exploring ways to achieve this, while simultaneously increasing spikelet number, by for example manipulation of *WAPO* genes on the A, B and D sub-genomes of hexaploid wheat, could therefore provide an approach to push yield potential beyond that currently achieved. Of course, attempts to push the hidden variation in polyploid wheat may have unforeseen consequences: for example, while *TaGW2* double mutants in bread wheat were associated with a greater increase in grain width, length and protein content, triple mutants were found to have wrinkled grain (Zhang et al. 2018a). Nevertheless, the identification of the genes that underlie sink-based yield component QTL, especially those that underlie multi-trait QTL such as those reported here, has promise to unlock further increases in yield potential. Finally, the incorporation of such molecular genetic advances into quantitative genetics methods such as genomic selection (Meuwissen et al. 2001; Jannink et al. 2010) may provide further opportunities for increasing the rate of genetic gain for wheat yield potential. It is likely that genomic selection will be a major source of genetic improvement in wheat breeding in the coming decade (Mackay et al. 2020). Therefore, incorporation of additional molecular genetic data on known genes or genetic loci, such as those identified in our study, may help improve genomic selection for final grain yield. Indeed, methods for the incorporation of such information are now emerging (e.g. Schrag et al. 2018), and exploration of these approaches may help to further increase the rate of genetic gain for wheat yield potential.

## Supplementary information

Supplementary Figure 1. Histograms of phenotypic values measured in the BMWpop for each of the 15 traits in the three trials, undertakein in the United Kingdom in 2017 (UK17), UK 2018 (UK18) and Germany 2018 (DE18). For each histogram, the BMWpop founder with the highest and lowest trait values are indicated by vertical dashed lines, colour coded as indicated in the key. (DOCX 10,466 kb)

Supplementary Figure 2. Analysis of ‘plant height’ quantitative trait locus (QTL) at the multi-trait QTL (MT-QTL) locus *QMtqtl.lfl-6A.1* on chromosome 6A. (A) ‘Plant height’ QTL from trials UK17, UK18 and DE18 mapping to the *QMtqtl.lfl-6A.1* locus. QTL plots shown used five co-variates (CIM-cov5). (B) A scatterplot of the genetic (Stadlmeier et al. 2018) versus physical (RefSeq v1.0. IWGSC 2018) maps of chromosome 6A. The map interval for *Rht24* we identified in the BMWpop is indicated in pink and bounded by the dashed red lines that meet the proximal and distal markers delimiting the QTL interval, and includes all of the intervening markers based on genetic map position, which are highlighted in red. The location of *TaGW2-A* (RefSeq v1.1 gene model *TraesCS6A02G189300*) is indicated by the blue circle on the y-axis. The region of low genetic recombination that spans the chromosome 6A centromere is indicated in grey and bounded by the black dashed lines. (DOCX 311 kb)

Supplementary Figure 3. Details of the multi-trait quantitative trait locus (QTL) *QMtqtl.lfl-7A.1* and the underlying candidate gene *WAPO-A1*. (A) Composite interval mapping (CIM) results for chromosome 7A for traits ‘total number of spikelets’ (totNFSP), ‘number of fertile spikelets’ (NFSP), ‘number of infertile spikelets’ (NISP), as well as closely linked QTL for ‘number of seed per ear’ (NS.NE) and ‘ear length’ (EL). Presented here are the meta-analysis predicted means analysed using CIM with inclusion of five covariates (CIM-cov5). (B) As for A, but excluding the highly significant QTL for totNSP and NFSP, allowing inspection of the non-significant peak for NISP (in grey) that coincides with the peak for totNSP and NFSP, the additional significant QTL for NISP spanning the region of low genetic recombination spanning the centromere (QTL peak at ~165 cM) and the closely linked, but potentially separate, QTL for NS.NE and EL. (C) *WAPO-A1* haplotypes, based on the DNA variants identified by comparing *WAPO-A1* sequences (including 1,000 bp up- and down-stream of the start and stop codons, respectively) from the wheat reference genome assembly (RefSeqv1.0. IWGSC 2018) with those from 15 additional hexaploid wheat lines with sequenced genomes (Walkowiak et al. 2020). The positions of the DNA variants that define the haplotypes are as detailed in Supplementary Table 8a, and their locations are numbered in relation to the *WAPO-A1* start codon in the RefSeq v1.0 assembly. Haplotype *WAPO-A1.hap1* and WAPO-hap2 have previously been shown to represent alleles conferring low (allele *WAPO-A1a*) and high (*WAPO-A1b*) spikelet number, respectively. The a H3 haplotype identified predominantly in wild and cultivated tetraploid emmer wheat by Kuzay et al. (2019) was not identified in the sequences analysed here, and so is not included in the figure. Sequencing a 1,719 bp region indicated by the dashed black line in the eight BMWpop founders allowed allocation as a (Ambition, BAY4535, Firl3565 and Julius) or *WAPO-A1.hap2* (Bussard, Event, Format and Potenzial). *WAPO-A1.hap1* and *WAPO-A1.hap2* were associated with the low and high spikelet number allele at QMtqtl-lfl.7A.1, respectively, and so are termed here as alleles *WAPO-A1a* and *WAPO-A1b*. Of the seven SNPs that differentiated these two haplotypes in the *WAPO-A1* region sequenced in the BMWpop founders, two lead to alterations of the predicted protein. The first was a T+140/G SNP that resulted in a F47/C amino acid substitution in a highly conserved region of the protein based on alignment of 57 proteins from 54 plant species. The second was a G+1284/A SNP resulting in an D384/N amino acid substitution located towards the C-terminus. (D) Weblogo plots illustrating amino acid conservation at the two locations for which DNA variants lead to amino acid substitutions in the predicted *WAPO-A1* protein, based on the alignment of the 57 protein accessions listed in Supplementary Table 3. (DOCX 489 kb)

Supplementary Figure 4. Genomic DNA alignment of *WAPO-A1*, including 1,000 bp up- and down-stream of the coding regions, from 16 hexaploid wheat lines with sequenced genome assemblies: T. aestivum varieties CS (Chinese Spring) (IWGSC, 2018), CDC_Stan (CDC Stanley), Claire, Mace, Norin 61, Weebill 1, ArinaLrFor, Cadenza, CDC_Land (CDC Landmark), Jagger, LongReach (LongReach Lancer), Paragon, Robigus, Julius, SY Matis and the T. aestivum ssp. spelta accession PI90962 (Walkowiak et al. 2020). Also included are the *WAPO-A1* genomic sequences generated by Sanger sequencing in the eight BMWpop founders (GenBank accessions MW366865 to MW366872). The positions of exon-1 and exon-2 are indicated by the blue and red lines, respectively. The region coding the F-box domain is indicated by the dashed black line. The 21 DNA variants identified in the sequence alignments are numbered, as also summarised in Supplementary Table 8a. Within the coding regions, DNA variants 10 and 15 result in amino acid substitutions F47/C and D384/N in the predicted protein, respectively. (DOCx 241 kb)

Supplementary Figure 5. Genomic DNA alignment of *WAPO-B1*, including 1,000 bp up- and down-stream of the coding regions, from 16 hexaploid wheat lines with sequenced genome assemblies: *T. aestivum* varieties CS (Chinese Spring) (IWGSC 2018), CDC_Stan (CDC Stanley), Claire, Mace, Norin 61, Weebill 1, Arina*LrFor*, Cadenza, CDC_Land (CDC Landmark), Jagger, LongReach (LongReach Lancer), Paragon, Robigus, Julius, SY Matis and the T. aestivum ssp. spelta accession PI90962 (Walkowiak et al. 2020). Also included are the *WAPO-B1* genomic sequences generated by Sanger sequencing in seven of the eight BMWpop founders (GenBank accessions MW366873 to MW366879). We were not able to PCR amplify *WAPO-B1* from the BMWpop founder Firl3565. The position of the (CT)n microsatellite upstream of the start codon is indicated by the green line. The positions of exon-1 and exon-2 are indicated by the blue and red lines, respectively. The region coding the F-box domain is indicated by the dashed black line. The 17 DNA variants identified in the sequence alignments are numbered, as also summarised in Supplementary Table 8b. Within the coding regions, DNA variants 5 (A+140/G) and 6 (G+427/A) result in amino acid substitutions H47/R and D143/N in the predicted protein, respectively. The 5 bp deletion within the region encoding the F-box domain in exon-1 (variant 4, present in LongReach Lancer, Mace, Weebill 1 and PI90962) results in a subsequent frame shift in the predicted protein, and a premature stop codon (TAA) at the position indicated by the black triangle. *Variant 6 (G+427/A) is present only in *WAPO-B1.hap3* (LongReach Lancer, Mace, Weebill 1 and PI90962) and encodes for a glutamine (Q) residue; however, in this haplotype the preceding 5 bp exon-1 deletion means that the amino acid sequence at this point in the predicted protein has already been knocked out of frame. $Variant 7 (G+517/T) results in amino acid change A173/S in *WAPO-B1.hap.2*, while in *WAPO-B1.hap3*, where the protein has already been knocked out of frame, it results in a valine (V) residue. (DOCX 269 kb)

Supplementary Figure 6. Alignment of the predicted proteins identified as ‘orthologous’ to WAPO-B1 in the Ensembl Plants database. Details of the 56 proteins sourced from 53 species are listed in Supplementary Table 4. Where more than one protein sequence was identified in a given species, only the first was used here, except for polyploid *Triticum* species where all sequences identified as ‘orthologues’ were included. The locations of the F-box domain and the two amino acid substitutions resulting from DNA variants identified in our *WAPO-B1* haplotype analysis (detailed in Supplementary Table 8b) are indicated. Species abbreviations: Ach (*Actinidia chinensis*), Aet (*Aegilops tauschii*), Atr (*Amborella trichopoda*), Aco (*Ananas comosus*), Ath (*Arabidopsis thaliana*), Aap (*Arabis alpine*), Bvu (*Beta vulgaris*), Bdi (*Brachypodium distachyon*), Bna (*Brassica napus*), Bol (*Brassica oloracea*), Bra (*Brassica rapa*), Camsa (*Camelina sativa*), Cansa (*Cannabis sativa*), Can (*Caspicum annum*), Cla (*Citrullus lanatus*), Ccl (*Citrus clementina*), Cofca (*Coffea canephora*), Ccap (*Corchorus capsularis*), Cme (*Cucumis melo*), Csa (*Cucumis sativus*), Cca (*Cynara cardunculus*), Dca (*Daucus carota*), Dro (*Dioscorea rotundata*), Gma (*Glycine max*), Han (*Helianthus annuus*), Hvu (*Hordeum vulgare*), Itr (*Ipomoea triloba*), Lan (*Lupinus angustifolius*), Mdo (*Malus domestica*), Mes (*Manihot esculenta*), Mtr (*Medicago truncatula*), Nat (*Nicotiana attenuate*), Oer (*Olea europaea*), Osa (*Oryza sativa*), Pvu (*Phaseolus vulgaris*), Pve (*Pistacia vera*), Ptr (*Populus trichocarpa*), Pav (*Prunus avium*), Ppe (*Prunus persica*), Pu (*Prunus dulcis*), Rch (*Rosa chinensis*), Smo (*Selaginella moellendorffii*), Sly (*Solanum lycopersicum*), Stu (*Solanum tuberosum)*, Tca (*Theobroma cacao*), Tpr (*Trifolium pratense*), Tae (*Triticum aestivum*; A-, B- and D-genome homoeologue indicated), Tdi (*Triticum dicoccoides*; A- and B-genome homoeologue indicated), Ttu (*Triticum turgidum*; B-genome homoeologue indicated), Van (*Vigna angularis*), Vra (*Vigna radiata*), Vvi (*Vitis vinifera*), Zma (*Zea mays*). (DOCX 290 kb)

Supplementary Figure 7. Heatmap of *WAPO-A1, -B1* and -*D1* gene expression in different wheat tissues at different developmental stages. Data was sourced from the wheat gene expression atlas (Ramírez-González et al. 2018). TPM = transcripts per million. (DOCX 257 kb)

Supplementary Table 1. Disease scoring guide used to assess yellow rust (YR) and septoria tritici blotch (STB) infection on a percentage scale in the BMWpop. Assessment was at the plot level and based on disease present in the top four leaves. (DOCX 14 kb)

Supplementary Table 2. Trait correlations. (A) Trait correlations between trials conducted in the United Kingdom in 2017 (UK17), in the UK in 2018 (UK18) and in Germany in 2018 (DE18), and (B) their corresponding significance values. *P*-values ≤ 0.05 are indicated in bold. Trait abbreviations: EL (ear length), EW (ear width), NFSP (number of fertile spikelets per ear), NISP (number of infertile spikelets per ear), totNSP (total number of spikelets per ear), NS.NE (number of seeds per ear), WS.EW (seed weight/ear weight ratio), SA (seed area), SWI (seed width), SL (seed length), FFD (factor form density), SL.SWI (seed length/seed width ratio), FT (flowering time), HT (plant height), TGW (thousand grain weight). (XLSX 49 kb)

Supplementary Table 3. Primers used to PCR amplify and Sanger sequence *WAPO-A1* and *WAPO-B1* in the BMWpop founders. Three primer pairs were used to attempt to amplify *WAPO-B1* in the founder Firl3565: WAPOB1_49F_UFO/WAPOB1_49R_UFO, WAPOB1_2F/WAPOB1_3R and WAPOB1_4F/WAPOB1_49R_UFO. PCR was undertaken using the following conditions: 9 min at 96 °C followed by 36 cycles of 96 °C for 45 secs, 58 °C for 45 secs and 72 °C for 120 secs, with a final extension stage of 72 °C for 7 mins. (DOCX 15 kb)

Supplementary Table 4. Protein sequences from plant species with sequenced genomes identified as ‘orthologues’ of WAPO-A1/-B1 in Ensembl Plants, and used for sequence alignments and analysis of conserved domains. Proteins were excluded if they had deletions within the investigated regions, or if they had sequence similarity with WAPO-A1/-B1 of < 20%. Where more than one protein sequence was identified in a given species, only the most similar to WAPO-A1/-B1 was used here (*except for polyploid *Triticum* species, where all sequences identified as ‘orthologues’ were included). †Not used in the Weblogo for the WAPO-A1 D384/N amino acid substitution, due to misalignments within the analysed region. (DOCX 21 kb)

Supplementary Table 5. Best linear unbiased estimates (BLUEs) for the 15 traits measured in BMWpop grown in field trials in the United Kingdom in 2017 and 2018 (UK17, UK18) and Germany in 2018 (DE18). Also included are the meta-analysis BLUEs (_meta). Trait abbreviations: EL (ear length), EW (ear width), NFSP (number of fertile spikelets per ear), NISP (number of infertile spikelets per ear), totNSP (total number of spikelets per ear), NS.NE (number of seeds per ear), WS.EW (seed weight/ear weight ratio), SA (seed area), SWI (seed width), SL (seed length), FFD (factor form density), SL.SWI (seed length/seed width ratio), FT (flowering time), HT (plant height), TGW (thousand grain weight). (XLSX 197 kb)

Supplementry Table 6. Summary of all quantitative trait loci (QTL) identified in the three field trials undertaken: two trials in the United Kingdom in 2017 and 2018 (UK17 and UK18) and one trial in Germany in 2018 (DE18), as well as in the meta-analysis (Meta). The QTLs reported are based on the results of interval mapping (IM), with QTL intervals determined based on the results of composite interval mapping using five covariates (CIM-cov5). For these QTL, also listed are the results of genetic mapping using CIM-cov10, identity-by-SNP (IBS) and identity-by-descent (IBD) approaches. The permutated 1% and 5% trait-specific significance thresholds are indicated, along with the percentage of the phenotypic variation explained (pve) and the trait heritability (h2). Trait abbreviations: EL (‘ear length’), EW (‘ear width’), NFSP (‘number of fertile spikelets per ear’), NISP (‘number of infertile spikelets per ear’), totNSP (‘total number of spikelets per ear’), NS.NE (‘number of seeds per ear’), WS.EW (‘seed weight-ear weight ratio’), SA (seed area), SWI (‘seed width’), SL (‘seed length’), FFD (‘factor form density’), SL.SWI (‘seed length-seed width ratio’), FT (‘flowering time’), HT (‘plant height’), TGW (‘thousand grain weight’). Genetic map positions are sourced from Stadlmeier et al. (2018). Wheat physical map positions are from the wheat reference genome assembly, RefSeq v1.0 (IWGSC 2018). Predicted allelic effects of founders 1 (Event), 2, (BAYP4535), 3 (Ambition), 4 (Firl3565), 5 (Format), 6 (Potenzial) and 7 (Bussard) are relative to founder 8 (Julius). The location of QTL within, outside or partially overlapping with the highly non-recombining regions of the chromosomes, based on the BMWpop genetic map versus the RefSeq v1.0 wheat genome assembly (IWGSC 2018), is indicated as ‘Y’, ‘N’ or ‘P’, respectively. Multi-trait QTL (MT-QTL) consist of co-locating QTL for traits identified in two or more test environments, and named *QMtqtl.lfl-XX*, where *XX* represents chromosome number. $Component QTL indicated in brackets are located within the MT-QTL, but were identified in only one test environment. †Un = anchored to IWGSC RefSeq v1.0 contigs unassigned to a chromosome. NA = no BLASTn hit on target chromosome. QTL for fungal disease susceptibility are shown separately at the end of the table. As septoria tritici blotch QTL were not identified using IM, the results presented are for CIM-cov5. (XLSX 115 kb)

Supplementary Table 7. Summary information for the alleles present in the BMWpop founders at *Rht-B1* and *Rht-D1*, and the haplotypes at *WAPO-A1* and *WAPO-B1*. Alleles at the *Rht-B1* and *Rht-D1* genes are based on the diagnostic genetic markers TG0010 and TG0011, as genotyped by Stadlmeier et al. (2018). Their effect on phenotype are indicated in brackets. Haplotypes at the *WAPO-A1* and *WAPO-B1* genes that are located within the intervals of BMWpop Meta-QTL *QMtqtl.lfl-7A.1* (traits: ‘number of infertile spikelets’, ‘number of fertile spikelets’, ‘total number of spikelets’ and ‘ear length’) and *QMtqtl.lfl-7B.1* (traits: ‘number of fertile spikelets’ and ‘total number of spikelets’), respectively, are listed. Their haplotypes were determined as detailed in Supplementary Table 8. Allelic effect of these genetic loci on spikelet number related traits are indicated in brackets as ‘high’ or ‘low’, based on the predicted allelic effects at the QTL summarised in Supplementary Table 6. *As we were unable to amplify *WAPO-B1* from founder Firl3565, its *WAPO-B1* haplotype is currently unconfirmed. (DOCX 16 kb)

Supplementary Table 8. Summary of DNA variants in and around *WAPO-A1 *and *WAPO-B1* in 23 hexaploid wheat lines. The positions of DNA variants are relative to the beginning of the start codon in the wheat reference genome assembly (RefSeq v1.0. IWGSC 2018) and the corresponding gene models (build Refseq v1.2), generated in the variety Chinese Spring. Data for 15 wheat lines were extracted from the genome assemblies published by Walkowiak et al. (2020) and generated within the 10 Plus Wheat Genomes Project (10PWG). Line PI190962 is an accession of hexaploid spelt wheat (*Triticum aestivum ssp. spelta*), all other lines are hexaploid bread wheat lines. For the 10PWG lines, the genomic sequences extracted included 1,000 bp up- and down-stream of the start and stop codons, respectively. Where DNA variants in the 10PWG lines and Chinese Spring could not be called due to assembly gaps, this is indicated in the table by ‘N’. For the BMWpop founder *WAPO-A1 *and *WAPO-B1* genomic sequences generated in this study (GenBank accessions MW366865 to MW366879), variant sites located outside of the regions we sequenced are indicated as ‘N’. The effects of DNA variants on the predicted WAPO proteins are indicated. Syn = synonymous substitution; FS, stop (frame-shift followed by a premature stop codon); N/A = not applicable. *WAPO-A1 *and *WAPO-B1* haplotype designations are indicated. *For the BMWpop founders, haplotypes are assigned based on the 1,719 bp and 1,893 bp regions sequenced for *WAPO-A1 *and *WAPO-B1*, respectively. The variety Julius is included twice in the tables: the first entry is from the 10PWG collection, while the second is the accession used in the construction of the BMWpop (listed here as Julius_BMW). (A) WAPO-A1. (B) *WAPO-B1*. As Chinese Spring contained a region of missing sequence in 5’ region upstream of the *WAPO-B1* start codon (located immediately adjacent to a (CT)n microsatellite repeat), we extracted just 444 bp of the upstream region. Using the three primer combinations described in Supplementary Table 2, we were not able to amplify *WAPO-B1* in BMWpop founder Firl3565, and the *WAPO-B1* haplotype and all SNP sites are indicated as ‘NA’. Allelic effect at QTL *QTotnsp.lfl-7B.1* that spans *WAPO-B1* are indicated for the BMWpop founders as determined from the data listed from the meta-analysis as listed in Supplementary Table 6, and summarised here as ‘low’ (reducing effect on spikelet number) or ‘high’ (increasing effect on spikelet number). $Indicated is the amino acid change in the predicted protein encoded by *WAPO-B1.hap1* and *WAPO-B1.hap2* only, as *WAPO-B1.hap3* contains a deletion leading to a frameshift which affects the protein sequence at these sites. (XLSX 23 kb)

Supplementary Notes 1 (DOCX 17 kb)

## Data Availability

Phenotypic data (BLUEs) are available in the Supplementary Materials. The BMWpop genotypic data is previously published (Stadlmeier et al. 2018).
