# Supplementary material for: Identification of eight QTL controlling multiple yield components in a German multi-parental wheat population, including Rht24, WAPO-A1, WAPO-B1 and genetic loci on chromosomes 5A and 6A
Source: Theor Appl Genet. 2021 Mar 12;134(5):1435–54. doi: 10.1007/s00122-021-03781-7 (PMC8081691; doi:10.1007/s00122-021-03781-7)
Supplement: Supplementary file 1 — Supplementary Figure 1. Histograms of phenotypic values measured in the BMWpop for each of the 15 traits in the three trials, undertakein in the United Kingdom in 2017 (UK17), UK 2018 (UK18) and Germany 2018 (DE18). For each histogram, the BMWpop founder with the highest and lowest trait values are indicated by vertical dashed lines, colour coded as indicated in the key. (DOCX 10,466 kb) [file 122_2021_3781_MOESM1_ESM.docx]

**
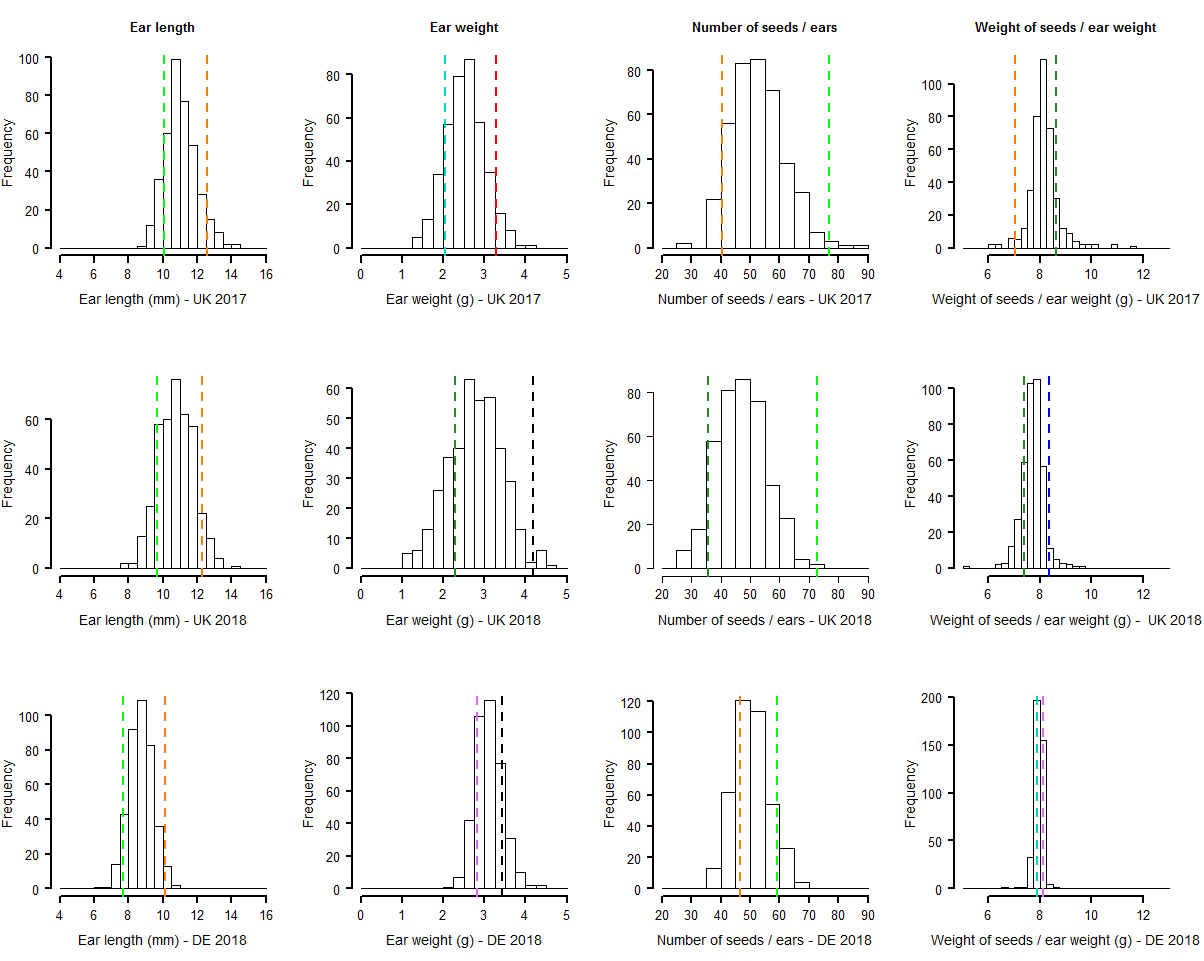

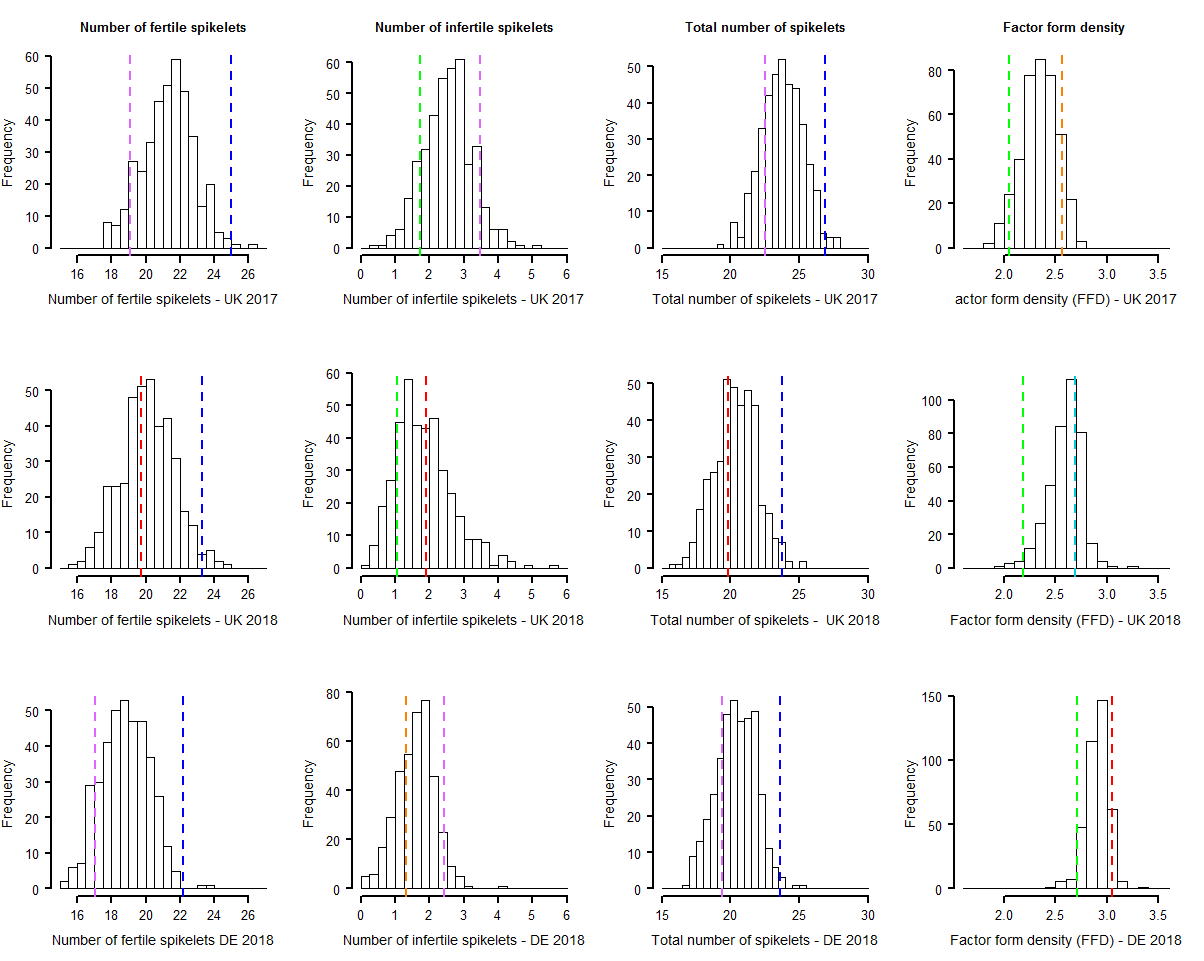

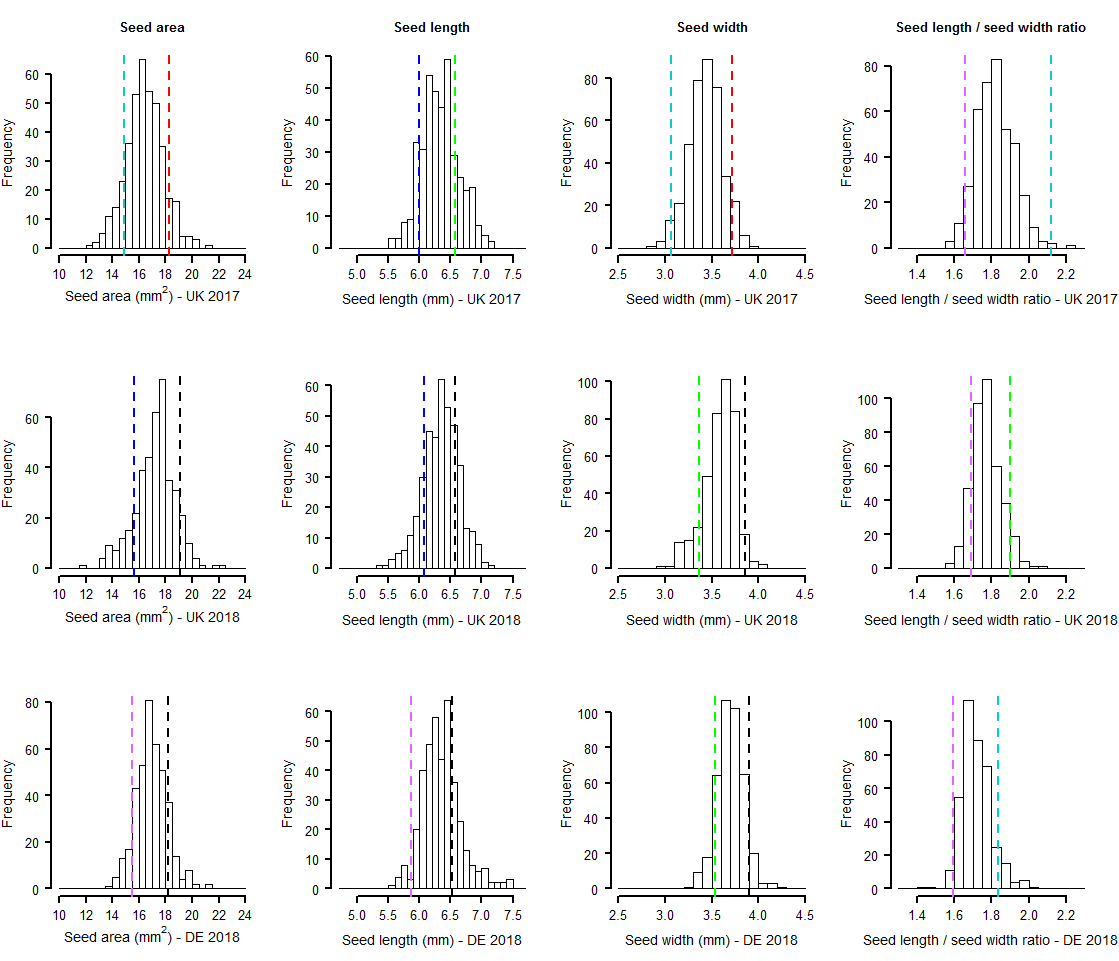
**
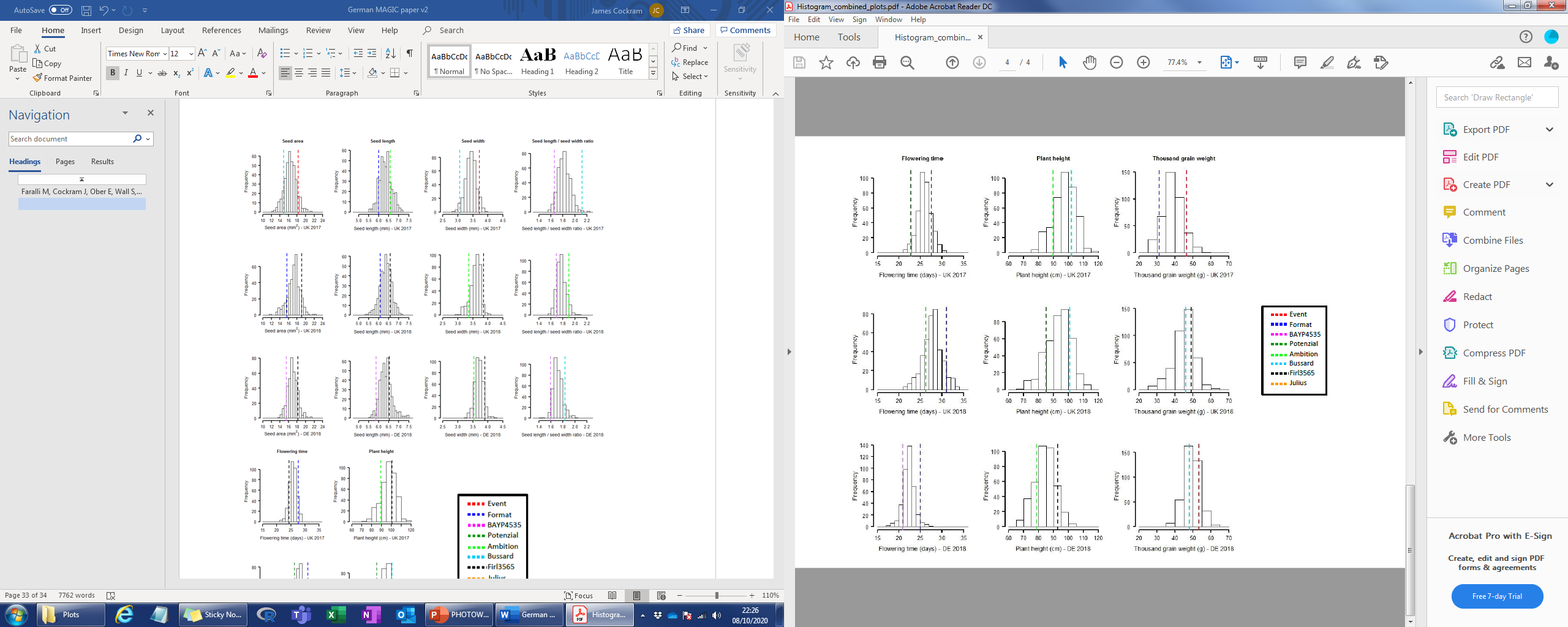


**Supplementary Figure 1.** Histograms of phenotypic values measured in the BMWpop for each of the 15 traits in the three trials, undertaken in in the United Kingdom in 2017 (UK17), UK 2018 (UK18) and Germany 2018 (DE18). For each histogram, the BMWpop founder with the highest and lowest trait values are indicated by vertical dashed lines, colour coded as indicated in the key.
