# Supplementary material for: Identification of eight QTL controlling multiple yield components in a German multi-parental wheat population, including Rht24, WAPO-A1, WAPO-B1 and genetic loci on chromosomes 5A and 6A
Source: Theor Appl Genet. 2021 Mar 12;134(5):1435–54. doi: 10.1007/s00122-021-03781-7 (PMC8081691; doi:10.1007/s00122-021-03781-7)
Supplement: Supplementary file 2 — Supplementary Figure 2. Analysis of ‘plant height’ quantitative trait locus (QTL) at the multi-trait QTL (MT-QTL) locus QMtqtl.lfl-6A.1 on chromosome 6A. (A) ‘Plant height’ QTL from trials UK17, UK18 and DE18 mapping to the QMtqtl.lfl-6A.1 locus. QTL plots shown used five co-variates (CIM-cov5). (B) A scatterplot of the genetic (Stadlmeier et al. 2018) versus physical (RefSeq v1.0. IWGSC 2018) maps of chromosome 6A. The map interval for Rht24 we identified in the BMWpop is indicated in pink and bounded by the dashed red lines that meet the proximal and distal markers delimiting the QTL interval, and includes all of the intervening markers based on genetic map position, which are highlighted in red. The location of TaGW2-A (RefSeq v1.1 gene model TraesCS6A02G189300) is indicated by the blue circle on the y-axis. The region of low genetic recombination that spans the chromosome 6A centromere is indicated in grey and bounded by the black dashed lines. (DOCX 311 kb) [file 122_2021_3781_MOESM2_ESM.docx]

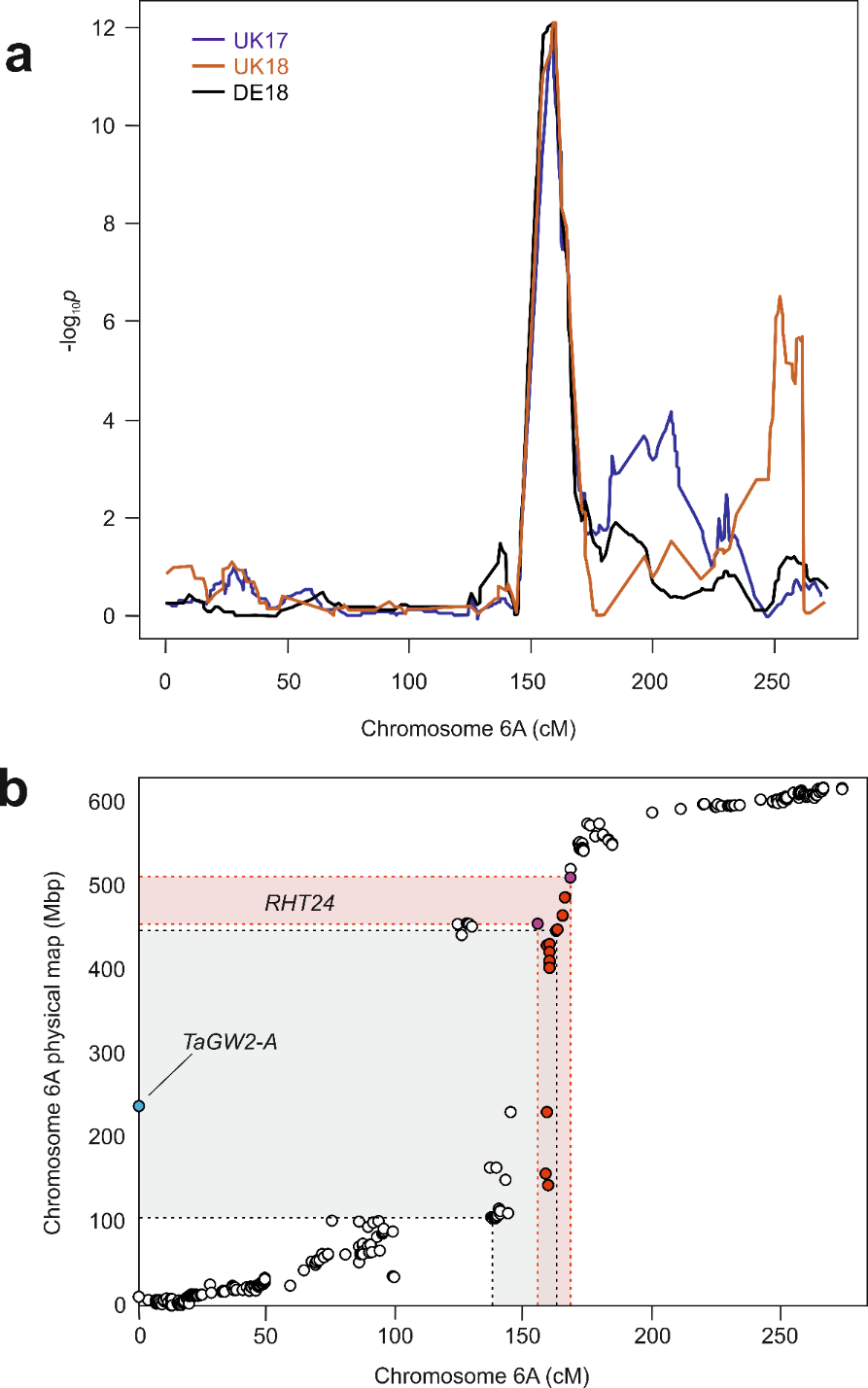


**Supplementary Figure 2.** Analysis of ‘plant height’ quantitative trait locus (QTL) at the multi-trait QTL (MT-QTL) locus *QMtqtl.lfl-6A.1* on chromosome 6A. (A) ‘Plant height’ QTL from trials UK17, UK18 and DE18 mapping to the *QMtqtl.lfl-6A.1* locus. QTL plots shown used five co-variates (CIM-cov5). (B) A scatterplot of the genetic (Stadlmeier et al. 2018) versus physical (RefSeq v1.0. IWGSC 2018) maps of chromosome 6A. The map interval for *Rht24* we identified in the BMWpop is indicated in pink and bounded by the dashed red lines that meet the proximal and distal markers delimiting the QTL interval, and includes all of the intervening markers based on genetic map position, which are highlighted in red. The location of *TaGW2-A* (RefSeq v1.1 gene model *TraesCS6A02G189300*) is indicated by the blue circle on the y-axis. The region of low genetic recombination that spans the chromosome 6A centromere is indicated in grey and bounded by the black dashed lines.
