# Supplementary material for: Identification of eight QTL controlling multiple yield components in a German multi-parental wheat population, including Rht24, WAPO-A1, WAPO-B1 and genetic loci on chromosomes 5A and 6A
Source: Theor Appl Genet. 2021 Mar 12;134(5):1435–54. doi: 10.1007/s00122-021-03781-7 (PMC8081691; doi:10.1007/s00122-021-03781-7)
Supplement: Supplementary file 3 — Supplementary Figure 3. Details of the multi-trait quantitative trait locus (QTL) QMtqtl.lfl-7A.1 and the underlying candidate gene WAPO-A1. (A) Composite interval mapping (CIM) results for chromosome 7A for traits ‘total number of spikelets’ (totNFSP), ‘number of fertile spikelets’ (NFSP), ‘number of infertile spikelets’ (NISP), as well as closely linked QTL for ‘number of seed per ear’ (NS.NE) and ‘ear length’ (EL). Presented here are the meta-analysis predicted means analysed using CIM with inclusion of five covariates (CIM-cov5). (B) As for A, but excluding the highly significant QTL for totNSP and NFSP, allowing inspection of the non-significant peak for NISP (in grey) that coincides with the peak for totNSP and NFSP, the additional significant QTL for NISP spanning the region of low genetic recombination spanning the centromere (QTL peak at ~165 cM) and the closely linked, but potentially separate, QTL for NS.NE and EL. (C) WAPO-A1 haplotypes, based on the DNA variants identified by comparing WAPO-A1 sequences (including 1,000 bp up- and down-stream of the start and stop codons, respectively) from the wheat reference genome assembly (RefSeqv1.0. IWGSC 2018) with those from 15 additional hexaploid wheat lines with sequenced genomes (Walkowiak et al. 2020). The positions of the DNA variants that define the haplotypes are as detailed in Supplementary Table 8a, and their locations are numbered in relation to the WAPO-A1 start codon in the RefSeq v1.0 assembly. Haplotype WAPO-A1.hap1 and WAPO-hap2 have previously been shown to represent alleles conferring low (allele WAPO-A1a) and high (WAPO-A1b) spikelet number, respectively. The a H3 haplotype identified predominantly in wild and cultivated tetraploid emmer wheat by Kuzay et al. (2019) was not identified in the sequences analysed here, and so is not included in the figure. Sequencing a 1,719 bp region indicated by the dashed black line in the eight BMWpop founders allowed allocation as a (Ambition, BAY4535, Firl3 [file 122_2021_3781_MOESM3_ESM.docx]

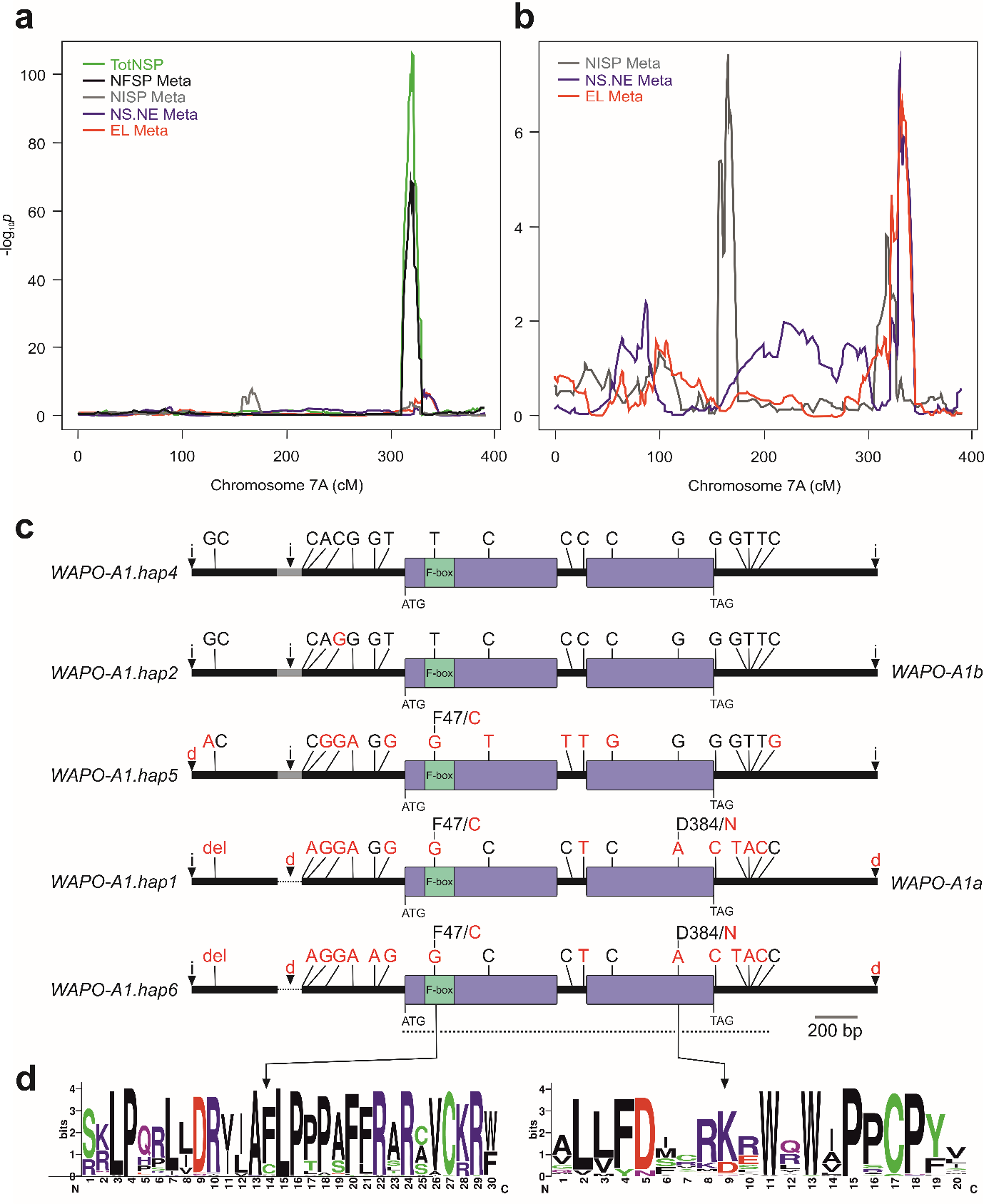


**Supplementary Figure 3.** Details of the multi-trait quantitative trait locus (QTL) *QMtqtl.lfl-7A.1* and the underlying candidate gene *WAPO-A1*. (A) Composite interval mapping (CIM) results for chromosome 7A for traits ‘total number of spikelets’ (totNFSP), ‘number of fertile spikelets’ (NFSP), ‘number of infertile spikelets’ (NISP), as well as closely linked QTL for ‘number of seed per ear’ (NS.NE) and ‘ear length’ (EL). Presented here are the meta-analysis predicted means analysed using CIM with inclusion of five covariates (CIM-cov5). (B) As for A, but excluding the highly significant QTL for totNSP and NFSP, allowing inspection of the non-significant peak for NISP (in grey) that coincides with the peak for totNSP and NFSP, the additional significant QTL for NISP spanning the region of low genetic recombination spanning the centromere (QTL peak at ~165 cM) and the closely linked, but potentially separate, QTL for NS.NE and EL. (C) *WAPO-A1* haplotypes, based on the DNA variants identified by comparing *WAPO-A1* sequences (including 1,000 bp up- and down-stream of the start and stop codons, respectively) from the wheat reference genome assembly (RefSeqv1.0. IWGSC 2018) with those from 15 additional hexaploid wheat lines with sequenced genomes (Walkowiak et al. 2020). The positions of the DNA variants that define the haplotypes are as detailed in Supplementary Table 8a, and their locations are numbered in relation to the *WAPO-A1* start codon in the RefSeq v1.0 assembly. Haplotype *WAPO-A1.hap1* and *WAPO-hap2* have previously been shown to represent alleles conferring low (allele *WAPO-A1a*) and high (*WAPO-A1b*) spikelet number, respectively. The *WAPO-A1* H3 haplotype identified predominantly in wild and cultivated tetraploid emmer wheat by Kuzay et al. (2019) was not identified in the sequences analysed here, and so is not included in the figure. Sequencing a 1,719 bp region indicated by the dashed black line in the eight BMWpop founders allowed allocation as *WAPO-A1.hap1* (Ambition, BAY4535, Firl3565 and Julius) or *WAPO-A1.hap2* (Bussard, Event, Format and Potenzial). *WAPO-A1.hap1* and *WAPO-A1.hap2* were associated with the low and high spikelet number allele at *QMtqtl-lfl.7A.1*, respectively, and so are termed here as alleles *WAPO-A1a* and *WAPO-A1b*. Of the seven SNPs that differentiated these two haplotypes in the *WAPO-A1* region sequenced in the BMWpop founders, two lead to alterations of the predicted protein. The first was a T+140/G SNP that resulted in a F47/C amino acid substitution in a highly conserved region of the protein based on alignment of 57 proteins from 54 plant species. The second was a G+1284/A SNP resulting in an D384/N amino acid substitution located towards the C-terminus. (D) Weblogo plots illustrating amino acid conservation at the two locations for which DNA variants lead to amino acid substitutions in the predicted WAPO-A1 protein, based on the alignment of the 57 protein accessions listed in Supplementary Table 3.
