# Supplementary material for: Identification of eight QTL controlling multiple yield components in a German multi-parental wheat population, including Rht24, WAPO-A1, WAPO-B1 and genetic loci on chromosomes 5A and 6A
Source: Theor Appl Genet. 2021 Mar 12;134(5):1435–54. doi: 10.1007/s00122-021-03781-7 (PMC8081691; doi:10.1007/s00122-021-03781-7)
Supplement: Supplementary file 4 — Supplementary Figure 4. Genomic DNA alignment of WAPO-A1, including 1,000 bp up- and down-stream of the coding regions, from 16 hexaploid wheat lines with sequenced genome assemblies: T. aestivum varieties CS (Chinese Spring) (IWGSC, 2018), CDC_Stan (CDC Stanley), Claire, Mace, Norin 61, Weebill 1, ArinaLrFor, Cadenza, CDC_Land (CDC Landmark), Jagger, LongReach (LongReach Lancer), Paragon, Robigus, Julius, SY Matis and the T. aestivum ssp. spelta accession PI90962 (Walkowiak et al. 2020). Also included are the WAPO-A1 genomic sequences generated by Sanger sequencing in the eight BMWpop founders (GenBank accessions MW366865 to MW366872). The positions of exon-1 and exon-2 are indicated by the blue and red lines, respectively. The region coding the F-box domain is indicated by the dashed black line. The 21 DNA variants identified in the sequence alignments are numbered, as also summarised in Supplementary Table 8a. Within the coding regions, DNA variants 10 and 15 result in amino acid substitutions F47/C and D384/N in the predicted protein, respectively. (DOCx 241 kb) [file 122_2021_3781_MOESM4_ESM.docx]

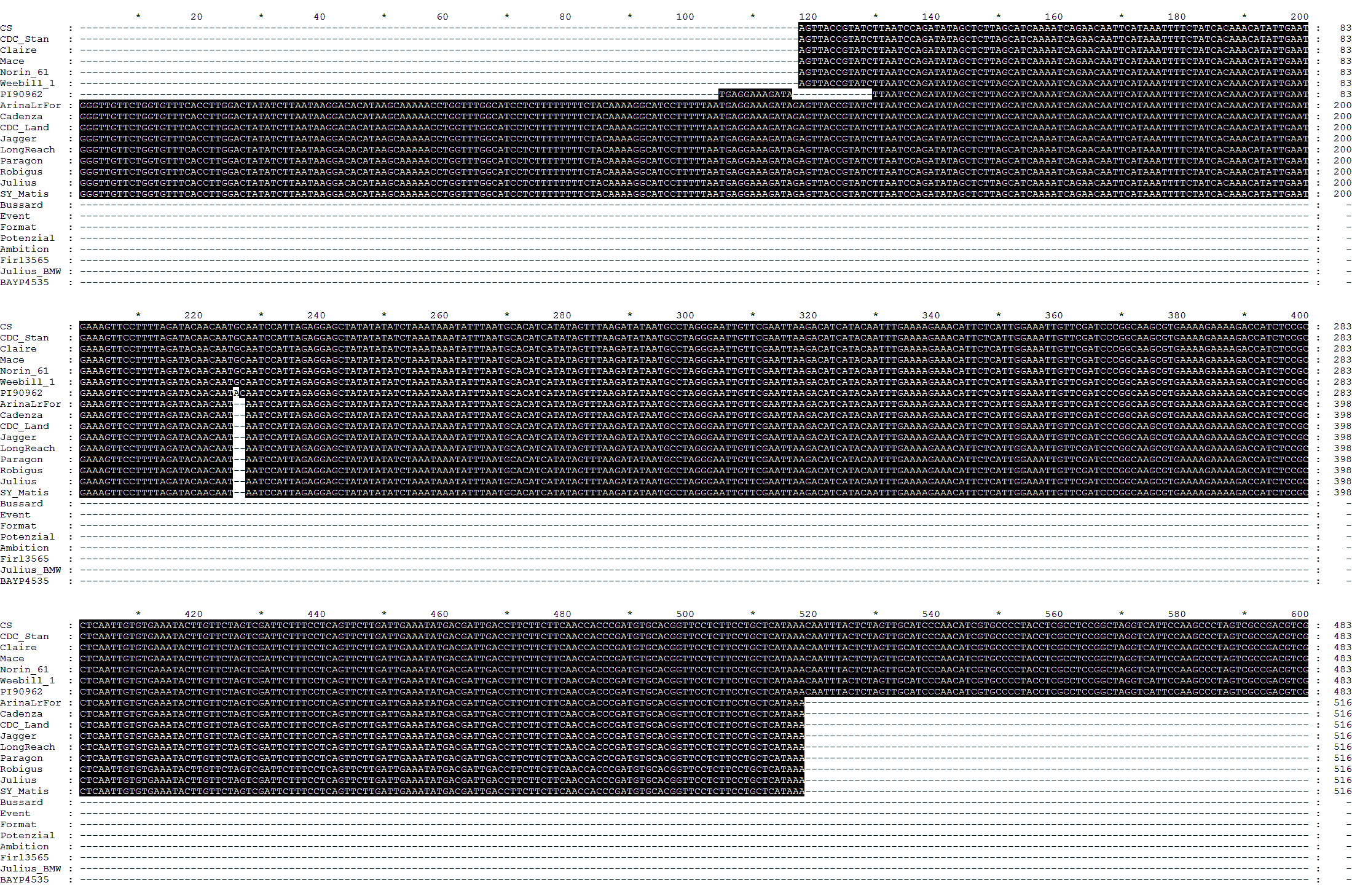


3

2

1


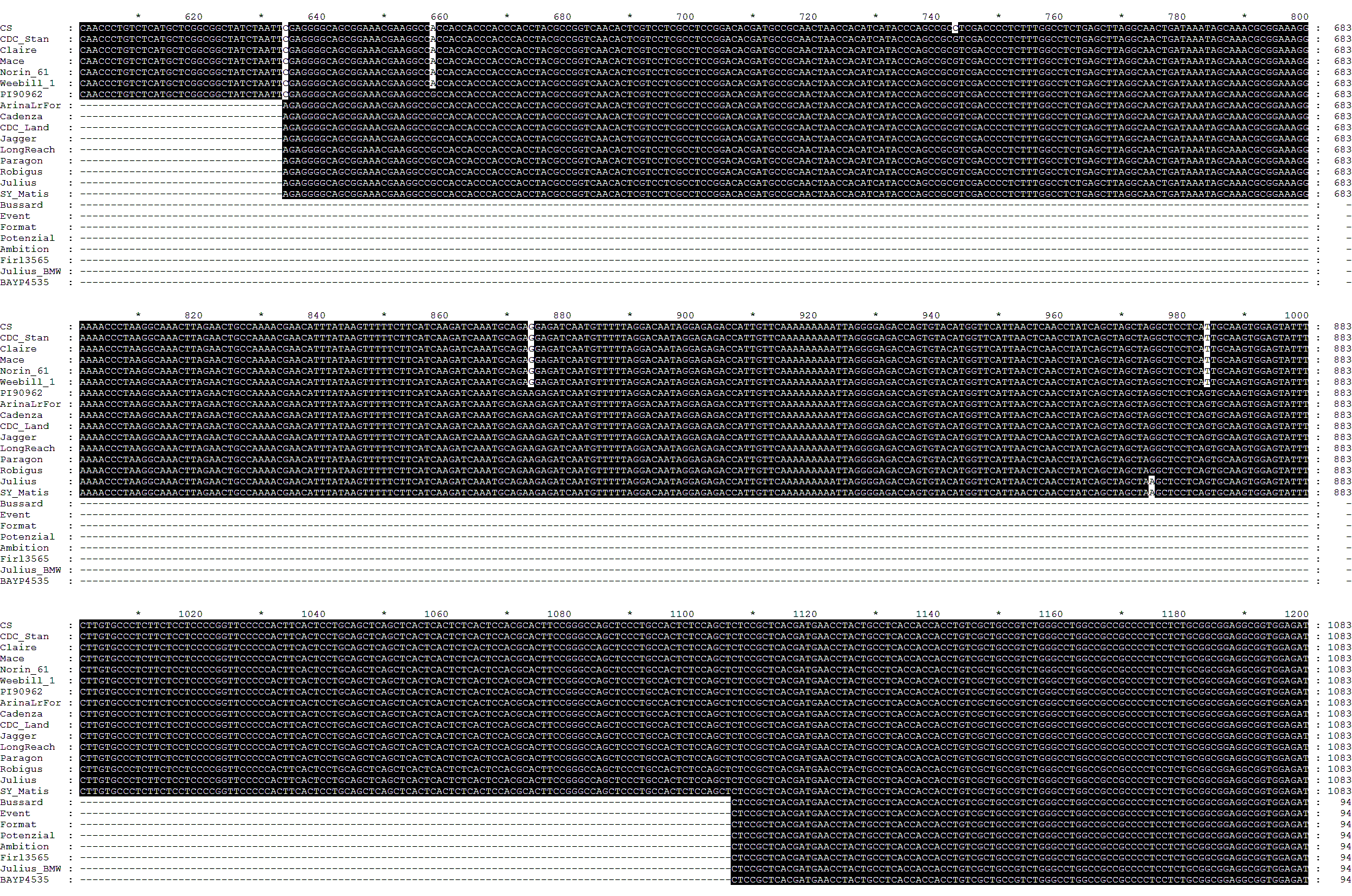


7 8 9

6

5

4


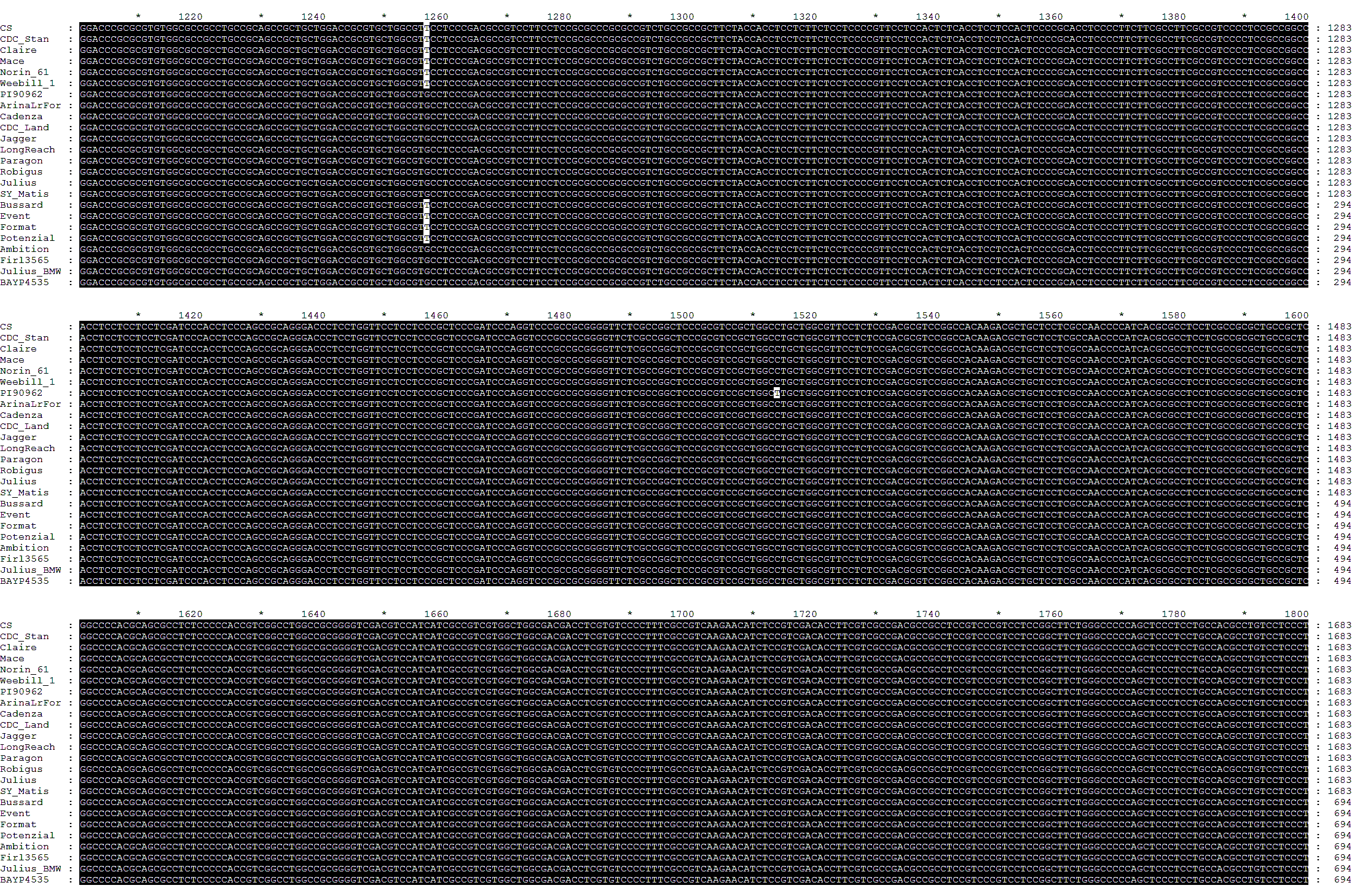


10 (T+140/G 🡪 F47/C)

11


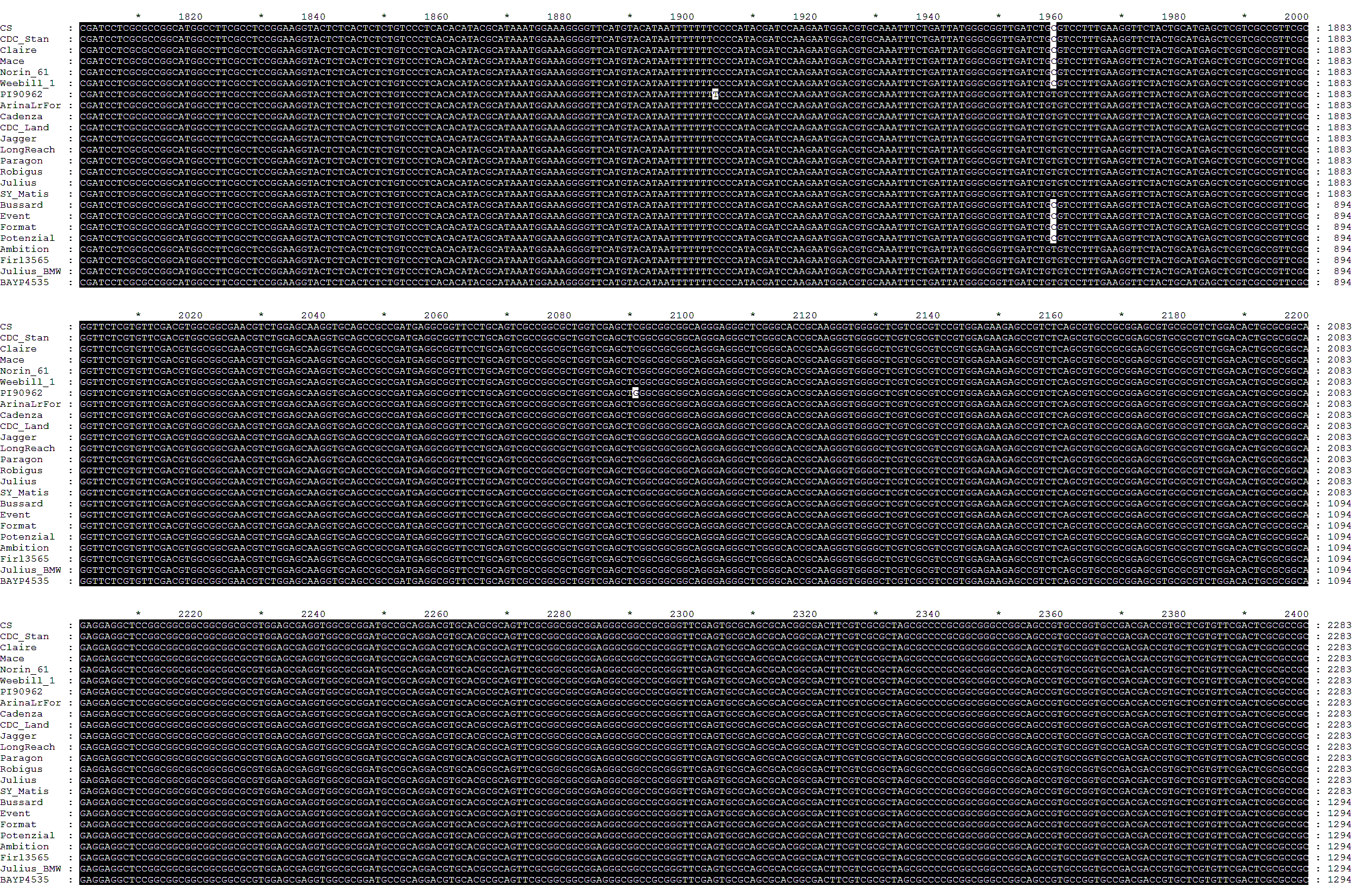


14

12 13


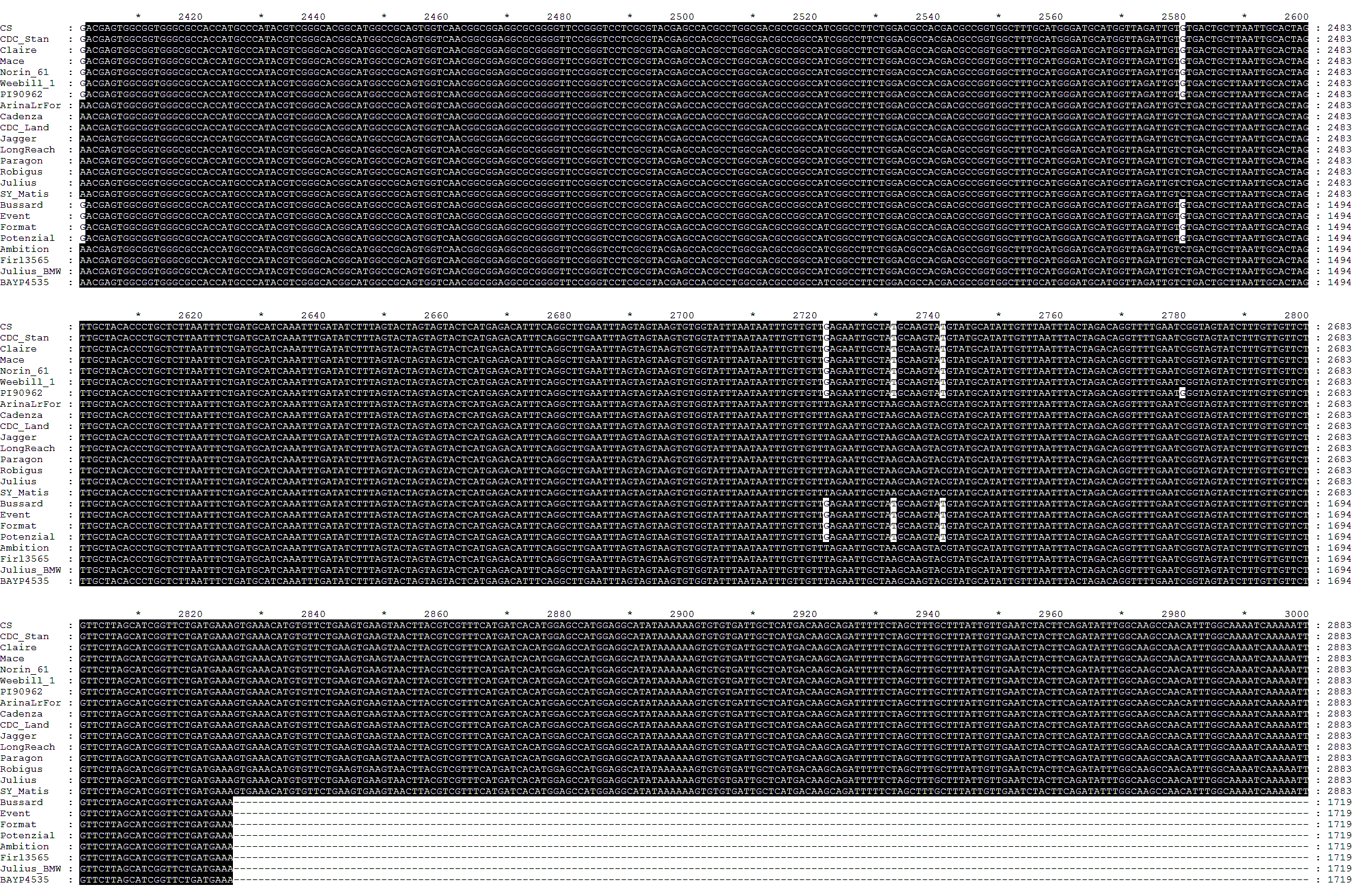


15 (G+1284/A 🡪 D384/N)

20

19

18

17

16


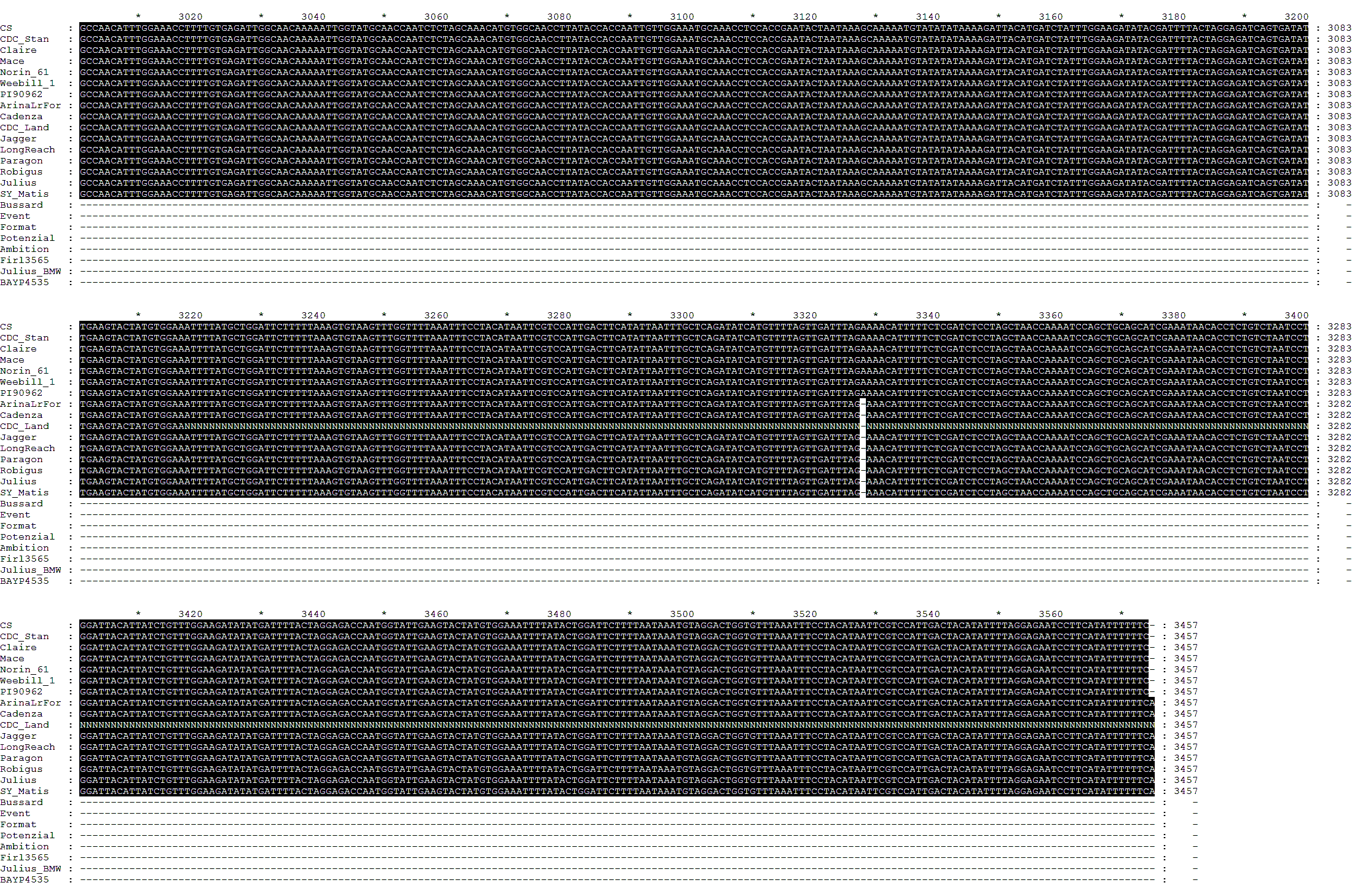


21

**Supplementary Figure 4.** Genomic DNA alignment of *WAPO-A1*, including 1,000 bp up- and down-stream of the coding regions, from 16 hexaploid wheat lines with sequenced genome assemblies: *T. aestivum* varieties CS (Chinese Spring) (IWGSC, 2018), CDC_Stan (CDC Stanley), Claire, Mace, Norin 61, Weebill 1, Arina*LrFor*, Cadenza, CDC_Land (CDC Landmark), Jagger, LongReach (LongReach Lancer), Paragon, Robigus, Julius, SY Matis and the *T. aestivum* ssp. *spelta* accession PI90962 (Walkowiak et al. 2020). Also included are the *WAPO-A1* genomic sequences generated by Sanger sequencing in the eight BMWpop founders (GenBank accessions MW366865 to MW366872). The positions of exon-1 and exon-2 are indicated by the blue and red lines, respectively. The region coding the F-box domain is indicated by the dashed black line. The 21 DNA variants identified in the sequence alignments are numbered, as also summarised in Supplementary Table 8a. Within the coding regions, DNA variants 10 and 15 result in amino acid substitutions F47/C and D384/N in the predicted protein, respectively.
