# Supplementary material for: Identification of eight QTL controlling multiple yield components in a German multi-parental wheat population, including Rht24, WAPO-A1, WAPO-B1 and genetic loci on chromosomes 5A and 6A
Source: Theor Appl Genet. 2021 Mar 12;134(5):1435–54. doi: 10.1007/s00122-021-03781-7 (PMC8081691; doi:10.1007/s00122-021-03781-7)
Supplement: Supplementary file 5 — Supplementary Figure 5. Genomic DNA alignment of WAPO-B1, including 1,000 bp up- and down-stream of the coding regions, from 16 hexaploid wheat lines with sequenced genome assemblies: T. aestivum varieties CS (Chinese Spring) (IWGSC 2018), CDC_Stan (CDC Stanley), Claire, Mace, Norin 61, Weebill 1, ArinaLrFor, Cadenza, CDC_Land (CDC Landmark), Jagger, LongReach (LongReach Lancer), Paragon, Robigus, Julius, SY Matis and the T. aestivum ssp. spelta accession PI90962 (Walkowiak et al. 2020). Also included are the WAPO-B1 genomic sequences generated by Sanger sequencing in seven of the eight BMWpop founders (GenBank accessions MW366873 to MW366879). We were not able to PCR amplify WAPO-B1 from the BMWpop founder Firl3565. The position of the (CT)n microsatellite upstream of the start codon is indicated by the green line. The positions of exon-1 and exon-2 are indicated by the blue and red lines, respectively. The region coding the F-box domain is indicated by the dashed black line. The 17 DNA variants identified in the sequence alignments are numbered, as also summarised in Supplementary Table 8b. Within the coding regions, DNA variants 5 (A+140/G) and 6 (G+427/A) result in amino acid substitutions H47/R and D143/N in the predicted protein, respectively. The 5 bp deletion within the region encoding the F-box domain in exon-1 (variant 4, present in LongReach Lancer, Mace, Weebill 1 and PI90962) results in a subsequent frame shift in the predicted protein, and a premature stop codon (TAA) at the position indicated by the black triangle. *Variant 6 (G+427/A) is present only in WAPO-B1.hap3 (LongReach Lancer, Mace, Weebill 1 and PI90962) and encodes for a glutamine (Q) residue; however, in this haplotype the preceding 5 bp exon-1 deletion means that the amino acid sequence at this point in the predicted protein has already been knocked out of frame. $Variant 7 (G+517/T) results in amino acid change A173/S in WAPO-B1.hap.2, while in WAPO-B1.hap3, where the protein has already [file 122_2021_3781_MOESM5_ESM.docx]

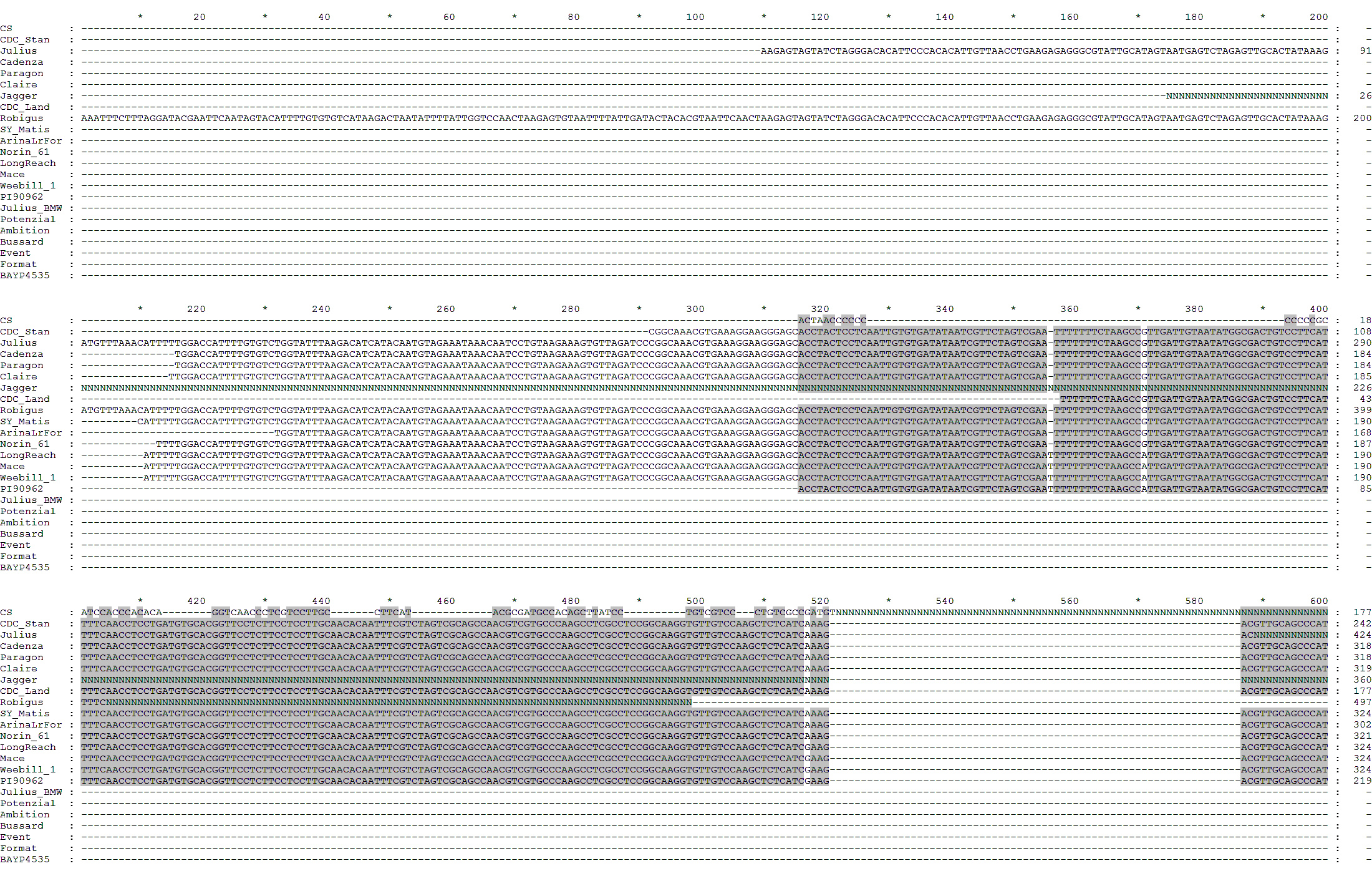


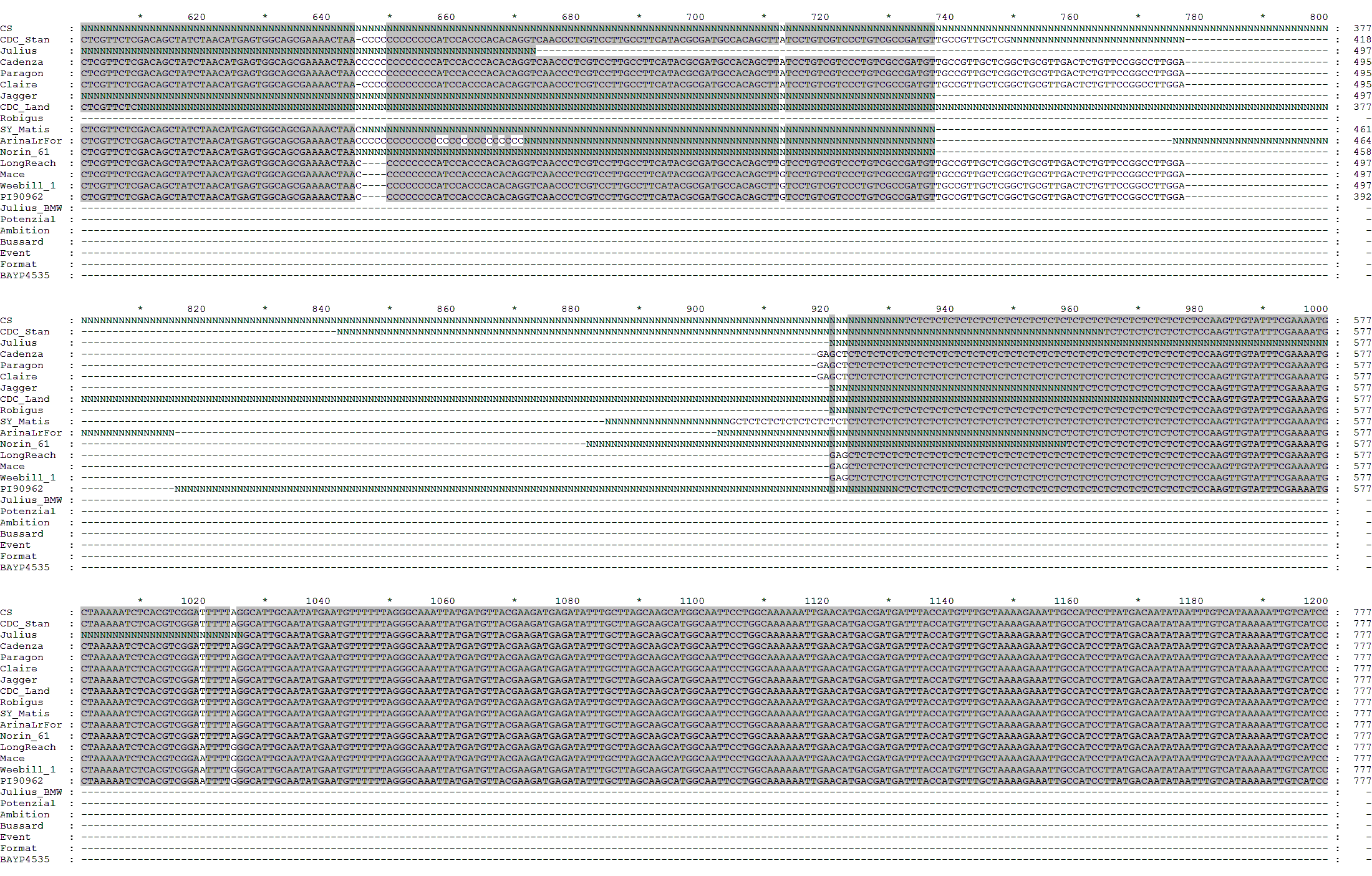


1 2


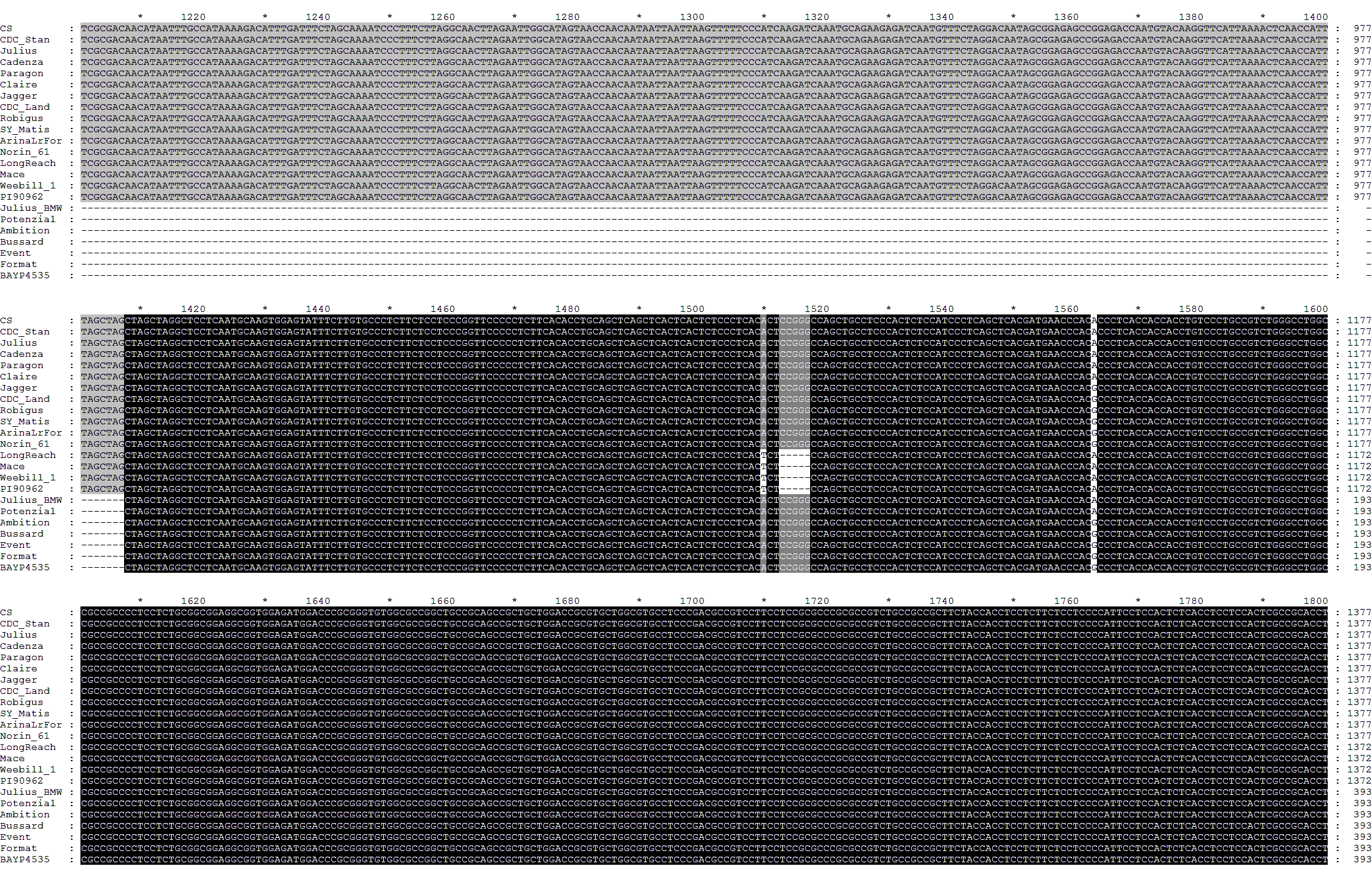


3 4 (🡪 frame shift and stop codon) 5 (A+140/G 🡪 H47/R)


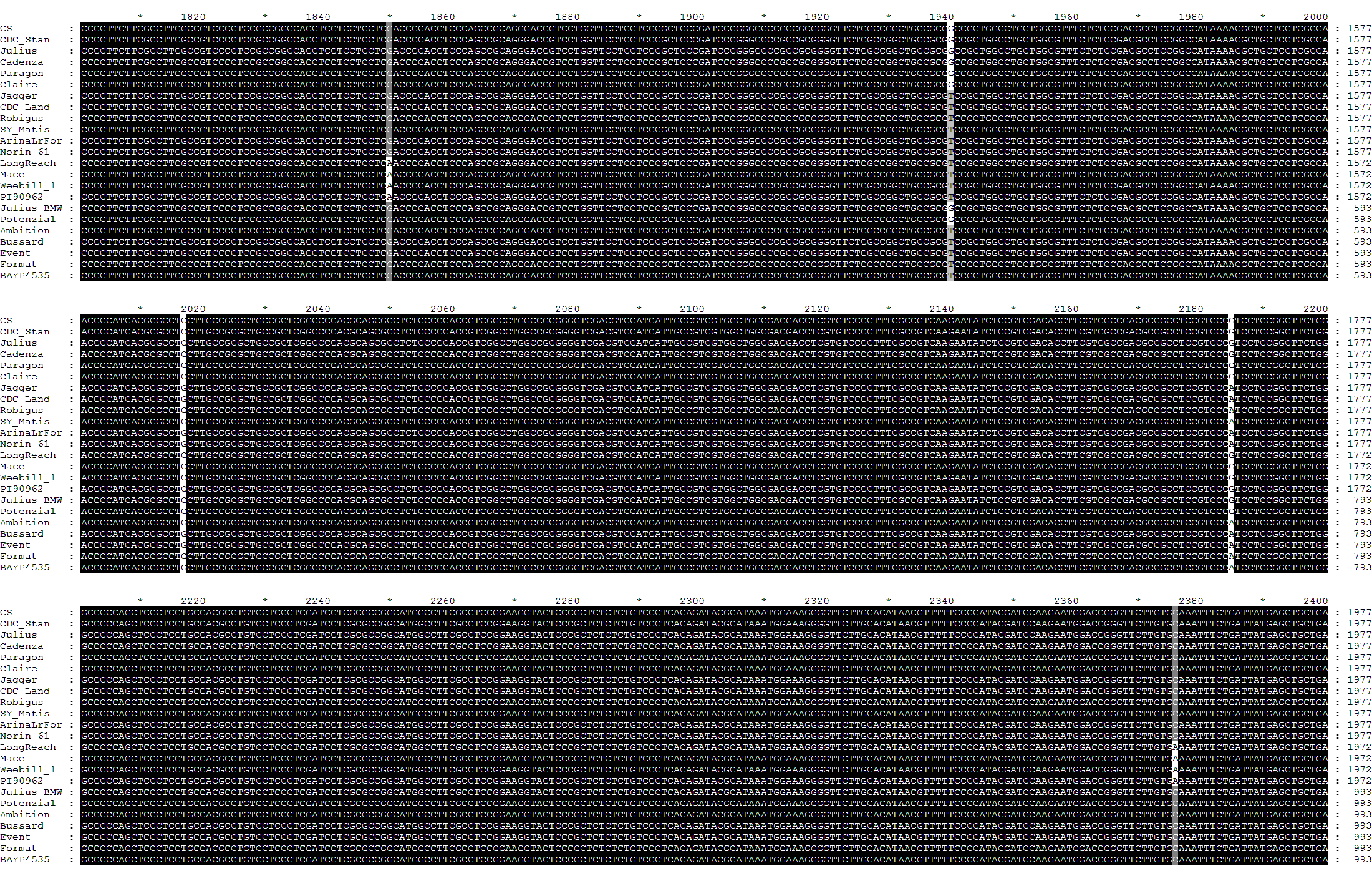


6 (G+427/A^*^) 7 (G+517/T 🡪 A173/S^$^)

8 9

10


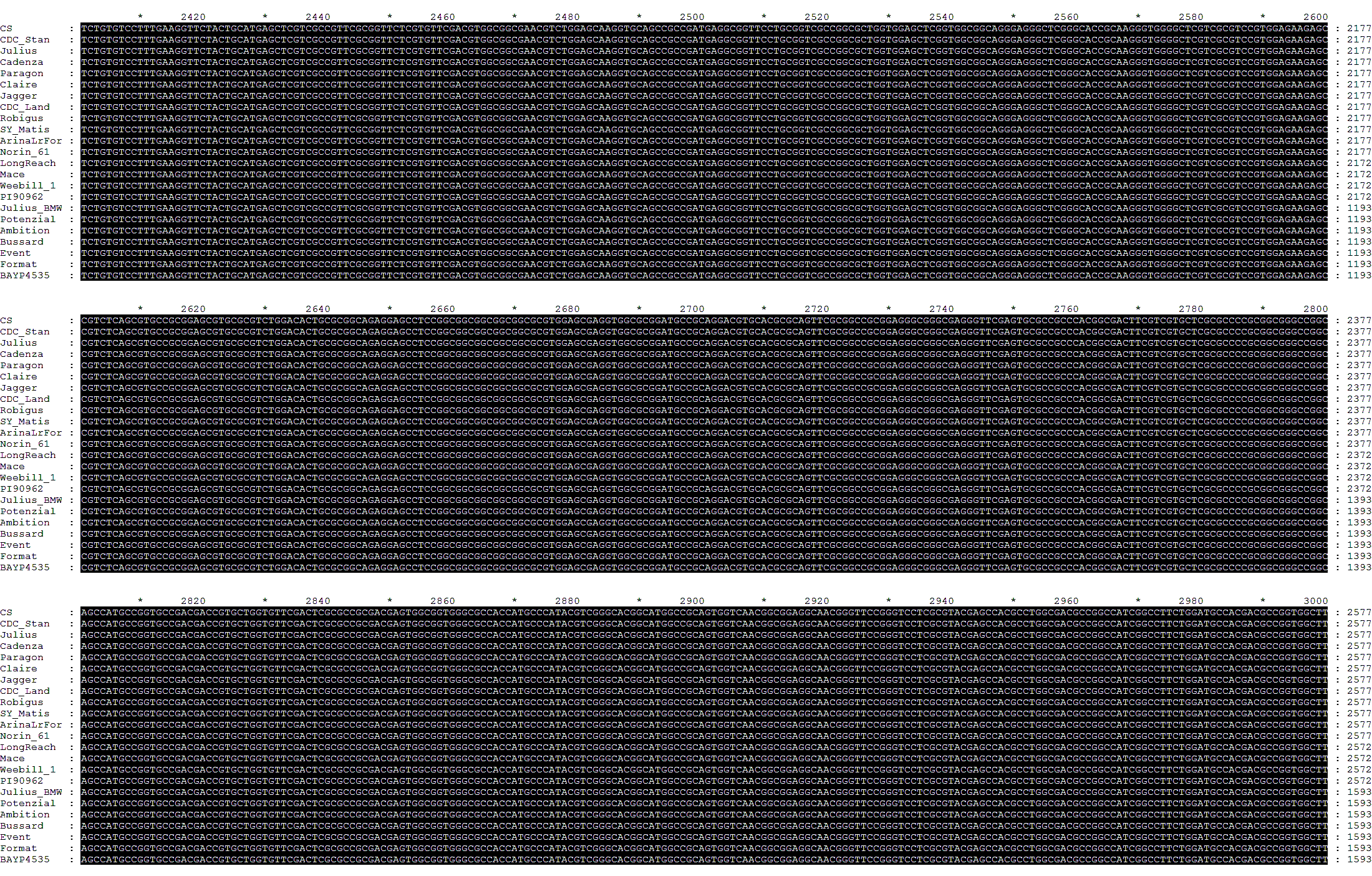


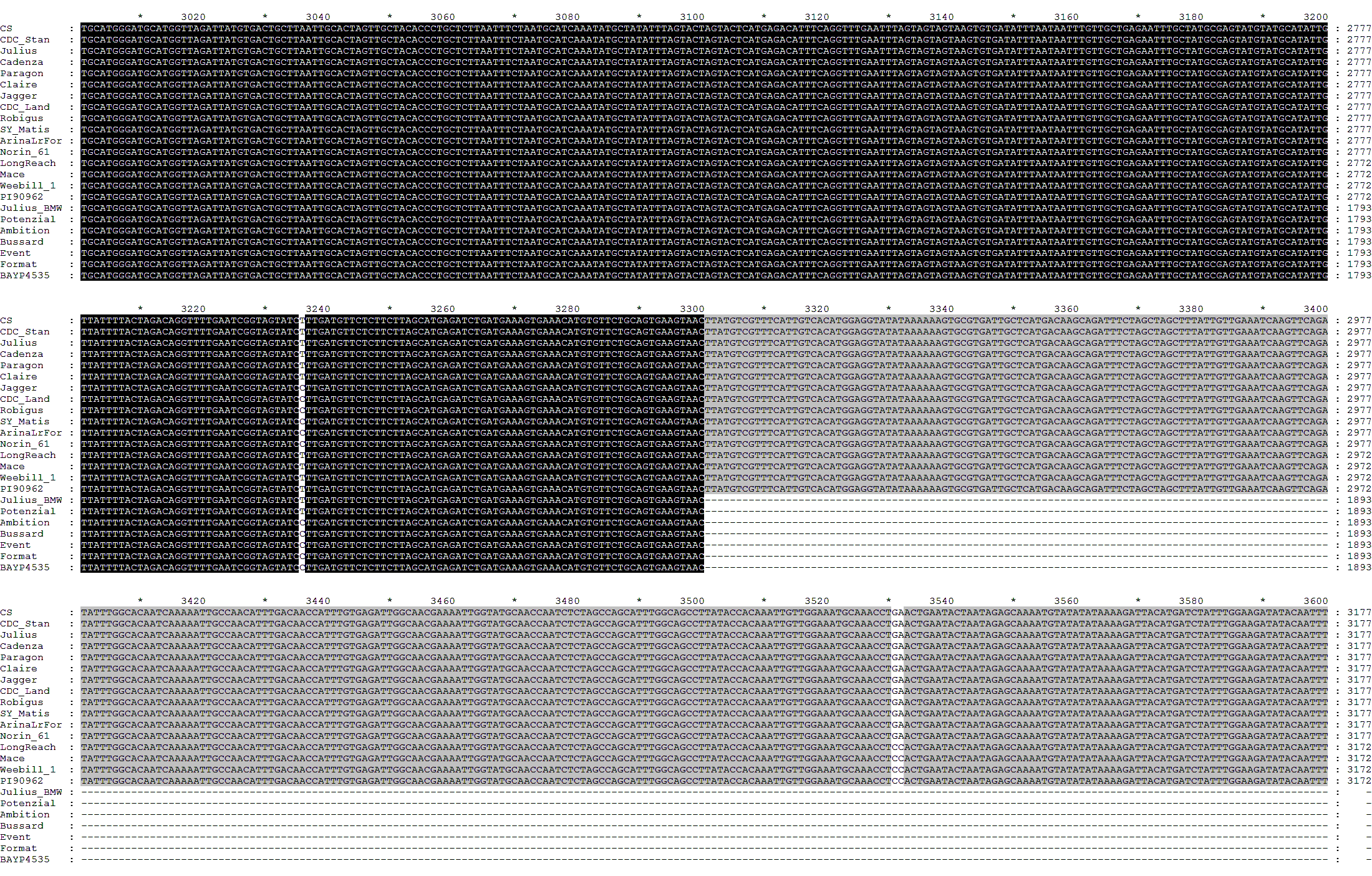


12 13

11


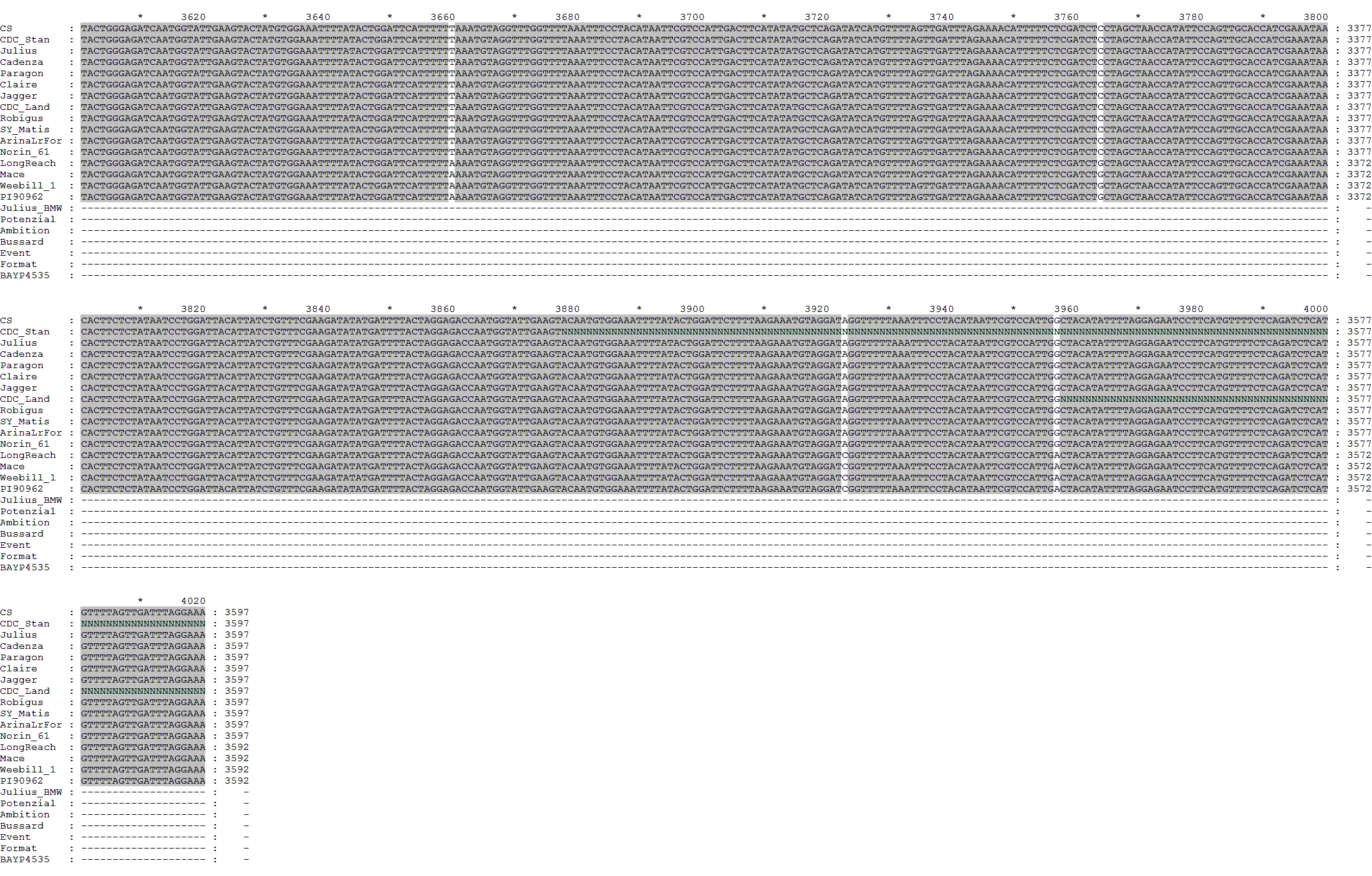


16 17

14 15

**Supplementary Figure 5.** Genomic DNA alignment of *WAPO-B1*, including 1,000 bp up- and down-stream of the coding regions, from 16 hexaploid wheat lines with sequenced genome assemblies: *T. aestivum* varieties CS (Chinese Spring) (IWGSC, 2018), CDC_Stan (CDC Stanley), Claire, Mace, Norin 61, Weebill 1, Arina*LrFor*, Cadenza, CDC_Land (CDC Landmark), Jagger, LongReach (LongReach Lancer), Paragon, Robigus, Julius, SY Matis and the *T. aestivum* ssp. *spelta* accession PI90962 (Walkowiak et al. 2020). Also included are the *WAPO-B1* genomic sequences generated by Sanger sequencing in seven of the eight BMWpop founders (GenBank accessions MW366873 to MW366879). We were not able to PCR amplify *WAPO-B1* from the BMWpop founder Firl3565. The position of the (CT)_n_ microsatellite upstream of the start codon is indicated by the green line. The positions of exon-1 and exon-2 are indicated by the blue and red lines, respectively. The region coding the F-box domain is indicated by the dashed black line. The 17 DNA variants identified in the sequence alignments are numbered, as also summarised in Supplementary Table 8b. Within the coding regions, DNA variants 5 (A+140/G) and 6 (G+427/A) result in amino acid substitutions H47/R and D143/N in the predicted protein, respectively. The 5 bp deletion within the region encoding the F-box domain in exon-1 (variant 4, present in LongReach Lancer, Mace, Weebill 1 and PI90962) results in a subsequent frame shift in the predicted protein, and a premature stop codon (TAA) at the position indicated by the black triangle. ^*^Variant 6 (G+427/A) is present only in *WAPO-B1.hap3* (LongReach Lancer, Mace, Weebill 1 and PI90962) and encodes for a glutamine (Q) residue; however, in this haplotype the preceding 5 bp exon-1 deletion means that the amino acid sequence at this point in the predicted protein has already been knocked out of frame. ^$^Variant 7 (G+517/T) results in amino acid change A173/S in *WAPO-B1.hap.2*, while in *WAPO-B1.*hap3, where the protein has already been knocked out of frame, it results in a valine (V) residue.
