# Supplementary material for: Identification of eight QTL controlling multiple yield components in a German multi-parental wheat population, including Rht24, WAPO-A1, WAPO-B1 and genetic loci on chromosomes 5A and 6A
Source: Theor Appl Genet. 2021 Mar 12;134(5):1435–54. doi: 10.1007/s00122-021-03781-7 (PMC8081691; doi:10.1007/s00122-021-03781-7)
Supplement: Supplementary file 6 — Supplementary Figure 6. Alignment of the predicted proteins identified as ‘orthologous’ to WAPO-B1 in the Ensembl Plants database. Details of the 56 proteins sourced from 53 species are listed in Supplementary Table 4. Where more than one protein sequence was identified in a given species, only the first was used here, except for polyploid Triticum species where all sequences identified as ‘orthologues’ were included. The locations of the F-box domain and the two amino acid substitutions resulting from DNA variants identified in our WAPO-B1 haplotype analysis (detailed in Supplementary Table 8b) are indicated. Species abbreviations: Ach (Actinidia chinensis), Aet (Aegilops tauschii), Atr (Amborella trichopoda), Aco (Ananas comosus), Ath (Arabidopsis thaliana), Aap (Arabis alpine), Bvu (Beta vulgaris), Bdi (Brachypodium distachyon), Bna (Brassica napus), Bol (Brassica oloracea), Bra (Brassica rapa), Camsa (Camelina sativa), Cansa (Cannabis sativa), Can (Caspicum annum), Cla (Citrullus lanatus), Ccl (Citrus clementina), Cofca (Coffea canephora), Ccap (Corchorus capsularis), Cme (Cucumis melo), Csa (Cucumis sativus), Cca (Cynara cardunculus), Dca (Daucus carota), Dro (Dioscorea rotundata), Gma (Glycine max), Han (Helianthus annuus), Hvu (Hordeum vulgare), Itr (Ipomoea triloba), Lan (Lupinus angustifolius), Mdo (Malus domestica), Mes (Manihot esculenta), Mtr (Medicago truncatula), Nat (Nicotiana attenuate), Oer (Olea europaea), Osa (Oryza sativa), Pvu (Phaseolus vulgaris), Pve (Pistacia vera), Ptr (Populus trichocarpa), Pav (Prunus avium), Ppe (Prunus persica), Pu (Prunus dulcis), Rch (Rosa chinensis), Smo (Selaginella moellendorffii), Sly (Solanum lycopersicum), Stu (Solanum tuberosum), Tca (Theobroma cacao), Tpr (Trifolium pratense), Tae (Triticum aestivum; A-, B- and D-genome homoeologue indicated), Tdi (Triticum dicoccoides; A- and B-genome homoeologue indicated), Ttu (Triticum turgidum; B-genome homoeologue indicated), Van (Vigna angularis), Vra (Vigna radiata), Vv [file 122_2021_3781_MOESM6_ESM.docx]

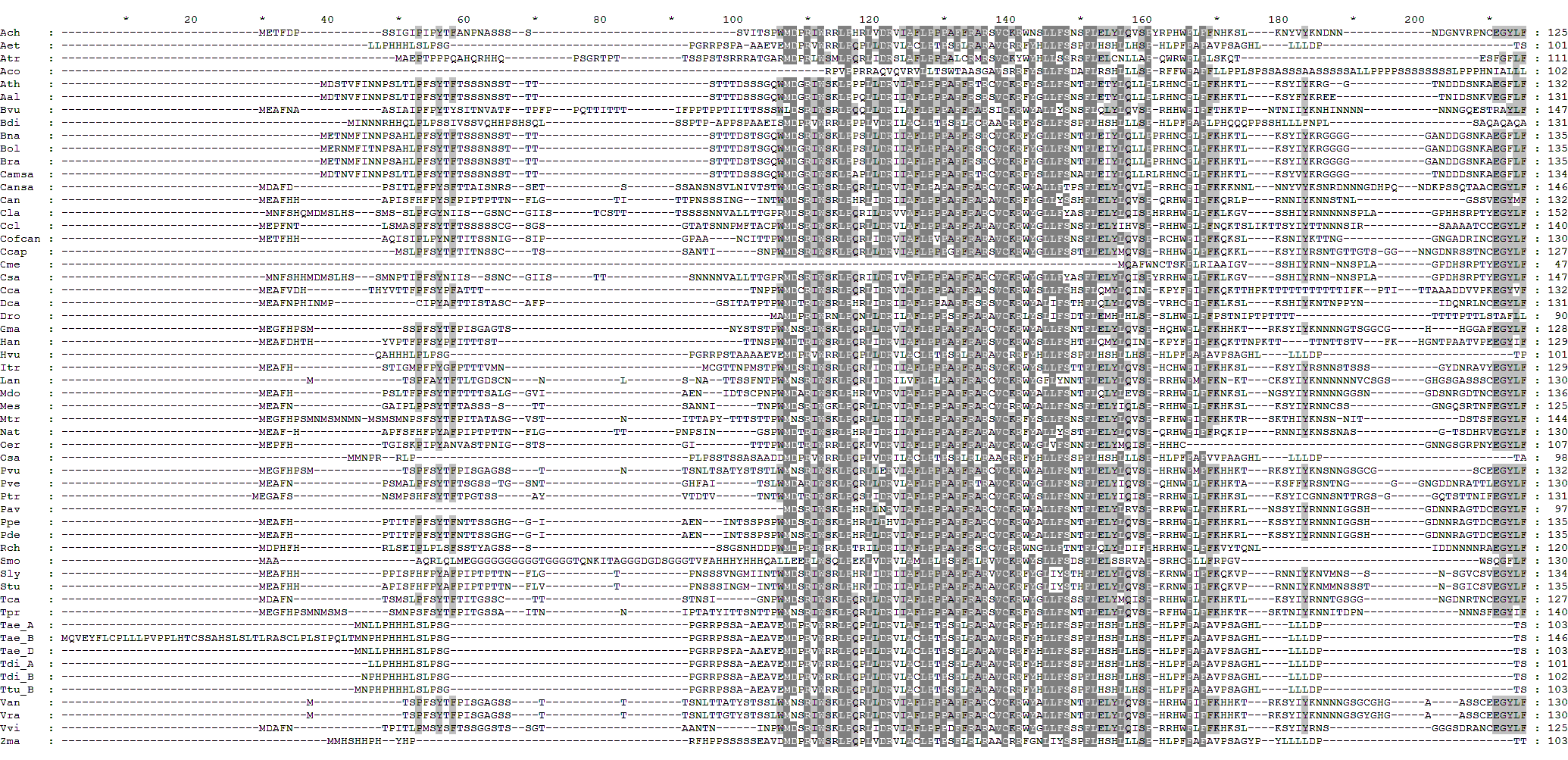


F-box domain

WAPO-B1 H47/R amin acid substitution present in low spikelet number alleles in the BMWpop founders Ambition, Bussard, Event, Format and BAYP4535


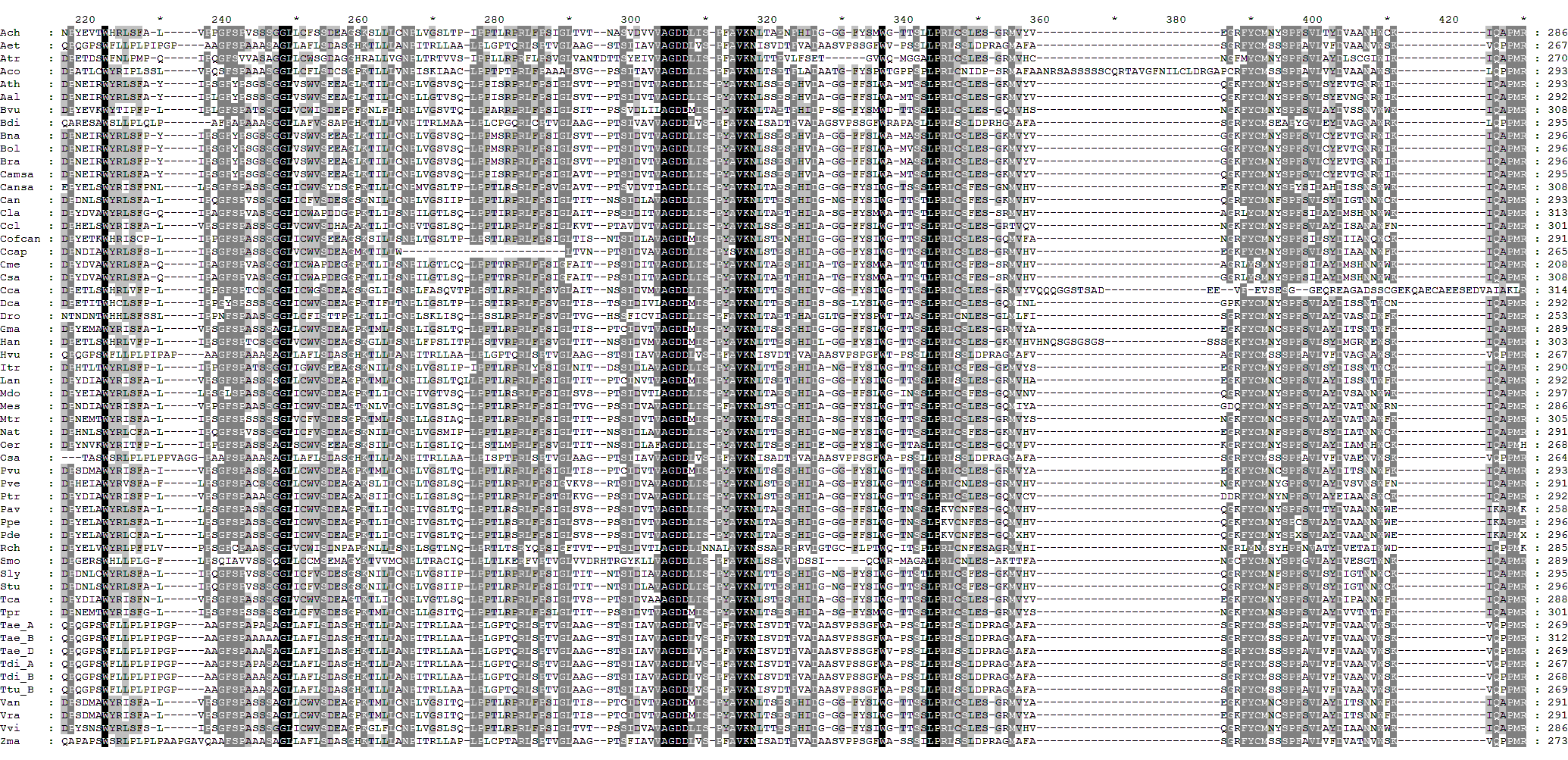


WAPO-B1 A173/S amio acid substitution present in low spikelet number alleles in the BMWpop founders Ambition, Bussard, Event, Format and BAYP4535


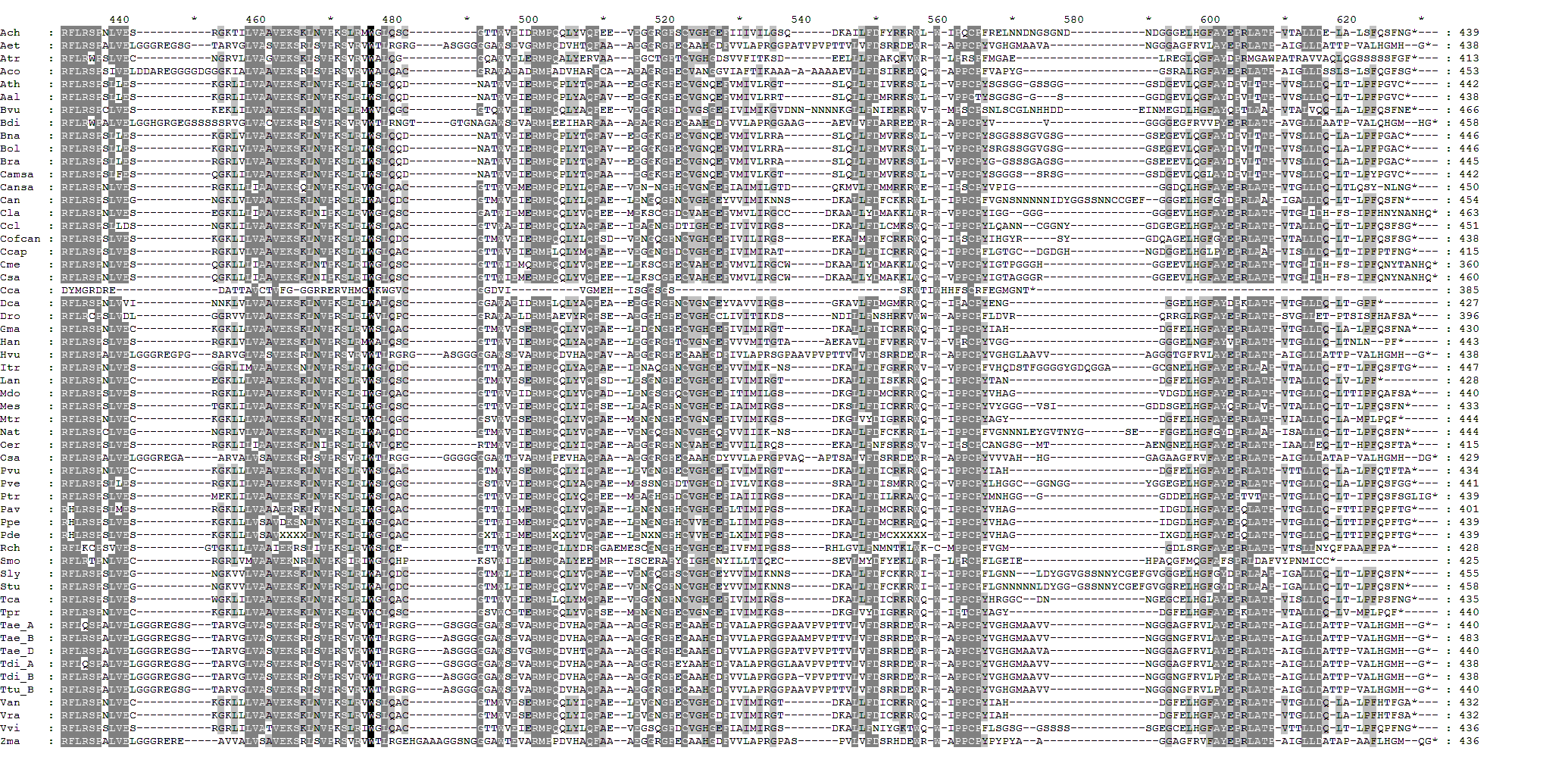


**Supplementary Figure 6.** Alignment of the predicted proteins identified as ‘orthologous’ to WAPO-B1 in the Ensembl Plants database. Details of the 56 proteins sourced from 53 species are listed in Supplementary Table 3. Where more than one protein sequence was identified in a given species, only the first was used here, except for polyploid *Triticum* species where all sequences identified as ‘orthologues’ were included. The locations of the F-box domain and the two amino acid substitutions resulting from DNA variants identified in our *WAPO-B1* haplotype analysis (detailed in Supplementary Table 8b) are indicated. Species abbreviations: Ach (*Actinidia chinensis*), Aet (*Aegilops tauschii*), Atr (*Amborella trichopoda*), Aco (*Ananas comosus*), Ath (*Arabidopsis thaliana*), Aap (*Arabis alpine*), Bvu (*Beta vulgaris*), Bdi (*Brachypodium distachyon*), Bna (*Brassica napus*), Bol (*Brassica oloracea*), Bra (*Brassica rapa*), Camsa (*Camelina sativa*), Cansa (*Cannabis sativa*), Can (*Caspicum annum*), Cla (*Citrullus lanatus*), Ccl (*Citrus clementina*), Cofca (*Coffea canephora*), Ccap (*Corchorus capsularis*), Cme (*Cucumis melo*), Csa (*Cucumis sativus*), Cca (*Cynara cardunculus*), Dca (*Daucus carota*), Dro (*Dioscorea rotundata*), Gma (*Glycine max*), Han (*Helianthus annuus*), Hvu (*Hordeum vulgare*), Itr (*Ipomoea triloba*), Lan (*Lupinus angustifolius*), Mdo (*Malus domestica*), Mes (*Manihot esculenta*), Mtr (*Medicago truncatula*), Nat (*Nicotiana attenuate*), Oer (*Olea europaea*), Osa (*Oryza sativa*), Pvu (*Phaseolus vulgaris*), Pve (*Pistacia vera*), Ptr (*Populus trichocarpa*), Pav (*Prunus avium*), Ppe (*Prunus persica*), Pu (*Prunus dulcis*), Rch (*Rosa chinensis*), Smo (*Selaginella moellendorffii*), Sly (*Solanum lycopersicum*), Stu (*Solanum tuberosum*), Tca (*Theobroma cacao*), Tpr (*Trifolium pratense*), Tae (*Triticum aestivum*; A-, B- and D-genome homoeologue indicated), Tdi (*Triticum dicoccoides*; A- and B-genome homoeologue indicated), Ttu (*Triticum turgidum*; B-genome homoeologue indicated), Van (*Vigna angularis*), Vra (*Vigna radiata*), Vvi (*Vitis vinifera*), Zma (*Zea mays*).
