# Supplementary material for: Identification of eight QTL controlling multiple yield components in a German multi-parental wheat population, including Rht24, WAPO-A1, WAPO-B1 and genetic loci on chromosomes 5A and 6A
Source: Theor Appl Genet. 2021 Mar 12;134(5):1435–54. doi: 10.1007/s00122-021-03781-7 (PMC8081691; doi:10.1007/s00122-021-03781-7)
Supplement: Supplementary file 7 — Supplementary Figure 7. Heatmap of WAPO-A1, -B1 and -D1 gene expression in different wheat tissues at different developmental stages. Data was sourced from the wheat gene expression atlas (Ramírez-González et al. 2018). TPM = transcripts per million. (DOCX 257 kb) [file 122_2021_3781_MOESM7_ESM.docx]

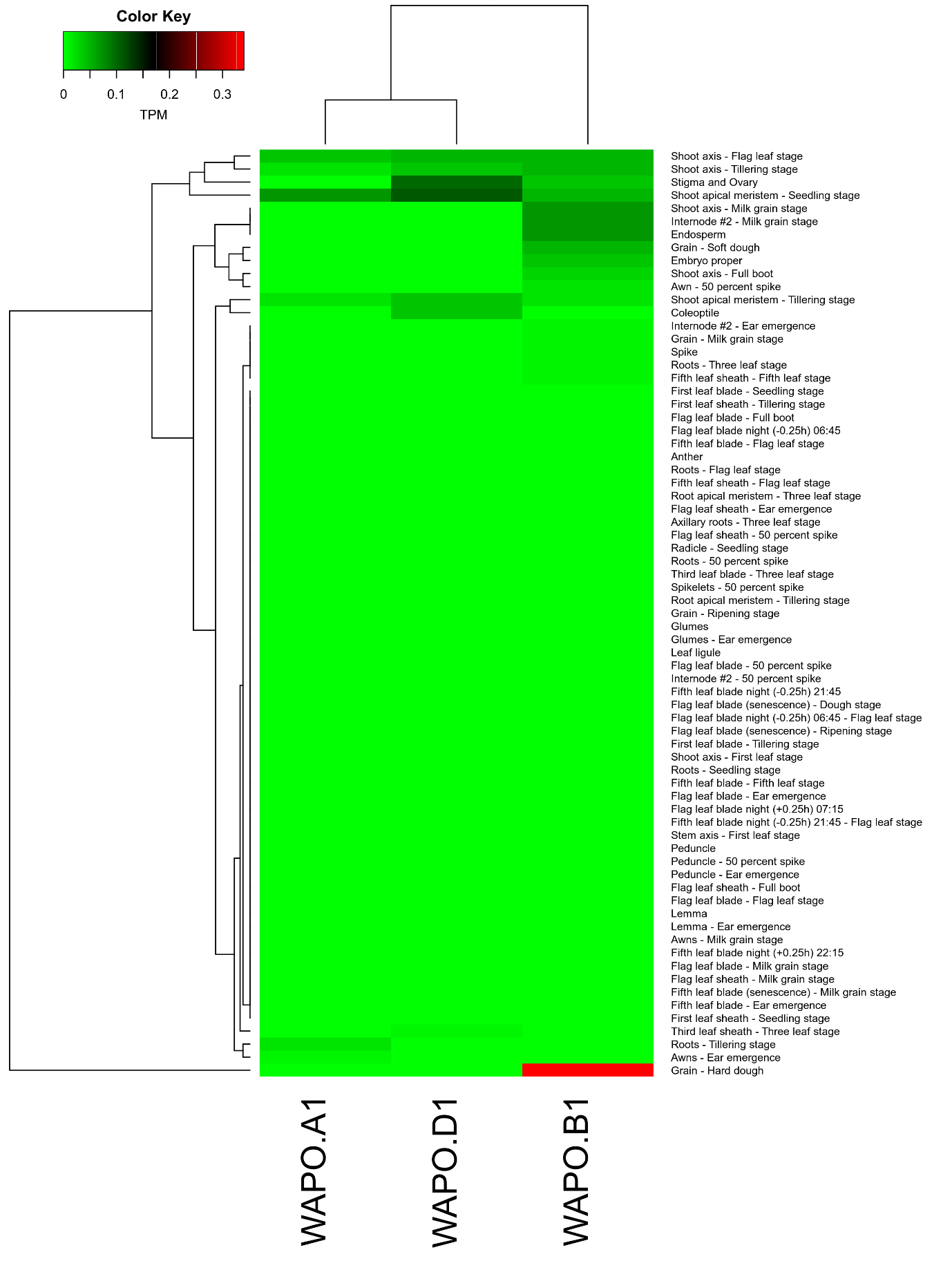


**Supplementary Figure 7.** Heatmap of *WAPO-A1, -B1* and *-D1* gene expression in different wheat tissues at different developmental stages. Data was sourced from the wheat gene expression atlas (Ramírez-González et al. 2018). TPM = transcripts per million.
