# Supplementary material for: Identification of eight QTL controlling multiple yield components in a German multi-parental wheat population, including Rht24, WAPO-A1, WAPO-B1 and genetic loci on chromosomes 5A and 6A
Source: Theor Appl Genet. 2021 Mar 12;134(5):1435–54. doi: 10.1007/s00122-021-03781-7 (PMC8081691; doi:10.1007/s00122-021-03781-7)
Supplement: Supplementary file 8 — Supplementary Table 1. Disease scoring guide used to assess yellow rust (YR) and septoria tritici blotch (STB) infection on a percentage scale in the BMWpop. Assessment was at the plot level and based on disease present in the top four leaves. (DOCX 14 kb) [file 122_2021_3781_MOESM8_ESM.docx]

| **% infection** | **YR** | **STB** |
| --- | --- | --- |
| 0 | No infection observed | No infection observed |
| 0.1 | One stripe per tiller | One lesion per 10 tillers |
| 1 | Two stripes per leaf | Two small lesions per tiller |
| 5 | Most tillers infected but some tops leaves uninfected | Small lesions beginning to form areas of dead tissue across width of leaf |
| 10 | All leaves infected but leaves appear green overall | Two lower leaves- large areas of diseased tissue, some covering a third of the leaf |
| 25 | Leaves appear half infected, half green | |
| 50 | Leaves appear more infected than green | |
| 75 | Very little green leaf tissue left | |
| 100 | Leaves dead (no green tissue left) | |

**Supplementary Table 1.** Disease scoring guide used to assess yellow rust (YR) and septoria tritici blotch (STB) infection on a percentage scale in the BMWpop. Assessment was at the plot level and based on disease present in the top four leaves.
