# Supplementary material for: Identification of eight QTL controlling multiple yield components in a German multi-parental wheat population, including Rht24, WAPO-A1, WAPO-B1 and genetic loci on chromosomes 5A and 6A
Source: Theor Appl Genet. 2021 Mar 12;134(5):1435–54. doi: 10.1007/s00122-021-03781-7 (PMC8081691; doi:10.1007/s00122-021-03781-7)
Supplement: Supplementary file 9 — Supplementary Table 2. Trait correlations. (A) Trait correlations between trials conducted in the United Kingdom in 2017 (UK17), in the UK in 2018 (UK18) and in Germany in 2018 (DE18), and (B) their corresponding significance values. P-values ≤ 0.05 are indicated in bold. Trait abbreviations: EL (ear length), EW (ear width), NFSP (number of fertile spikelets per ear), NISP (number of infertile spikelets per ear), totNSP (total number of spikelets per ear), NS.NE (number of seeds per ear), WS.EW (seed weight/ear weight ratio), SA (seed area), SWI (seed width), SL (seed length), FFD (factor form density), SL.SWI (seed length/seed width ratio), FT (flowering time), HT (plant height), TGW (thousand grain weight). (XLSX 49 kb) [file 122_2021_3781_MOESM9_ESM.docx]

| **Target gene** | **Primer name** | **Sequence 5’ to 3’** | **Information** |
| --- | --- | --- | --- |
| *WAPO-A1* | WAPOA1_2F | GCTAGCTAGGCTCCTCAA | PCR *WAPO-A1* and sequencing |
| *WAPO-A1* | WAPOA1_4R | ATGTGATCATGAAACGACG |  |
| *WAPO-B1* | WAPOB1_49F_UFO | CTAGGACAATAGCGGAGAGCC | PCR *WAPO-B1* and sequencing |
| *WAPO-B1* | WAPOB1_49R_UFO | CTTGTCATGAGCAATCACG |  |
| *WAPO-A1* | WAPOA1_3F | CACCTCCTCCTCCTCGAT | Sequencing |
| *WAPO-A1* | WAPOA1_4F | CATAAATGGAAAGGGGTTCA | Sequencing |
| *WAPO-A1/-B1* | UFOABD_F1 | GGCGGTGGAGATGGACCC | Sequencing |
| *WAPO-B1* | WAPOB1_4F | GGGTCGACGTCCATCATT | Sequencing and PCR *WAPO-B1* with WAPOB1_49R_UFO |
| *WAPO-B1* | WAPOB1_3R | GAAGGTGTCGACGGAGATA | Sequencing and PCR *WAPO-B1* with WAPOB1_2F |
| *WAPO-B1* | UFOABD_R2 | CACGCTGAGACGGCTCTTC | Sequencing |
| *WAPO-A1/-B1* | WAPOAB_JCR1 | ATGCATCCCATGCAAAGCCA | Sequencing |
| *WAPO-B1* | WAPOB1_2F | GCTAGCTAGGCTCCTCAA | PCR *WAPO-B1* in Firl3565 with WAPOB1_3R |

**Supplementary Table 2.** Primers used to PCR amplify and Sanger sequence *WAPO-A1* and *WAPO-B1* in the BMWpop founders. Three primer pairs were used to attempt to amplify *WAPO-B1* in the founder Firl3565: WAPOB1_49F_UFO/WAPOB1_49R_UFO, WAPOB1_2F/WAPOB1_3R and WAPOB1_4F/WAPOB1_49R_UFO. PCR was undertaken using the following conditions: 9 min at 96 °C followed by 36 cycles of 96 °C for 45 secs, 58 °C for 45 secs and 72 °C for 120 secs, with a final extension stage of 72 °C for 7 mins.
