# Supplementary material for: Identification of eight QTL controlling multiple yield components in a German multi-parental wheat population, including Rht24, WAPO-A1, WAPO-B1 and genetic loci on chromosomes 5A and 6A
Source: Theor Appl Genet. 2021 Mar 12;134(5):1435–54. doi: 10.1007/s00122-021-03781-7 (PMC8081691; doi:10.1007/s00122-021-03781-7)
Supplement: Supplementary file 10 — Supplementary Table 3. Primers used to PCR amplify and Sanger sequence WAPO-A1 and WAPO-B1 in the BMWpop founders. Three primer pairs were used to attempt to amplify WAPO-B1 in the founder Firl3565: WAPOB1_49F_UFO/WAPOB1_49R_UFO, WAPOB1_2F/WAPOB1_3R and WAPOB1_4F/WAPOB1_49R_UFO. PCR was undertaken using the following conditions: 9 min at 96 °C followed by 36 cycles of 96 °C for 45 secs, 58 °C for 45 secs and 72 °C for 120 secs, with a final extension stage of 72 °C for 7 mins. (DOCX 15 kb) [file 122_2021_3781_MOESM10_ESM.docx]

|  | **Species** | **Protein accession** | **Used in WAPO-A1 alignments** | **Used in WAPO-B1 alignments** | **Grass sp. ?** |
| --- | --- | --- | --- | --- | --- |
| 1 | *Actinidia chinensis* | CEY00_Acc20814 | Y | Y | No |
| 2 | *Aegilops tauschii* | AET7Gv21179300 | Y | Y | Yes |
| 3 | *Amborella trichopoda* | AMTR_s00032p00208090 | Y | Y | No |
| 4 | *Ananas comosus* | Aco008339.1.path1 | Y | Y | No |
| 5 | *Arabidopsis thaliana* | AT1G30950 | Y | Y | No |
| 6 | *Arabis alpine* | KFK44889 | N | Y | No |
| 7 | *Beta vulgaris* | BVRB_4g086600 | Y | Y | No |
| 8 | *Brachypodium distachyon* | BRADI_1g31880v3 | Y | Y | Yes |
| 9 | *Brassica napus* | BnaAnng11240D | Y | Y | No |
| 10 | *Brassica oloracea* | Bo2g121010 | Y | Y | No |
| 11 | *Brassica rapa* | Bra039878 | Y | Y | No |
| 12 | *Camelina sativa* | Csa03g034360 | Y | Y | No |
| 13 | *Cannabis sativa* | evm.TU.09.1002 | Y | Y | No |
| 14 | *Caspicum annum* | T459_06031 | Y | Y | No |
| 15 | *Citrullus lanatus* | Cla97C03G055340 | Y | Y | No |
| 16 | *Citrus clementina* | CICLE_v10017846mg | Y | Y | No |
| 17 | *Coffea canephora* | GSCOC_T00007092001 | Y | Y | No |
| 18 | *Corchorus capsularis* | CCACVL1_13194 | Y | Y | No |
| 19 | *Cucumis melo* | Csa_1G056950 | N | Y | No |
| 20 | *Cucumis sativus* | KGN64459 | Y | Y | No |
| 21 | *Cynara cardunculus* | Ccrd_024829 | Y^†^ | Y | No |
| 22 | *Daucus carota* | DDCAR_001274 | Y | Y | No |
| 23 | *Dioscorea rotundata* | DRNTG_13981 | Y | Y | No |
| 24 | *Glycine max* | GLYMA_05G134000 | Y | Y | No |
| 25 | *Gossypium raimondii* | B456_002G139700 | Y | N | No |
| 26 | *Helianthus annuus* | HannXRQ_Chr09g0257841 | Y | Y | No |
| 27 | *Hordeum vulgare* | HORVU7Hr1G108970 | Y | Y | Yes |
| 28 | *Ipomoea triloba* | itb10g12520 | Y | Y | No |
| 29 | *Leersia perrieri* | LPERR06G19890 | Y | N | No |
| 30 | *Lupinus angustifolius* | TanjilG_19292 | Y | Y | No |
| 31 | *Malus domestica* | MD05G0207000 | Y | Y | No |
| 32 | *Manihot esculenta* | MANES_13G074800 | Y | Y | No |
| 33 | *Medicago truncatula* | MTR_4g094748 | Y | Y | No |
| 34 | *Musa acuminata* | GSMUA_Achr3G23630_001 | Y | N | No |
| 35 | *Nicotiana attenuate* | UFO_0 (A4A49_42330) | Y | Y | No |
| 36 | *Olea europaea* | Oeu056060 | Y | Y | No |
| 37 | *Oryza sativa Japonica* | APO1 (Os06g0665400) | Y | Y | Yes |
| 38 | *Phaseolus vulgaris* | PHAVU_002G188300g | Y | Y | No |
| 39 | *Physcomitrella patens* | Pp3c11_18710 | Y | N | No |
| 40 | *Pistacia vera* | EVM0013296 | Y | Y | No |
| 41 | *Populus trichocarpa* | POPTR_001G160900v3 | Y | Y | No |
| 42 | *Prunus avium* | Pav_sc0001465.1_g150.1.mk | Y | Y | No |
| 43 | *Prunus dulcis* | Prudul26B013786 | Y | Y | No |
| 44 | *Prunus persica* | PRUPE_4G117600 | Y | Y | No |
| 45 | *Rosa chinensis* | RchiOBHm_Chr3g0456371 | Y | Y | No |
| 46 | *Selaginella moellendorffii* | SELMODRAFT_407049 | Y | Y | No |
| 47 | *Solanum lycopersicum* | Solyc02g081670.1 | Y | Y | No |
| 48 | *Solanum tuberosum* | PGSC0003DMG400017736 | Y | Y | No |
| 49 | *Theobroma cacao* | Tc02v2_g000340 | Y | Y | No |
| 50 | *Trifolium pratense* | Tp57577_TGAC_v2_gene23831 | Y | Y | No |
| 51 | *Triticum aestivum** | TraesCS7A02G481600 | Y | Y | Yes |
| 52 | *Triticum aestivum** | TraesCS7B02G384000 | Y | Y | Yes |
| 53 | *Triticum aestivum** | TraesCS7D02G468700 | Y | Y | Yes |
| 54 | *Triticum dicoccoides** | TRIDC7AG067090 | Y | Y | Yes |
| 55 | *Triticum dicoccoides** | TRIDC7BG060970 | Y | Y | Yes |
| 56 | *Triticum turgidum* | TRITD7Bv1G202770 | Y | Y | Yes |
| 57 | *Vigna angularis* | LR48_Vigan107s001700 | Y | Y | No |
| 58 | *Vigna radiata* | Vradi07g09760 | Y | Y | No |
| 59 | *Vitis vinifera* | VIT_01s0011g01220 | Y | Y | No |
| 60 | *Zea mays* | Zm00001d036653_T001 | N | Y | Yes |

**Supplementary Table 3.** Protein sequences from plant species with sequenced genomes identified as ‘orthologues’ of WAPO-A1/-B1 in Ensembl Plants, and used for sequence alignments and analysis of conserved domains. Proteins were excluded if they had deletions within the investigated regions, or if they had sequence similarity with WAPO-A1/-B1 of <20%. Where more than one protein sequence was identified in a given species, only the most similar to WAPO-A1/-B1 was used here (^*^except for polyploid *Triticum* species, where all sequences identified as ‘orthologues’ were included). ^†^Not used in the Weblogo for the WAPO-A1 D384/N amino acid substitution, due to misalignments within the analysed region.
