# Supplementary material for: Identification of eight QTL controlling multiple yield components in a German multi-parental wheat population, including Rht24, WAPO-A1, WAPO-B1 and genetic loci on chromosomes 5A and 6A
Source: Theor Appl Genet. 2021 Mar 12;134(5):1435–54. doi: 10.1007/s00122-021-03781-7 (PMC8081691; doi:10.1007/s00122-021-03781-7)
Supplement: Supplementary file 14 — Supplementary Table 7. Summary information for the alleles present in the BMWpop founders at Rht-B1 and Rht-D1, and the haplotypes at WAPO-A1 and WAPO-B1. Alleles at the Rht-B1 and Rht-D1 genes are based on the diagnostic genetic markers TG0010 and TG0011, as genotyped by Stadlmeier et al. (2018). Their effect on phenotype are indicated in brackets. Haplotypes at the WAPO-A1 and WAPO-B1 genes that are located within the intervals of BMWpop Meta-QTL QMtqtl.lfl-7A.1 (traits: ‘number of infertile spikelets’, ‘number of fertile spikelets’, ‘total number of spikelets’ and ‘ear length’) and QMtqtl.lfl-7B.1 (traits: ‘number of fertile spikelets’ and ‘total number of spikelets’), respectively, are listed. Their haplotypes were determined as detailed in Supplementary Table 8. Allelic effect of these genetic loci on spikelet number related traits are indicated in brackets as ‘high’ or ‘low’, based on the predicted allelic effects at the QTL summarised in Supplementary Table 6. *As we were unable to amplify WAPO-B1 from founder Firl3565, its WAPO-B1 haplotype is currently unconfirmed. (DOCX 16 kb) [file 122_2021_3781_MOESM14_ESM.docx]

| **Founder** | ***Rht-B1 allele*** | ***Rht-D1 allele*** | ***WAPO-A1 haplotype*** | ***WAPO-B1 haplotype*** |
| --- | --- | --- | --- | --- |
| Ambition | *Rht-B1a* (tall) | *Rht-D1b* (semi dwarf) | *WAPA-A1.hap1* (low) | *WAPO-B1a* (low) |
| Firl3565 | *Rht-B1a* (tall) | *Rht-D1a* (tall) | *WAPA-A1.hap1* (low) | *WAPO-B1b* (high)^*^ |
| Bussard | *Rht-B1a* (tall) | *Rht-D1a* (tall) | *WAPA-A1.hap2* (high) | *WAPO-B1a* (low) |
| Event | *Rht-B1a* (tall) | *Rht-D1b* (semi dwarf) | *WAPA-A1.hap2* (high) | *WAPO-B1a* (low) |
| Format | *Rht-B1a* (tall) | *Rht-D1a* (tall) | *WAPO-A1.hap2* (high) | *WAPO-B1a* (low) |
| Julius | *Rht-B1a* (tall) | *Rht-D1b* (semi dwarf) | *WAPA-A1.hap1* (low) | *WAPO-B1b* (high) |
| Potenzial | *Rht-B1a* (tall) | *Rht-D1b* (semi dwarf) | *WAPA-A1.hap2* (high) | *WAPO-B1b* (high) |
| BAYP4535 | *Rht-B1b* (semi dwarf) | *Rht-D1a* (tall) | *WAPA-A1.hap1* (low) | *WAPO-B1a* (low) |

**Supplementary Table 7.** Summary information for the alleles present in the BMWpop founders at *Rht-B1* and *Rht-D1*, and the haplotypes at *WAPO-A1* and *WAPO-B1*. Alleles at the *Rht-B1* and *Rht-D1* genes are based on the diagnostic genetic markers *TG0010* and *TG0011*, as genotyped by Stadlmeier et al. (2018)*.* Their effect on phenotype are indicated in brackets. Haplotypes at the *WAPO-A1* and *WAPO-B1* genes that are located within the intervals of BMWpop Meta-QTL *QMtqtl.lfl-7A.1* (traits: ‘number of infertile spikelets’, ‘number of fertile spikelets’, ‘total number of spikelets’ and ‘ear length’) and *QMtqtl.lfl-7B.1* (traits: ‘number of fertile spikelets’ and ‘total number of spikelets’), respectively, are listed. Their haplotypes were determined as detailed in Supplementary Table 8. Allelic effect of these genetic loci on spikelet number related traits are indicated in brackets as ‘high’ or ‘low’, based on the predicted allelic effects at the QTL summarised in Supplementary Table 6. ^*^As we were unable to amplify *WAPO-B1* from founder Firl3565, its *WAPO-B1* haplotype is currently unconfirmed.
