# Supplementary material for: Identification of eight QTL controlling multiple yield components in a German multi-parental wheat population, including Rht24, WAPO-A1, WAPO-B1 and genetic loci on chromosomes 5A and 6A
Source: Theor Appl Genet. 2021 Mar 12;134(5):1435–54. doi: 10.1007/s00122-021-03781-7 (PMC8081691; doi:10.1007/s00122-021-03781-7)
Supplement: Supplementary file 16 — Supplementary Notes 1 (DOCX 17 kb) [file 122_2021_3781_MOESM16_ESM.docx]

Raw wheat panicle development RNA-seq reads from BioProject PRJNA325489 SRR3659891–SRR3659902 (Li et al. 2018) were obtained from the National Center for Biotechnology Information (NCBI) Short Read Archive (SRA) using fastq-dump v2.8.1. The reads were trimmed to remove adapters and quality filtered using TrimGalore v0.5.0 (http://www.bioinformatics.babraham.ac.uk/projects/trim_galore/) using command line options: “-q 20 --clip_R1 1 --clip_R2 1 --length 50 –paired”. High and low confidence gene predictions of the IWGSC wheat reference genome assembly RefSeq v1.1 (IWGSC, 2018) were concatenated before indexing with Kallisto v0.46.1 (Bray et al. 2016). kallisto quant was then used to calculate transcripts per million (TPM) values using the command option -b 101. Median TPM values for *WAPO-A1*, -*B1* and -*D1* gene models were obtained from the bootstrapping results using kalliso h5dump and a bespoke python script, before mean averages were calculated for each sample from their duplicates. Tissue-specific gene expression TPM values for *WAPO-A1*, -*B1* and -*D1* gene models were obtained from the Wheat eFP Browser at http://bar.utoronto.ca/efp_wheat/cgi-bin/efpWeb.cgi (Winter et al. 2007; Ramírez-González et al. 2018). Heatmaps of the *WAPO-A1*, -*B1* and -*D1* gene profiles were generated using the heatmap.2 function in the gplots package (Warnes et al. 2012) in R (R Core Team, 2014).

**References**

Bray NL, Pimentel H, Melsted P, Pachter L. Near-optimal probabilistic RNA-seq quantification. Nat Biotechnol. 2016 May;34(5):525-7. doi: 10.1038/nbt.3519.

International Wheat Genome Sequencing Consortium (IWGSC) et al. (2018) Science 361:eaar7191. doi: 10.1126/science.aar7191. Epub 2018 Aug 16.

Li Y, Fu X, Zhao M, Zhang W, Li B, An D, Li J, Zhang A, Liu R, Liu X (2018) A genome-wide view of transcriptome dynamics during early spike development in bread wheat. Sci Rep8:15338. doi: 10.1038/s41598-018-33718-y.

R Core Team (2014) A Language and Environment for Statistical Computing. R Found. Stat. Comput.

Ramírez-González RH, Borrill P, Lang D, Harrington SA, Brinton J, Venturini L, Davey M, Jacobs J, van Ex F, Pasha A, Khedikar Y, Robinson SJ, Cory AT, Florio T, Concia L, Juery C, Schoonbeek H, Steuernagel B, Xiang D, Ridout CJ, Chalhoub B, Mayer KFX, Benhamed M, Latrasse D, Bendahmane A; International Wheat Genome Sequencing Consortium, Wulff BBH, Appels R, Tiwari V, Datla R, Choulet F, Pozniak CJ, Provart NJ, Sharpe AG, Paux E, Spannagl M, Bräutigam A, Uauy C (2018) The transcriptional landscape of polyploid wheat. Science 361:eaar6089. doi: 10.1126/science.aar6089.

Warnes GR, Bolker B, Bonebakker L, Gentleman R, Huber W, et al. (2012) Package ‘gplots’. http://cran.r-project.org.

Winter D, Vinegar B, Nahal H, Ammar R, Wilson GV, Provart NJ (2007) An "Electronic Fluorescent Pictograph" browser for exploring and analyzing large-scale biological data sets. PLoS One 2:e718. doi: 10.1371/journal.pone.0000718.
